# Design, synthesis, and biological evaluation of FXR/ASK1 dual-target modulators

**DOI:** 10.3762/bjoc.22.59

**Published:** 2026-05-20

**Authors:** Xi Zhang, Jingyan Wang, Ziqiang Zhao, Caiyi Wang, Zenghui Ye, Wei-Yuan Ma, Jian-Xing Xu, Fengzhi Zhang

**Affiliations:** 1 School of Pharmacy, Hangzhou Medical College, Hangzhou, 310014, P. R. China

**Keywords:** ASK1 inhibitors, FXR agonists, FXR/ASK1 dual-target modulators, MASH

## Abstract

The dual modulation of FXR and ASK1 is considered a promising therapeutic strategy for metabolic dysfunction-associated fatty liver disease (MAFLD) and its progressive form, metabolic dysfunction-associated steatohepatitis (MASH). GW4064 and GS-4997 are effective regulators for FXR and ASK1, respectively. Through the effective functional group splicing strategy, a new dual-target modulator is designed. Compound **Z8**, which acts on both targets, was found to more potently reduce intracellular lipid droplet accumulation in OA-treated HepG2 cells than the FXR agonist GW4064 and the ASK1 inhibitor selonsertib (GS-4997).

## Introduction

The pathogenesis of metabolic dysfunction-associated fatty liver disease (MAFLD) is characterized by multifactorial interactions between environmental, genetic, extrahepatic, and intrahepatic factors. These factors can act in a parallel or sequential manner to drive the development of steatosis, inflammation, and fibrosis [1]. MAFLD is a clinical entity that encompasses a spectrum of disease, with metabolic dysfunction-associated steatohepatitis (MASH) denoting a more advanced form [2]. The condition is characterized by steatosis, hepatocyte injury, and inflammation [3], which can progress to fibrosis, cirrhosis and hepatocellular carcinoma (HCC) [4]. Although isolated hepatic steatosis often follows a benign and non-progressive course, untreated MASH carries a significant risk of severe sequelae [5]. These conditions constitute a substantial and growing burden on healthcare systems, yet treatment options remain limited. Presently, resmetirom, a THR-β agonist, stands as the sole pharmacotherapy to have received approval, thus underscoring the critical and as yet unmet medical need in this domain [6].

**Scheme 1:**
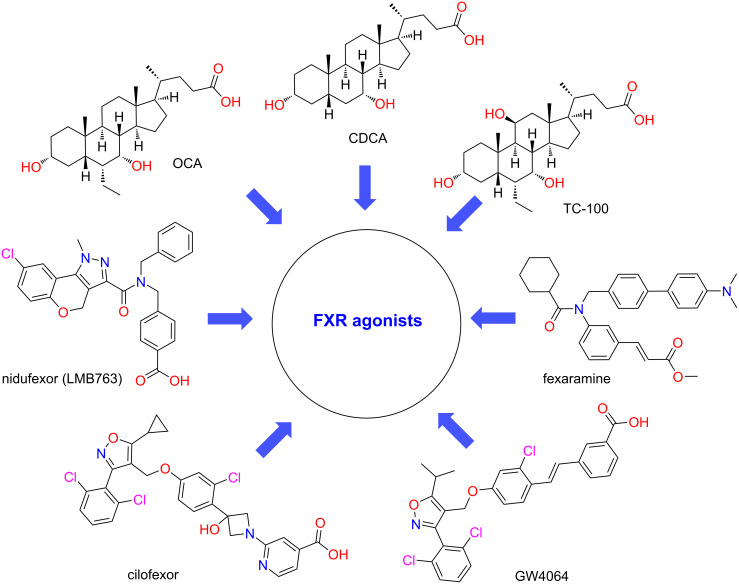
Steroidal and nonsteroidal FXR agonists.

The farnesoid X receptor (FXR), a bile acid-activated nuclear receptor highly expressed in the liver and intestine, is a key regulator of genes involved in cholesterol and bile acid homeostasis, hepatic gluconeogenesis, lipogenesis, inflammation, and fibrosis. It has been demonstrated that the substance under discussion also helps to maintain intestinal barrier integrity, to prevent bacterial translocation, and to support a balanced gut microbiota [7]. In view of this pleiotropic role, FXR activation has emerged as a well-established pharmacological target for MASH [8]. Consequently, a diverse range of FXR agonists – categorized as bile acid derivatives, non-bile-acid steroidal agonists, non-steroidal agonists, and partial agonists – are in advanced clinical development [9–10]. CDCA is the most pharmacologically active FXR agonist in natural bile acids. OCA and TC-100 were derived by a series of derivations using CDCA as a lead compound. Among the non-steroidal agonists, GW 4064, a classical isoxazole FXR agonist, served as the lead compound for a series of derivatives, such as cilofexor (GS-9674). Unfortunately, these agonists have failed in clinical trials because of side effects such as itching or a failure to meet primary endpoints. Despite the failure of several FXR agonists to achieve the desired outcomes, recent research has shifted towards investigating the potential of dual FXR agonists. A number of dual-targeting drugs, which engage FXR in conjunction with other pathways, have demonstrated considerable potential in enhancing the efficacy of MASH treatment outcomes [11–12] (Scheme 1).

**Scheme 2:**
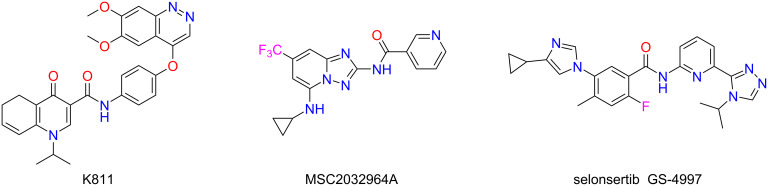
ASK1 inhibitors.

Apoptosis signal-regulating kinase 1 (ASK1), a member of the MAP3K family, is known to be activated by various cellular stressors. These can be induced by diverse factors, spanning from reactive oxygen species (ROS) and endoplasmic reticulum stress to calcium influx and inflammatory agents (e.g., TNF and LPS) [13–14]. One such factor is the activation of ASK1, a complex process that involves homodimerization and autophosphorylation. Two major aspects characterize the role of ASK1 in liver pathophysiology. First, its phosphorylation initiates a signaling relay that activates both JNK and p38 MAPK pathways, resulting in apoptotic cell death, production of proinflammatory cytokines, and fibrogenic gene activation. Second, clinical data reveal that this same signaling node is overactive in the livers of individuals with obesity or MASH, where its functional output correlates directly with the severity of insulin resistance, inflammation, and hepatic steatosis [15]. The most advanced ASK1 inhibitor in clinical development is the compound celonsertib (Scheme 2). Following this, a series of new ASK1 inhibitors were derived using selonsertib as a lead compound in a variety of structure-optimized ways. Given its central role, there are several dual-target drugs being developed that inhibit ASK1 alongside other pathways as antifibrotic agents for MASH treatment [16–17].

ASK1 activation has been demonstrated to drive key pathological processes in the liver, including hepatocyte dysfunction, apoptosis, inflammation, and myofibroblast activation [18–20]. Conversely, intestinal FXR agonism has been demonstrated to promote the release of fibroblast growth factor 19 (FGF19) [21–22], which in turn has been shown to downregulate hepatic bile acid synthesis, lipogenesis, and gluconeogenesis [23–25]. A previous study demonstrated that in a liver fibrosis model and a chronic fast food diet (FFD)-induced MASH mouse mode [26–27], the combination of an ASK1 inhibitor and an FXR agonist led to a more pronounced reduction in hepatic fibrosis than either monotherapy [28].

**Scheme 3:**
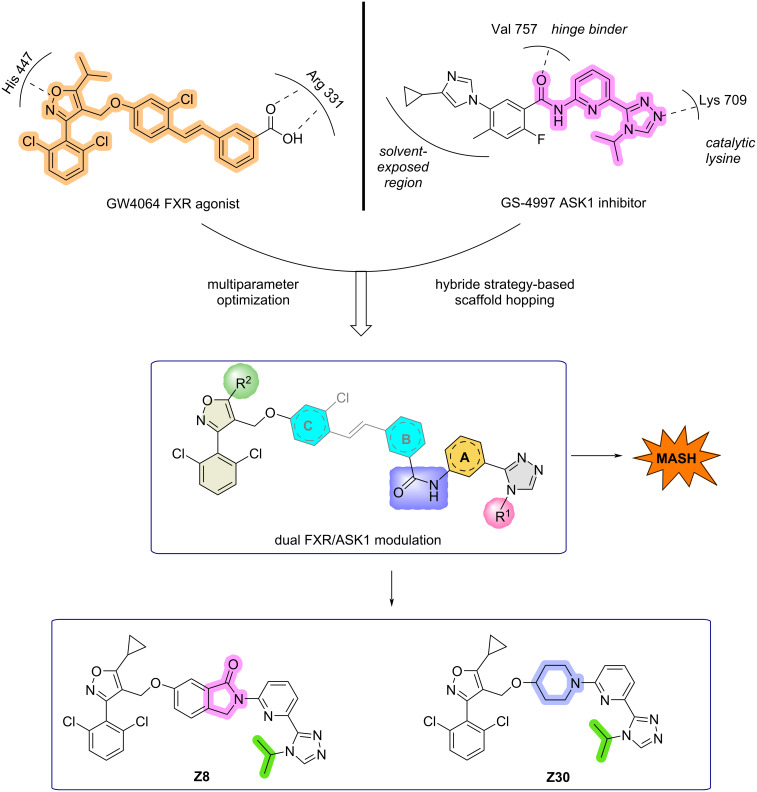
Dual FXR/ASK1 modulation strategy for MASH.

Recently, several publications reported many dual-target drug molecules based on FXR and other relevant therapeutic targets, such as FXR/sEH (soluble epoxide hydrolase), FXR/PPARδ (peroxisome proliferators activated receptor δ), FXR/LTA4H (leukotriene A4 hydrolase), and FXR/FABP 1 (fatty acid binding protein 1) [12,29–31]. In this study, we present the rational design and characterization of a balanced dual modulator targeting the nuclear receptors FXR and ASK1 [32], with the objective of advancing this experimental therapeutic concept (Scheme 3) [33–34]. The conception of this class of dual ligands was achieved by the merging of the minimal pharmacophores of the selective FXR agonist GW4064 and the selective ASK1 inhibitor selonsertib (GS-4997) [35–37]. Published structure–activity relationship (SAR) studies have identified the isoxazole moiety and terminal carboxylic acid of GW4064 [38], along with the 4-aliphatically substituted 4*H*-1,2,4-triazolopyridine biaryl scaffold of selonsertib [31,39–40], as key pharmacophores. In the co-crystal structure of hFXR-LBD with GW4064 [41], the isoxazole core coordinates the π-cation interaction between His 447 and Trp 469; the 5-isopropyl group is embedded into a hydrophobic pocket formed by Phe 284, Leu 287, Trp 454, and Phe 461; the dichlorophenyl moiety engages in π–π stacking with Phe 329; and the *meta*-positioned carboxy group forms a strong electrostatic interaction with Arg 331. The modification strategies for GW4064 are as follows: The stilbene moiety can be replaced through conformational restriction to reduce potential toxicity. The hydrophobic spacer element offers some flexibility in shape and size, but the linker length must remain conserved. Furthermore, to address the issue of poor aqueous solubility, heteroatoms can be introduced into the aromatic ring systems or the phenyl ring at the 3-position of the isoxazole to enhance hydrophilicity and improve pharmacokinetic properties [42]. Co-crystallization of GS-4997 with ASK1 unveiled key stabilizing interactions in the active site. Specifically, the amide carbonyl forms a hydrogen bond with the backbone amide of Val 757, and concurrently, a triazole nitrogen engages in a hydrogen bond with the amine moiety of Lys 709 [43]. By merging the key pharmacophores of the two drugs and systematically optimizing the resulting bipharmacophoric structure, we ultimately obtained balanced dual-target modulators, including the highly active component **Z8**. Despite a significant decrease in solubility due to its relatively high molecular weight, the compound's overall performance remains excellent. It was demonstrated that, in the absence of receptor overexpression, **Z8** effectively activated FXR and inhibited ASK1 signaling. Furthermore, treatment of hepatic cell lines with **Z8** validated its dual-target engagement in native cellular environments. In view of this favorable profile, **Z8** emerges as a highly promising lead compound for further investigation of dual FXR/ASK1 modulation as a polypharmacological strategy for MASH.

## Results and Discussion

### Chemistry

**Scheme 4:**
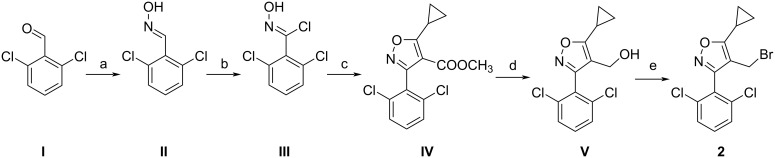
Synthesis of compound **2**. Conditions: (a) NH_2_OH·HCl, NaOH, EtOH/H_2_O, 70 °C, 12 h; (b) NCS, DMF, 40 °C, 2 h; (c) methyl 3-cyclopropyl-3-oxopropanoate, triethylamine, EtOH, rt, 12 h, 64% yield; (d) LiAlH_4_, THF, 0 °C, 2 h, 68% yield; (e) PBr_3_, DCM, 0 °C, 12 h, 79% yield.

**Scheme 5:**
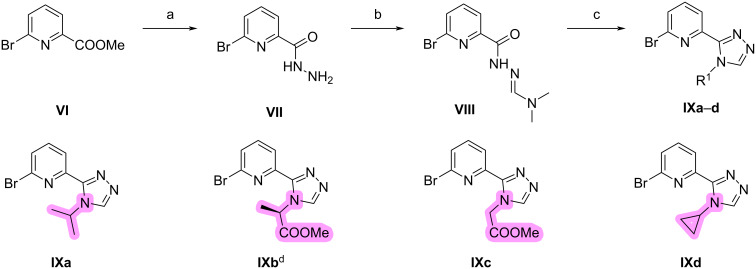
Synthesis of compounds **IXa**–**d**. Conditions: (a) N_2_H_4_·H_2_O, MeOH, rt, 12 h; (b) DMF–DMA, 80 °C, 12 h; (c) amines (for **IXa**,**c**,**d**) or (*R*)-alanine methyl ester (for **IXb**), AcOH, MeCN, 90 °C, 16 h, 66–80% yield; (d) absolute stereochemistry is *R*.

**Scheme 6:**
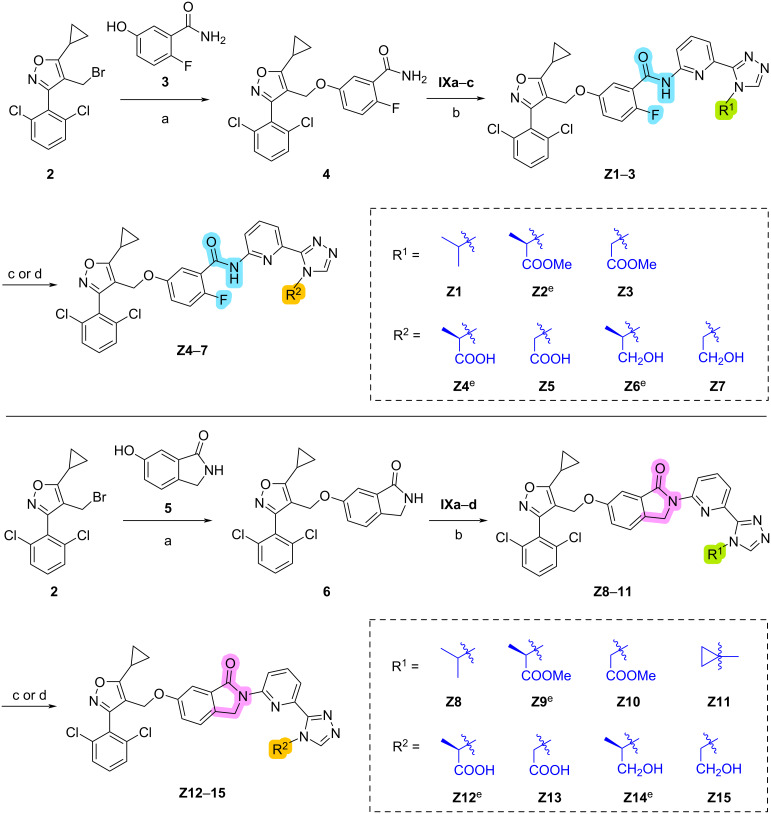
Synthesis of compounds **Z1**–**15**. Conditions: (a) K_2_CO_3_, KI, MeCN, 50 °C, 6 h, 86–96% yield; (b) Pd_2_(dba)_3_, Xantphos, Cs_2_CO_3_, 1,4-dioxane, 80 °C, 12 h, 80–91% yield; (c) LiOH·H_2_O, THF/MeOH/H_2_O, rt, 4 h, 55–76% yield; (d) LiAlH_4_, THF, 0 °C, 4 h, 43–89% yield; (e) absolute stereochemistry is *R*.

**Scheme 7:**
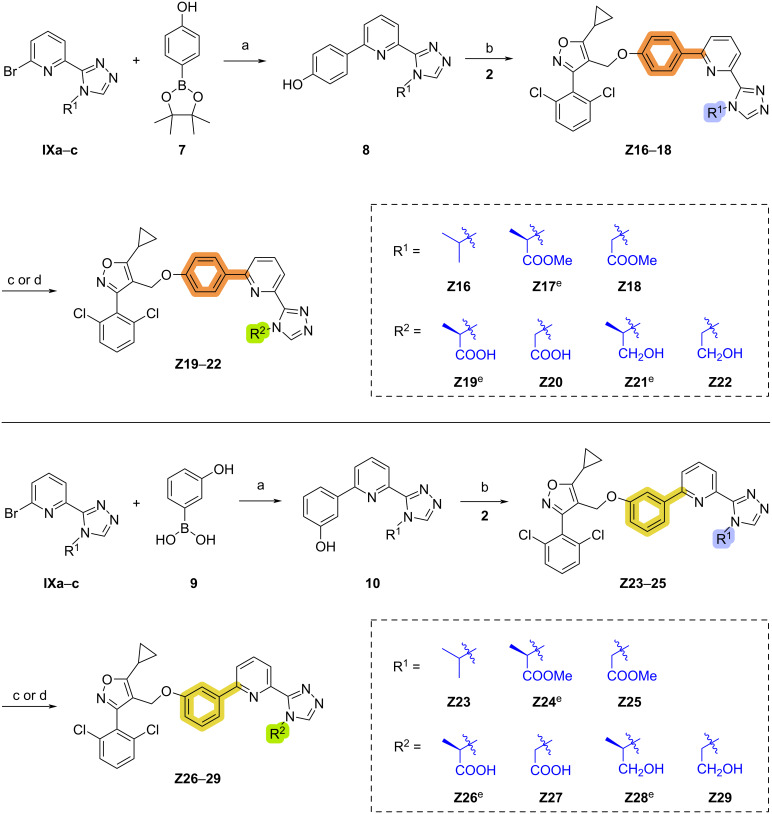
Synthesis of compounds **Z16**–**29**. Conditions: (a) Pd(PPh_3_)_4_, Na_2_CO_3_, 1,4-dioxane/H_2_O, 80 °C, 18 h, 64–90% yield; (b) K_2_CO_3_, KI, MeCN, 50 °C, 6 h, 53–95% yield; (c) LiOH·H_2_O, THF/MeOH/H_2_O, rt, 4 h, 55–76% yield; (d) LiAlH_4_, THF, 0 °C, 4 h, 43–89% yield; (e) absolute configuration is *R*.

**Scheme 8:**
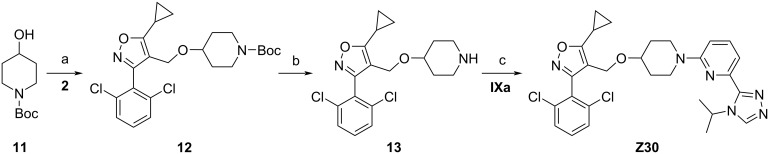
Synthesis of compound **Z30**. Conditions: (a) NaH, DMF, rt, 16 h; (b) TFA, DCM, 0 °C, 3 h; (c) Pd_2_(dba)_3_, Xantphos, Cs_2_CO_3_, 1,4-dioxane, 80 °C, 18 h, 21% yield.

The synthetic routes to the target compounds are outlined in Schemes 4–8. The synthesis of isoxazole intermediate **2** commenced with the condensation of commercially available benzaldehyde **I** with hydroxylamine to afford oxime intermediate **II**. Subsequent treatment of **II** with NCS, followed by cyclization with methyl 3-cyclopropyl-3-oxopropionate, furnished isoxazole **IV**. Reduction of **IV** with lithium aluminum hydride gave alcohol **V**, which was then brominated with phosphorus tribromide to yield the desired isoxazole intermediate **2** in five steps (Scheme 4) [31].

The preparation of triazole intermediates **IXa**–**d** is shown in Scheme 5. Firstly, compound **VI** was reacted with hydrazine hydrate in methanol to obtain hydrazide **VII** which was mixed with DMF/DMA and heated to provide intermediate **VIII**. The obtained **VIII** was not purified and directly reacted with different amines to afford triazoles **IXa**,**c**,**d** or, in case of product **IXb** with (*R*)-alanine methyl ester. The absolute stereochemistry of **IXb** is *R*. All compounds with defined absolute configuration described below were synthesized from **IXb**, thereby preserving the absolute stereochemistry and affording the corresponding *R* enantiomer.

The synthetic routes of compounds **Z1**–**15** are shown in Scheme 6. First, intermediates **2** and **3** were reacted under basic conditions in MeCN to obtain intermediate **4** which was subsequently reacted with bromides **IXa–c** to get compounds **Z1–3** in 80–91% yield. Then, compounds **Z1**–**3** were treated with with LiOH or lithium aluminum hydride to obtain compounds **Z4**–**7**. The synthesis of compounds **Z8**–**15** followed a similar preparation procedure as for **Z1**–**7**.

The synthesis of target compounds **Z16**–**29** is illustrated in Scheme 7. Intermediates **8** and **10** were synthesized by the Suzuki cross-coupling reaction using dioxaborolane **7** or boronic acid **9** and **IXa**–**c**. Next, **Z16**–**18** and **Z23**–**25** were obtained by Williamson ether synthesis from the intermediates **8**, **10** and isoxazole intermediates **2**. Finally, hydrolysis in the presence of LiOH and reduction in the presence of lithium aluminum hydride provided target compounds **Z19**–**22** and **Z26**–**29**.

The synthesis of target compound **Z30** is illustrated in Scheme 8. Treatment of intermediate **2** with *N*-Boc-4-hydroxypiperidine (**11**) in the presence of sodium hydride produced ether **12**, followed by removal of the Boc-protecting group, provided intermediate **13**. Coupling of intermediate **13** with **IXa** under the catalytic conditions of Pd_2_(dba)_3_ provided compound **Z30**.

### Molecular simulation

**Scheme 9:**
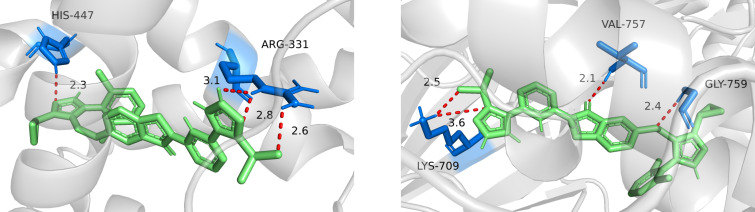
Molecular docking of dual-target modulator **Z8** to the ligand binding sites of FXR (PDB ID: 3DCT, https://doi.org/10.2210/pdb3DCT/pdb) [41] and ASK1 (PDB ID: 6OYT, https://doi.org/10.2210/pdb6OYT/pdb) [44].

To elucidate the binding modes of compound **Z8** with FXR and ASK1, molecular docking simulations were performed as shown in Scheme 9. The results revealed that within the FXR binding pocket, the isoxazole and triazole moieties of **Z8** formed hydrogen bonds with the key residues ARG-331 and HIS-447, respectively. Similarly, in the ASK1 binding pocket, the isoxazole, carbonyl, and triazole groups of **Z8** engaged in hydrogen bonding with residues GLY-759, VAL-757, and LYS-709, respectively. Notably, the binding sites of **Z8** overlapped with those of the reference compounds, GW 4064 and selonsertib, indicating that **Z8** likely engages with FXR and ASK1 through a similar binding mode [45–47].

### Biological evaluations

#### FXR agonist activity

**Figure 1:**
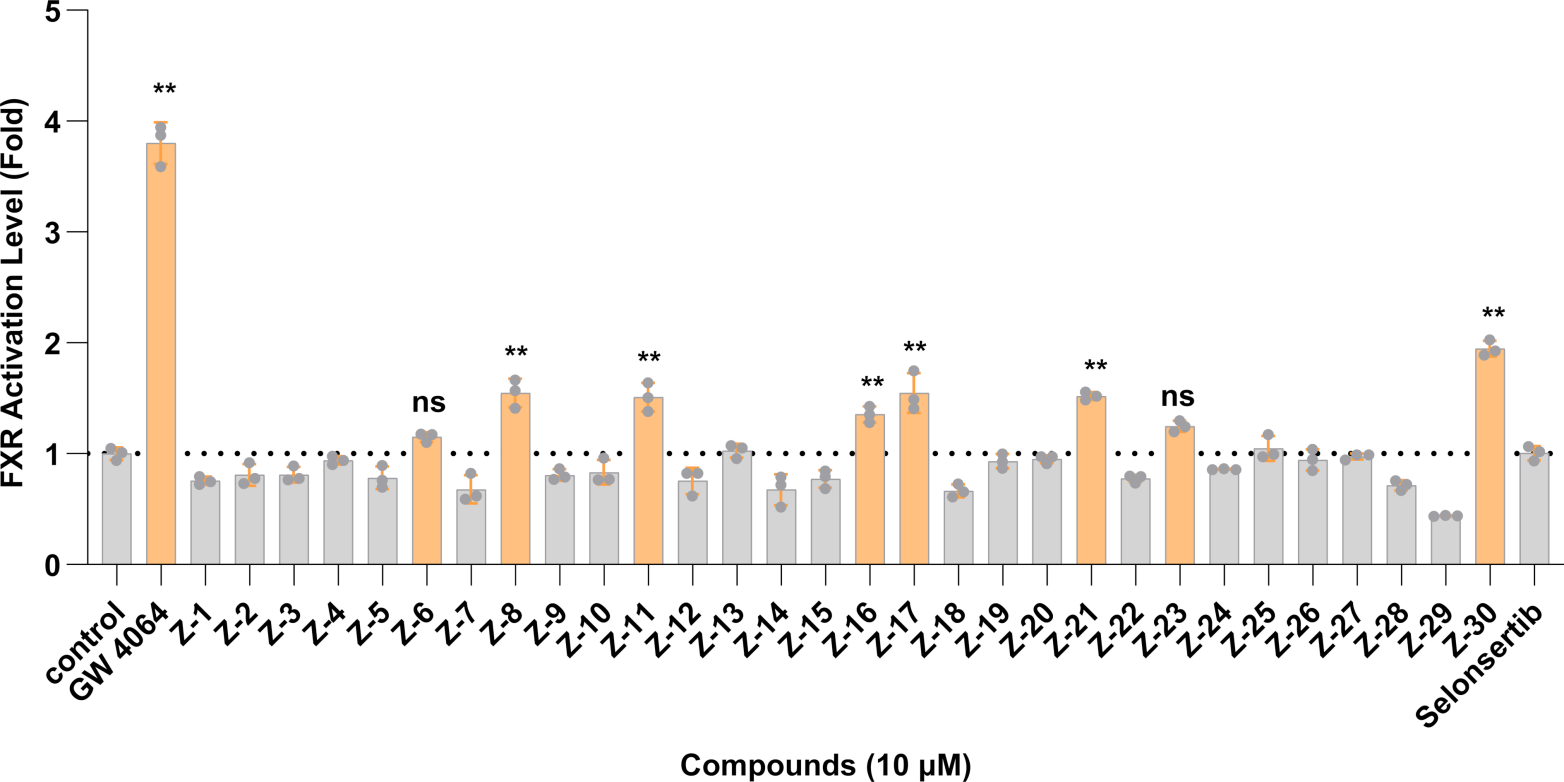
FXR agonist activity of all compounds. Results of the dual-luciferase reporter assay in CHO cells cultured for 24 hours following addition of GW4064 and test compounds. Compared to the control group, **p < 0.01 and ns indicate no significant difference, with control; DMSO serving as the control group.

The evaluation of the FXR agonist activity was conducted through the implementation of a dual-luciferase reporter gene assay. As demonstrated in Figure 1, among the designed dual-target modulators, compounds **Z8**, **Z11**, **Z17**, and **Z30** exhibited the most potent effects at a concentration of 10 μM.

#### ASK1 Inhibition

**Figure 2:**
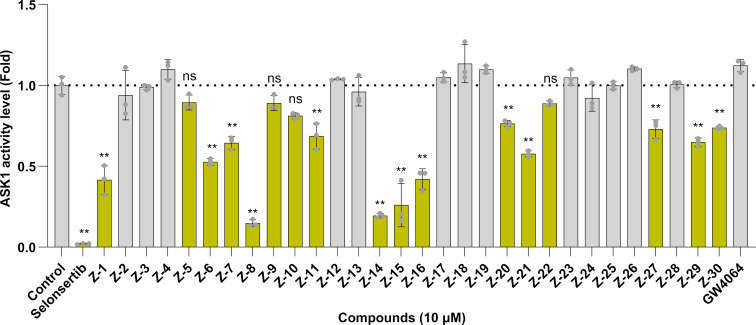
ASK1 inhibition of all compounds**.** Results of the ADP-Glo™ kinase assay after treating ASK1 kinase with selonsertib and test compounds for 1 hour. Compared to the control group: **p < 0.01 and ns indicates no significant difference; control: DMSO control group.

The inhibitory activity against ASK1 was evaluated using the ADP-Glo™ kinase assay. A screen of the designed dual-target modulators identified 17 compounds that inhibited ASK1 (Figure 2). Among the compounds examined, **Z8** demonstrated the greatest potency, exhibiting an inhibition rate of 84.59% at a concentration of 10 μM.

#### Lipid-modifying activity

**Figure 3:**
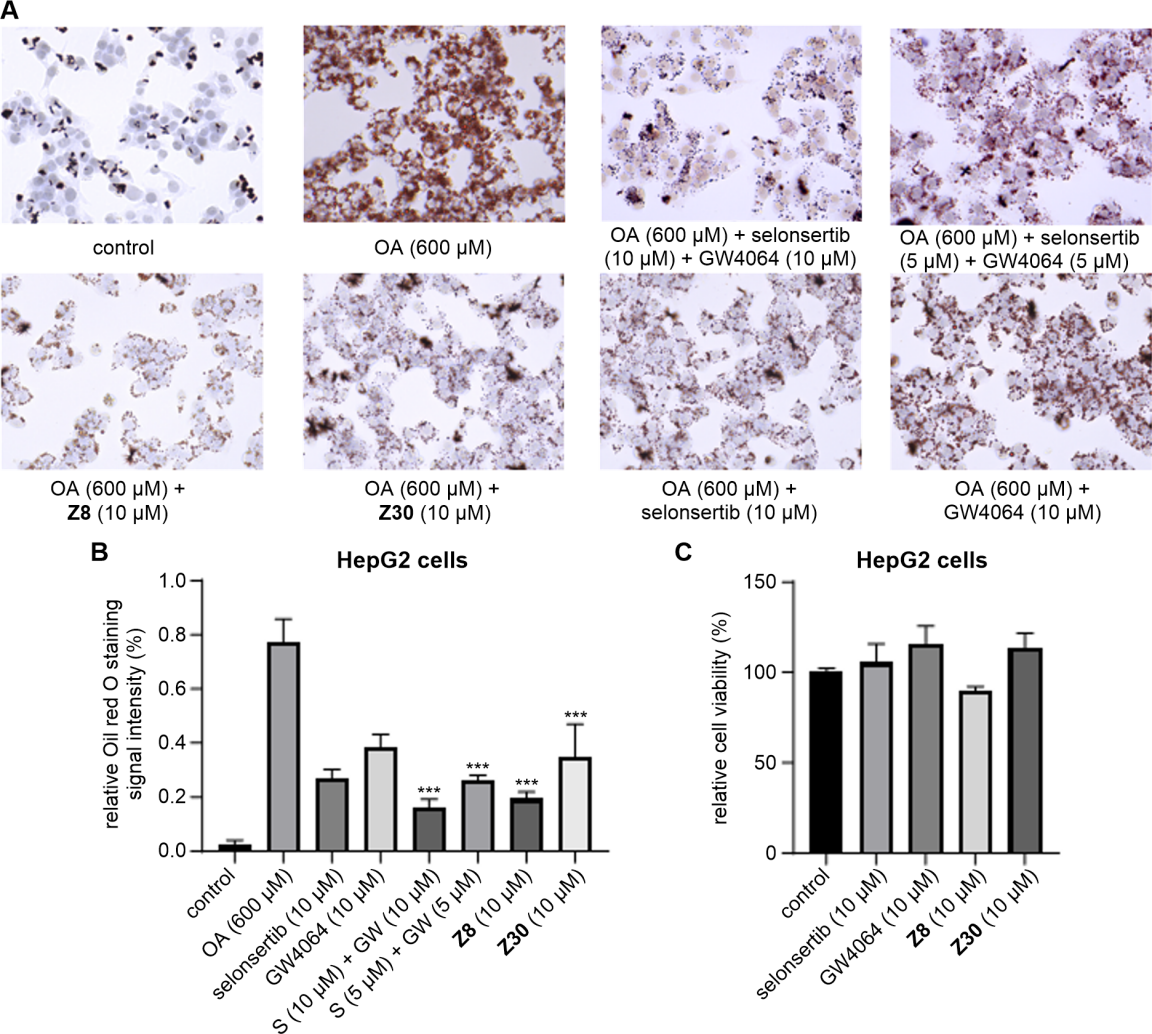
Effects of **Z8** and **Z30** on OA-induced lipid accumulation in HepG2 cells. Cells were treated with GW4064, selonsertib, **Z8**, **Z30**, or the combination of GW4064 and selonsertib for 2 h prior to OA stimulation (600 μM). (A) Representative Oil Red O staining images (40×). (B) Quantification of staining intensity by ImageJ. Data are presented as mean ± SD (*n* = 3). Statistical significance was analyzed by one-way ANOVA followed by the appropriate post hoc test; ***p < 0.001 versus OA control. Additional pairwise comparisons between **Z8** and the positive-control groups are described in the text. (C) Cell viability at 10 μM under the same treatment conditions.

In light of the prevalence of the reduction of lipid levels as a therapeutic objective in MASH, the present study sought to evaluate the lipid-modifying potential of modulators **Z8** and **Z30**. Utilizing an in vitro model of oleic acid (OA)-induced steatosis in HepG2 cells, the study evaluated the effects of these compounds on hepatic lipid accumulation. The presence of lipid droplets was confirmed through direct visualization using Oil Red O staining (Figure 3A). The combined treatment with GW4064 and GS4997 exerted a stronger lipolytic effect than either positive control alone, and its activity was comparable to that of **Z8**. As anticipated, both **Z8** and **Z30** demonstrated a dose-dependent attenuation of OA-induced lipid accumulation. As illustrated in Figure 3B, **Z8** demonstrated significantly higher activity in comparison to the positive controls selonsertib and GW4064. To ascertain whether the viability of the cells was impaired by the compounds, an MTT assay was conducted. The results of this assay demonstrated that neither compound caused a decrease in cell viability at concentrations of up to 10 µM (Figure 3C).

## Conclusion

Given the respective pharmacological roles of FXR and ASK1 in MASH, we hypothesized that a dual modulator targeting both could potentially serve as an excellent therapeutic agent, concurrently activating FXR and inhibiting ASK1. In this work, a new modulator was designed by hybridizing GW4064 with GS-4997, aiming to overcome the limitations of GW4064 and potentiate the inhibitory activity against ASK1. Following comprehensive multiparameter optimization, compound **Z8** emerged as a lead FXR/ASK1 dual modulator, demonstrating suitable liver microsome stability and high target selectivity. Collectively, compound **Z8** holds great promise for the treatment of MASH.

## Supporting Information

File 1 Experimental, characterization data and copies of spectra.

## Data Availability

All data that supports the findings of this study is available in the published article and/or the supporting information of this article.
