# Supplementary material for: Design, synthesis, and biological evaluation of FXR/ASK1 dual-target modulators
Source: Beilstein J Org Chem. 2026 May 20;22:771–81. doi: 10.3762/bjoc.22.59 (PMC13202473; doi:10.3762/bjoc.22.59)
Supplement: File 1 — Experimental, characterization data and copies of spectra. [file Beilstein_J_Org_Chem-22-771-s001.pdf]

BEILSTEIN JOURNAL OF ORGANIC CHEMISTRY

Supporting Information

for

Design, synthesis, and biological evaluation of FXR/ASK1 dual-target modulators

Xi Zhang, Jingyan Wang, Ziqiang Zhao, Caiyi Wang, Zenghui Ye, Wei-Yuan Ma, Jian-Xing Xu and Fengzhi Zhang

Beilstein J. Org. Chem. **2026,** *22,* 771–781. doi:10.3762/bjoc.22.59

Experimental, characterization data and copies of spectra

License and Terms: This is a supporting information file under the terms of the Creative Commons Attribution License (https://creativecommons.org/licenses/by/4.0). Please note that the reuse, redistribution and reproduction in particular requires that the author(s) and source are credited and that individual graphics may be subject to special legal provisions.
The license is subject to the *Beilstein Journal of Organic Chemistry* terms and conditions: (https://www.beilstein-journals.org/bjoc/terms)

Table of contents

1. Detailed experimental procedures and spectroscopic data for all synthesized compounds S2
2. References S19
3. Oil Red O staining and image analysis S20
4. Dual-luciferase reporter assay (FXR) S20
5. ADP-Glo™ kinase assay (ASK1) S21
6. MTT assay S21
7. Copies of ^{1}H and ^{13}C NMR spectra S22

1. Detailed experimental procedures and spectroscopic data for all synthesized compounds

General information

All target compounds were purified via column chromatography with an elution system of DCM/methanol or PE/EA in different ratios, and their structures were confirmed via ^{1}H nuclear magnetic resonance (NMR), ^{13}C NMR, and liquid chromatograph–mass spectrometry (LC–MS). ^{1}H and ^{13}C NMR spectral data were recorded on a 400 MHz spectrometer (Bruker advance III 400 spectrometer). Liquid chromatography–mass spectrometry (LC–MS) was performed using an Agilent-IQ. Unless otherwise stated, all solvents or reagents were obtained from commercially available materials and used directly without further purification. Column chromatography was performed using 200–300 mesh silica gel. The purities of all the synthesized compounds were >95% determined by analytical HPLC on a Shimadzu LC-20AD system, Diamonsil C-18 column.

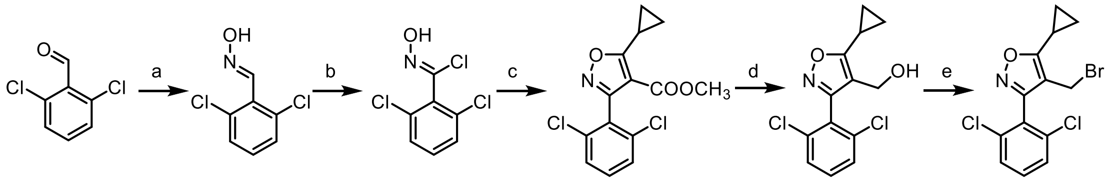

I II III IV V 2

(a) $NH_2OH{\cdot}HCl$, NaOH, EtOH/H_2O, 70 °C, 12 h; (b) NCS, DMF, 40 °C, 2 h; (c) methyl 3-cyclopropyl-3-oxopropanoate, triethylamine, EtOH, r.t., 12 h; (d) $LiAlH_4$, THF, 0 °C, 2 h; (e) PBr_3, DCM, 0 °C, 12 h.

Scheme S1: Synthesis of compound **2**[1]

3 N NaOH (10 mL) was added dropwise to 10 mL of $NH_3O{\cdot}HCl$ (2.16 g, 30 mmol) aqueous suspension to neutralize hydrochloric acid at 0 °C. This mixture was added dropwise to an ethanol solution of **I** (5 g, 28 mmol) at 0 °C and then moved to an oil bath at 70 °C for 12 h to complete the reaction. The reaction mixture was cooled to room temperature and then concentrated to dryness to obtain **II**. NCS (5 g, 28 mmol) in DMF (10 mL) was added dropwise to **II** in DMF at 40 °C. After the reaction was complete, an appropriate amount of water was added to the reaction mixture. The mixture was extracted with ethyl acetate (70 × 3 mL) and concentrated to give **III**. Methyl 3-cyclopropyl-3-oxopropanoate (4 g, 28 mmol) was added to triethylamine (7.9 mL,

56.6 mmol), stirred for 30 min, then added dropwise to **III** in EtOH (100 mL), and reacted overnight at room temperature. After the reaction was completed, the solvent was removed under reduced pressure, water was added, and it was extracted with ethyl acetate and concentrated to obtain **IV**. $LiAlH_4$ (3.2 g, 85 mmol) in anhydrous THF (10 mL) was added dropwise to **IV** in anhydrous THF (100 mL) at 0 °C. The mixture was allowed to warm to room temperature and then stirred for two hours. After the reaction was completed, 15% NaOH (2 mL) was added to quench the reaction. The mixture was filtered, and the filtrate was collected. The organic solvent was removed under reduced pressure, ethyl acetate (50 × 3 mL) was added, and the organic layer was washed three times with water and concentrated to give **V**. PBr_3 (4.7 mL, 28 mmol, 1 equiv) in DCM (5 mL) was added to **V** in DCM (5 mL) slowly at 0 °C. Upon completion of the addition, the reaction mixture was stirred for 12 hours. After the reaction was completed, water was added to quench the reaction. The organic layer was washed three times with saturated Na_2CO_3 (10 mL), concentrated, and purified by column chromatography to obtain 4-(bromomethyl)-5-cyclopropyl-3-(2,6-dichlorophenyl) isoxazole (**2**) [1]. Yield 3.6 g (34%); White solid. **^{1}H NMR** (400 MHz, $CDCl_3$) δ 7.36 (d, *J* = 5.6 Hz, 2H), 7.30 (d, *J* = 7.5 Hz, 1H), 4.15 (s, 2H), 2.07 – 2.03 (m, 1H), 1.37 – 1.25 (m, 2H), 1.14 – 1.05 (m, 2H). **^{13}C NMR** (100 MHz, $CDCl_3$) δ 172.5, 159.1, 135.8, 131.6, 128.3, 127.1, 111.9, 19.9, 8.5, 7.8. **LC-MS (ESI)** calcd for $C_{13}H_{10}BrCl_2NO$ $[M + H]^+$ 345.9, found: 346.0.

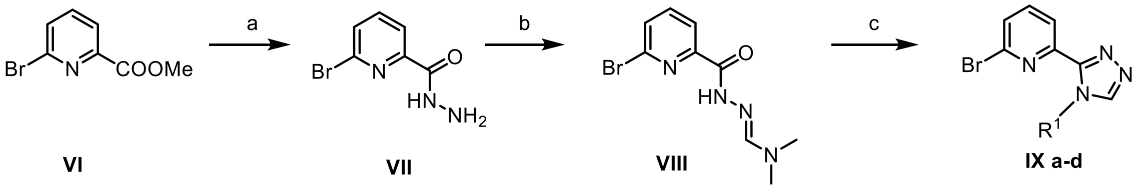

(a) $N_2H_4 \cdot H_2O$, MeOH, RT, 12 h; (b) DMF-DMA, 80 °C, 12 h; (c) amines, AcOH, MeCN, 90 °C, 16 h, 66%-80% yield.

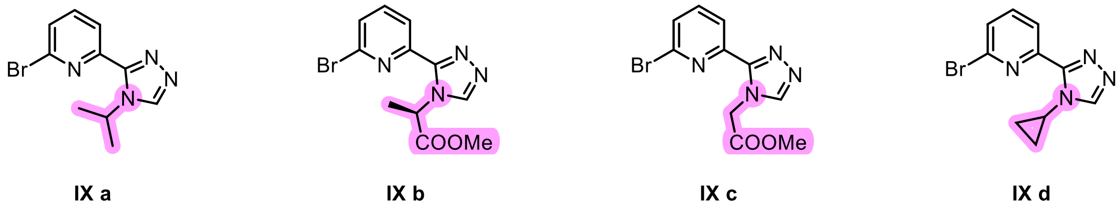

Scheme S2: Synthesis of compounds **IXa–d**. [2]

Hydrazine hydrate (0.3 mL, 6 mmol) was added dropwise to **VI** (0.4 g, 1.9 mmol) in methanol (20 mL) at room temperature for 12 h. After the reaction was completed, the mixture was reduced to 1/3 of its volume under reduced pressure. MTBE (20 mL) was added. The solid was collected by filtration to give **VII** which was used in the next step. DMF-DMA (2.5 mL, 18 mmol) was added to the solid and the reaction mixture was heated at 80 °C overnight. After cooling to room temperature, Et_2O (20 mL) was added. The solid formed was collected by filtration and rinsed with Et_2O to give **VIII** which was used without further purification in the next step. **VIII** (0.5 g, 1.8 mmol) and propan-2-amine (0.8 mL, 9 mmol) were dissolved in acetic acid and MeCN (1 mL:5 mL). The solution was heated at 90 °C for 16 h. After the reaction was completed, the mixture was cooled down and concentrated. The residue was dissolved in water, neutralized with 1 N NaOH and extracted with EA (20 × 3 mL). The organic layer was washed three times with brine (5 × 3 mL), concentrated and purified by column chromatography to obtain 2-bromo-6-(4-isopropyl-4*H*-1,2,4-triazol-3-yl)pyridine (**IXa**).[2] Yield 0.4 g (80%); Pale yellow solid. **LC-MS (ESI)** calcd for $C_{10}H_{11}BrN_4$ $[M + H]^+$ 267.0, found: 266.9.

Methyl 2-(3-(6-bromopyridin-2-yl)-4*H*-1,2,4-triazol-4-yl)propanoate (**IXb)**.[2] Yield 0.4 g (72%); White solid. **^{1}H NMR** (400 MHz, $CDCl_3$) δ 8.42 (s, 1H), 8.36 (d, *J* = 7.2 Hz, 1H), 7.70 (t, *J* = 7.5 Hz, 1H), 7.53 (t, *J* = 7.4 Hz, 1H), 5.92 (d, *J* = 6.6 Hz, 1H), 3.79 (s, 3H), 1.91 (d, *J* = 6.6 Hz, 3H). **^{13}C NMR** (100 MHz, $CDCl_3$) δ 170.6, 149.9, 148.0, 143.7, 140.6, 139.6, 128.6, 122.2, 55.3, 53.1, 18.0. **LC-MS (ESI)** calcd for $C_{11}H_{11}BrN_4O_2$ $[M + H]^+$ 311.0, found: 310.9.

Methyl 2-(3-(6-bromopyridin-2-yl)-4*H*-1,2,4-triazol-4-yl)acetate (**IXc**).[2] Yield 0.8 g (72%); White solid. **^{1}H NMR** (400 MHz, $CDCl_3$) δ 8.37 (d, *J* = 7.5 Hz, 1H), 8.26 (s, 1H), 7.76 – 7.65 (m, 1H), 7.52 (d, *J* = 8.1 Hz, 1H), 5.25 (s, 2H), 3.83 (s, 3H). **^{13}C NMR** (100 MHz, $CDCl_3$) δ 167.5, 150.1, 147.8, 146.6, 140.6, 139.6, 128.6, 121.6, 53.0, 48.2. **LC-MS (ESI)** calcd for $C_{10}H_9BrN_4O_2$ $[M + H]^+$ 296.9, found: 296.9.

2-Bromo-6-(4-cyclopropyl-4*H*-1,2,4-triazol-3-yl)pyridine (**IXd**).[2] Yield 0.2 g (72%); This compound is known, and its NMR spectroscopic data can be found in the cited reference. [2] Pale yellow solid. **LC-MS (ESI)** calcd for $C_{10}H_9BrN_4$ $[M + H]^+$ 265.0, found: 264.9.

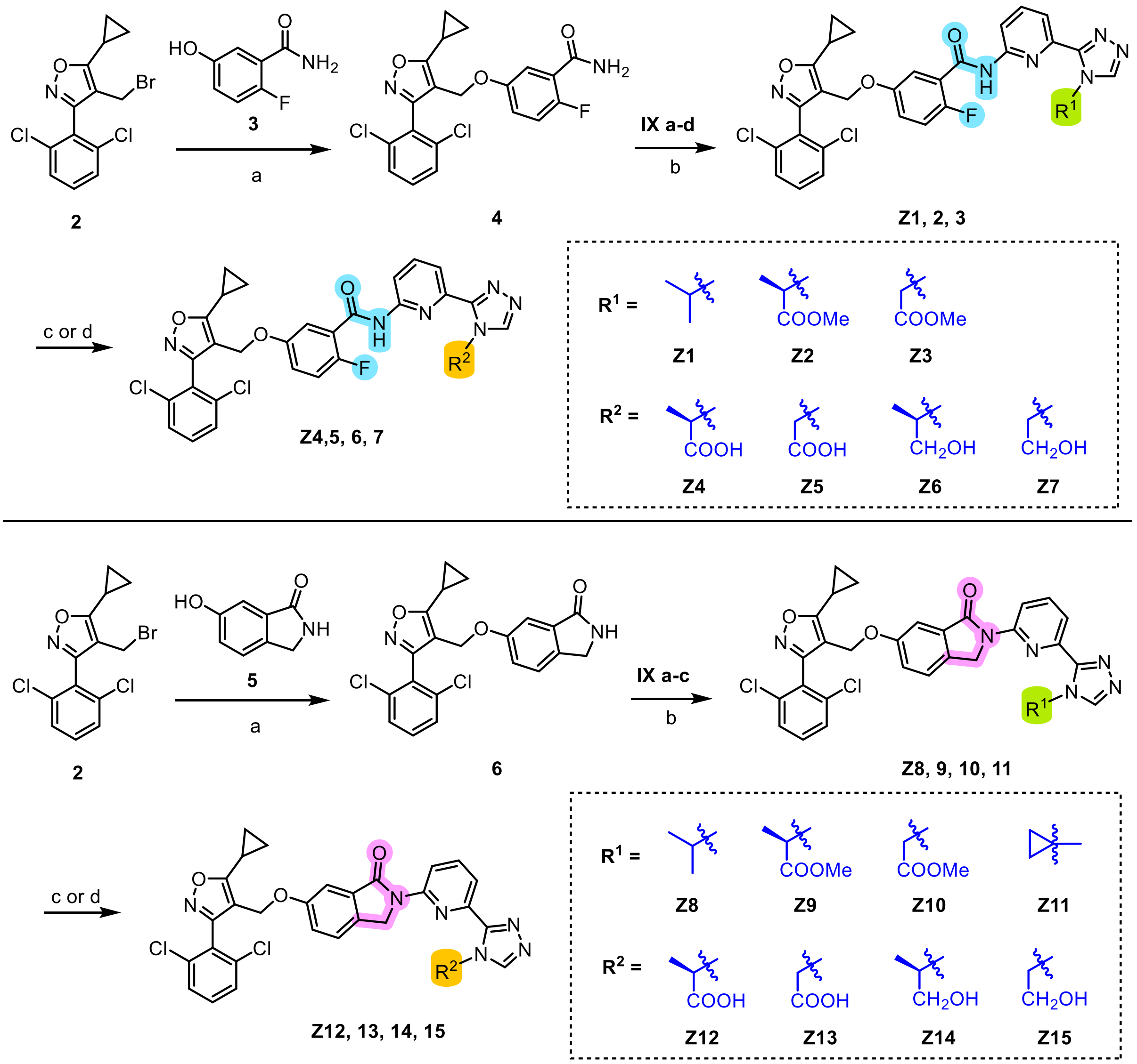

(a) K_2CO_3, KI, MeCN, 50 °C, 6 h, 86%-96% yield; (b) $Pd_2(dba)_3$, Xantphos, Cs_2CO_3, 1,4-dioxane, 80 °C, 12 h, 80%-91% yield; (c) $LiOH{\cdot}H_2O$, THF/MeOH/H_2O, r.t., 4 h, 55%-76% yield; (d) $LiAlH_4$, THF, 0 °C, 4 h, 43%-89% yield.

Scheme S3: Synthesis of intermediates **4** and **6**, and compounds **Z1–15**.

Compound **2** (1.7 g, 5 mmol), **3** (1.6 g, 10 mmol), K_2CO_3 (2 g, 15 mmol), and KI (41 mg, 0.25 mmol) were dissolved in MeCN (20 mL) and the mixture was stirred at 50 °C for 4 h. After the reaction was completed, the reaction mixture was extracted with EA (20 × 3 mL) and purified by column chromatography to obtain 5-((5-cyclopropyl-3-(2,6-dichlorophenyl)isoxazol-4-yl)methoxy)-2-fluorobenzamide (**4**). Yield 1.8 g (86%); White solid. **^{1}H NMR** (400 MHz, $CDCl_3$) δ 7.54 (dd, *J* = 6.1, 3.2 Hz, 1H), 7.42 – 7.36 (m, 2H), 7.31 (dd, *J* = 9.1, 6.9 Hz, 1H), 7.04 – 6.94 (m, 1H), 6.93 – 6.84 (m, 1H), 6.71 (d, *J* = 11.9 Hz, 1H), 6.15 – 6.11 (m, 1H), 4.83 (s, 2H), 2.21 – 2.13 (m, 1H), 1.36 – 1.24 (m, 2H), 1.20 – 1.06 (m, 2H); **^{13}C NMR** (100 MHz, $CDCl_3$) δ 172.6,

164.6, 159.3, 154.6, 135.7, 131.3, 128.1, 127.7, 121.9, 121.8, 120.3, 120.2, 117.3, 117.0, 115.6, 110.2, 60.2, 8.5, 7.8. **LC-MS (ESI)** calcd for $C_{20}H_{15}Cl_2FN_2O_3$ $[M + H]^+$ 421.0, found: 421.0.

6-((5-Cyclopropyl-3-(2,6-dichlorophenyl)isoxazol-4-yl)methoxy)isoindolin-1-one (**6**). Yield 0.4 g (96%); White solid. **^{1}H NMR** (400 MHz, $CDCl_3$) δ 7.59 – 7.44 (m, 1H), 7.43 – 7.36 (m, 2H), 7.33 – 7.28 (m, 3H), 7.01 (dd, *J* = 8.3, 2.4 Hz, 1H), 4.85 (s, 2H), 4.37 (s, 2H), 2.25 – 2.12 (m, 1H), 1.32 – 1.26 (m, 2H), 1.19 – 1.09 (m, 2H). **^{13}C NMR** (100 MHz, $CDCl_3$) δ 175.5, 172.6, 172.1, 159.4, 158.6, 136.3, 135.7, 133.2, 131.3, 128.1, 127.7, 124.1, 121.1, 110.2, 107.4, 59.8, 45.4, 8.5, 7.8. **LC-MS (ESI)** calcd for $C_{21}H_{16}Cl_2N_2O_3$ $[M + H]^+$ 415.0, found: 415.0.

Intermediate **4** (0.1 g, 0.2 mmol) and **IXa** (0.06 g, 0.2 mmol), $Pd_2(dba)_3$ (9.1 mg, 0.01 mmol), Xantphos (17.3 mg, 0.03 mmol) and Cs_2CO_3 (0.13 g, 0.4 mmol) were dissolved in 1,4-dioxane (10 mL) and stirred at 80 °C for 18 h under N_2 atmosphere. After the reaction mixture was completed, the mixture was concentrated under reduced pressure, water was added and extracted with EA (20 × 3 mL). Finally, the organic phase was concentrated and purified by column chromatography to obtain the target product **Z1**. Products **Z2** and **Z3** were synthesized from the corresponding intermediates following the same method.

5-((5-Cyclopropyl-3-(2,6-dichlorophenyl)isoxazol-4-yl)methoxy)-2-fluoro-*N*-(6-(4-isopropyl-4*H*-1,2,4-triazol-3-yl)pyridin-2-yl)benzamide (**Z1**). Yield 0.1 g (83%); White solid. **^{1}H NMR** (400 MHz, $CDCl_3$) δ 9.14 (d, *J* = 16.6 Hz, 1H), 8.44 – 8.36 (m, 2H), 8.06 (d, *J* = 7.7 Hz, 1H), 7.92 (t, *J* = 8.0 Hz, 1H), 7.61 (dd, *J* = 6.2, 3.2 Hz, 1H), 7.40 (d, *J* = 7.9 Hz, 2H), 7.37 – 7.26 (m, 1H), 7.13 – 7.03 (m, 1H), 6.95 (dt, *J* = 8.6, 3.7 Hz, 1H), 5.67 – 5.29 (m, 1H), 4.88 (s, 2H), 2.24 – 2.15 (m, 1H), 1.59 (d, *J* = 6.8 Hz, 6H), 1.32 – 1.26 (m, 2H), 1.21 – 1.14 (m, 2H). **^{13}C NMR** (100 MHz, $CDCl_3$) δ172.6, 161.1, 161.0, 159.3, 156.5, 154.9, 154.1, 150.6, 150.4, 146.3, 142.0, 139.7, 135.7, 131.3, 128.1, 127.6, 122.1, 122.0, 120.9, 120.8, 120.5, 117.5, 117.3, 115.7, 114.8, 110.1, 60.2, 48.8, 23.6, 8.5, 7.8. **LC-MS (ESI)** calcd for $C_{30}H_{25}Cl_2FN_6O_3$ $[M + H]^+$ 607.1, found: 607.2.

Methyl (*R*)-2-(3-(6-(5-((5-cyclopropyl-3-(2,6-dichlorophenyl)isoxazol-4-yl)methoxy)-2-fluoro-benzamido)pyridin-2-yl)-4*H*-1,2,4-triazol-4-yl)propanoate (**Z2**). Yield 0.2 g (64%); White solid. **^{1}H NMR** (400 MHz, $CDCl_3$) δ 9.09 (d, *J* = 13.6 Hz, 1H), 8.46 – 8.37 (m, 2H), 8.16 (d, *J* = 7.7 Hz, 1H), 7.91 (t, *J* = 8.0 Hz, 1H), 7.56 (dd, *J* = 6.0, 3.3 Hz, 1H), 7.40 (d, *J* = 7.3 Hz, 2H), 7.33 (dd, *J*

= 9.1, 6.9 Hz, 1H), 7.11 (dd, *J* = 11.1, 9.0 Hz, 1H), 6.96 (dt, *J* = 9.0, 3.7 Hz, 1H), 5.75 – 5.65 (m, 1H), 4.88 (s, 2H), 3.67 (s, 3H), 2.38 – 2.14 (m, 1H), 1.90 (d, *J* = 7.3 Hz, 3H), 1.33 – 1.27 (m, 2H), 1.19 – 1.14 (m, 2H). **^{13}C NMR** (100 MHz, $CDCl_3$) δ 172.7, 171.2, 161.5, 159.3, 156.4, 154.8, 154.0, 150.6, 150.5, 145.4, 143.7, 139.9, 135.7, 131.3, 128.1, 127.6, 122.1, 122.0, 121.1, 120.9, 119.5, 117.6, 117.3, 115.7, 114.9, 110.1, 60.2, 55.8, 52.9, 17.8, 8.5, 7.8. **LC-MS (ESI)** calcd for $C_{31}H_{25}Cl_2FN_6O_5$ $[M + H]^+$ 651.1, found: 651.1.

Methyl 2-(3-(6-(5-((5-cyclopropyl-3-(2,6-dichlorophenyl) isoxazol-4-yl) methoxy)-2-fluorobenz-amido)pyridin-2-yl)-4*H*-1,2,4-triazol-4-yl)acetate (**Z3**). Yield 0.33 g (66%); White solid. **^{1}H NMR** (400 MHz, $CDCl_3$) δ 9.04 (d, *J* = 14.4 Hz, 1H), 8.45 – 8.39 (m, 1H), 8.24 (s, 1H), 8.19 (dd, *J* = 7.8, 0.9 Hz, 1H), 7.92 (t, *J* = 8.0 Hz, 1H), 7.57 (dd, *J* = 6.1, 3.2 Hz, 1H), 7.44 – 7.39 (m, 2H), 7.32 (dd, *J* = 9.1, 6.9 Hz, 1H), 7.11 (dd, *J* = 11.2, 9.0 Hz, 1H), 6.96 (dt, *J* = 9.0, 3.7 Hz, 1H), 5.20 (s, 2H), 4.88 (s, 2H), 3.71 (s, 3H), 2.24 – 2.13 (m, 1H), 1.33 – 1.16 (m, 2H), 1.20 – 1.09 (m, 2H). **^{13}C NMR** (100 MHz, $CDCl_3$) δ 172.7, 167.9, 161.4, 161.4, 159.3, 156.4, 154.9, 154.0, 150.6, 150.4, 146.3, 145.3, 139.9, 135.7, 131.3, 128.1, 127.6, 122.2, 122.1, 120.9, 120.8, 119.2, 117.5, 117.3, 115.7, 115.1, 110.0, 60.4, 60.2, 52.9, 48.8, 8.50, 7.8. **LC-MS (ESI)** calcd for $C_{30}H_{23}Cl_2FN_6O_5$ $[M + H]^+$ 637.1, found: 637.1.

$LiOH·H_2O$ (4 mg, 0.09 mmol) was added to **Z2** (37 mg, 0.06 mmol) in 3:3:1 THF/MeOH/H_2O (0.9 mL/0.9 mL/0.3 mL). After stirring at room temperature for 6 h, the volatiles were removed under reduced pressure. The residue was acidified with 1 M HCl and extracted with ethyl acetate (20 × 3 mL). The organic fractions were combined and washed with saturated brine prior to drying over anhydrous sodium sulfate. After concentration by using a rotary evaporator, the residue was purified by silica gel column chromatography to afford the target compounds **Z4** and **Z5**).

(*R*)-2-(3-(6-(5-((5-Cyclopropyl-3-(2,6-dichlorophenyl)isoxazol-4-yl)methoxy)-2-fluorobenz-amido)pyridin-2-yl)-4*H*-1,2,4-triazol-4-yl)propanoic acid (**Z4**). Yield 0.02 g (55%); White solid. **^{1}H NMR** (400 MHz, DMSO-d_6) δ 11.05 (s, 1H), 8.63 (s, 1H), 8.18 (d, *J* = 8.2 Hz, 1H), 7.98 (t, *J* = 8.0 Hz, 1H), 7.83 (d, *J* = 7.6 Hz, 1H), 7.62 (d, *J* = 7.9 Hz, 2H), 7.54 (dd, *J* = 9.1, 6.9 Hz, 1H), 7.25 – 7.16 (m, 2H), 6.95 (dt, *J* = 8.5, 3.6 Hz, 1H), 5.93 – 5.46 (m, 1H), 4.94 (s, 2H), 2.48 – 2.45 (m, 1H), 1.59 (d, 3H), 1.19 – 1.13 (m, 2H), 1.13 – 1.08 (m, 2H). **^{13}C NMR** (100 MHz, DMSO-d_6) δ 172.88, 163.6, 159.6, 155.4, 154.2, 152.9, 151.3, 150.8, 147.1, 145.2, 139.8, 135.0, 133.0, 128.9,

127.4, 124.8, 124.7, 119.9, 119.4, 117.7, 117.5, 115.3, 114.4, 110.8, 60.0, 57.0, 20.0, 8.9, 7.7. **LC-MS (ESI)** calcd for $C_{30}H_{23}Cl_2FN_6O_5$ $[M + H]^+$ 637.1, found: 637.1.

2-(3-(6-(5-((5-Cyclopropyl-3-(2,6-dichlorophenyl)isoxazol-4-yl)methoxy)-2-fluorobenz-amido)pyridin-2-yl)-4*H*-1,2,4-triazol-4-yl)acetic acid (**Z5**). Yield 0.02 g (75%); White solid. **^{1}H NMR** (400 MHz, DMSO-d_6) δ 10.81 (s, 1H), 8.48 (s, 1H), 8.15 (d, *J* = 8.2 Hz, 1H), 7.96 (t, *J* = 7.9 Hz, 1H), 7.85 (d, *J* = 7.6 Hz, 1H), 7.61 (d, *J* = 7.9 Hz, 2H), 7.53 (dd, *J* = 9.0, 7.0 Hz, 1H), 7.25 – 7.14 (m, 2H), 6.96 (dd, *J* = 9.0, 3.5 Hz, 1H), 5.75 (s, 2H), 4.94 (s, 2H), 2.50 – 2.46 (m, 1H), 1.21 – 1.12 (m, 2H), 1.16 – 1.09 (m, 2H). **^{13}C NMR** (100 MHz, DMSO-d_6) δ 177.5, 168.2, 164.4, 160.1, 158.9, 157.6, 155.9, 151.7, 144.7, 139.8, 137.6, 133.6, 132.2, 129.4, 129.3, 124.6, 123.3, 122.4, 122.2, 120.0, 118.9, 115.5, 60.1, 50.1, 22.9, 13.6, 12.5. **LC-MS (ESI)** calcd for $C_{29}H_{21}Cl_2FN_6O_5$ $[M + H]^+$ 623.0, found: 623.0.

Compound **Z2** (30 mg, 0.05 mmol) was dissolved in anhydrous tetrahydrofuran (2 mL). Lithium aluminum hydride (3.5 mg, 0.1 mmol) was slowly added under ice-bath cooling, and the mixture was stirred at room temperature for 4 h. After completion of the reaction, the mixture was quenched by the addition of 15% NaOH solution (1 mL) under ice-bath cooling. The resulting mixture was extracted with ethyl acetate (20 mL × 3). The combined organic layers were dried over anhydrous sodium sulfate, concentrated, and purified by column chromatography to afford the target compound **Z6** as a white solid (22 mg, 76% yield).

(*R*)-5-((5-Cyclopropyl-3-(2,6-dichlorophenyl)isoxazol-4-yl)methoxy)-2-fluoro-*N*-(6-(4-(1-hy-droxypropan-2-yl)-4*H*-1,2,4-triazol-3-yl)pyridin-2-yl)benzamide (**Z6**). Yield 0.02 g (76%); White solid. **^{1}H NMR** (400 MHz, $CDCl_3$) δ 9.14 (d, *J* = 16.1 Hz, 1H), 8.33 (d, *J* = 3.9 Hz, 2H), 7.87 – 7.78 (m, 2H), 7.61 – 7.54 (m, 1H), 7.40 (d, *J* = 8.0 Hz, 2H), 7.37 – 7.29 (m, 1H), 7.07 (dd, *J* = 11.3, 9.0 Hz, 1H), 6.95 (dt, *J* = 8.8, 3.9 Hz, 1H), 5.48 – 5.36 (m, 1H), 4.88 (s, 2H), 4.17 – 4.09 (m, 1H), 3.93 – 3.84 (m, 1H), 2.22 – 2.13 (m, 1H), 1.57 (d, *J* = 7.1 Hz, 3H), 1.32 – 1.26 (m, 2H), 1.22 – 1.12 (m, 2H). **^{13}C NMR** (100 MHz, $CDCl_3$) δ 172.7, 161.2, 159.3, 154.9, 154.2, 150.8, 150.3, 145.7, 143.2, 139.8, 135.7, 131.3, 128.1, 127.6, 122.1, 120.9, 120.8, 120.5, 117.3, 115.7, 115.0, 110.1, 65.7, 60.2, 54.2, 17.4, 8.5, 7.8. **LC-MS (ESI)** calcd for $C_{30}H_{25}Cl_2FN_6O_4$ $[M + H]^+$ 623.1, found: 623.1.

5-((5-Cyclopropyl-3-(2,6-dichlorophenyl)isoxazol-4-yl)methoxy)-2-fluoro-*N*-(6-(4-(2-hydroxyethyl)-4*H*-1,2,4-triazol-3-yl)pyridin-2-yl)benzamide (**Z7**). Yield 0.03 g (70%); White solid. **^{1}H NMR** (400 MHz, $CDCl_3$) δ 9.12 (d, *J* = 16.3 Hz, 1H), 8.29 (dd, *J* = 7.9, 1.3 Hz, 1H), 8.16 (s, 1H), 7.84 – 7.72 (m, 2H), 7.56 (dd, *J* = 6.1, 3.2 Hz, 1H), 7.38 (d, *J* = 7.3 Hz, 2H), 7.31 (dd, *J* = 9.1, 6.9 Hz, 1H), 7.06 (dd, *J* = 11.4, 9.0 Hz, 1H), 6.93 (dt, *J* = 9.1, 3.7 Hz, 1H), 4.86 (s, 2H), 4.56 (t, *J* = 4.7 Hz, 2H), 4.10 (t, *J* = 4.7 Hz, 2H), 2.23 – 2.12 (m, 1H), 1.51 – 1.23 (m, 2H), 1.21 – 0.98 (m, 2H). **^{13}C NMR** (100 MHz, $CDCl_3$) δ 172.7, 161.2, 159.3, 156.5, 154.9, 154.1, 150.4, 150.3, 146.5, 145.4, 139.6, 135.7, 131.4, 128.1, 127.6, 122.1, 122.0, 120.9, 120.8, 119.8, 117.6, 117.3, 115.7, 114.9, 110.1, 61.2, 60.2, 49.5, 8.5, 7.8. **LC-MS (ESI)** calcd for $C_{29}H_{23}Cl_2FN_6O_4$ $[M + H]^+$ 609.1, found: 609.1.

6-((5-Cyclopropyl-3-(2,6-dichlorophenyl)isoxazol-4-yl)methoxy)-2-(6-(4-isopropyl-4*H*-1,2,4-triazol-3-yl)pyridin-2-yl)isoindolin-1-one (**Z8**). Yield 0.1 g (89%); White solid. **^{1}H NMR** (400 MHz, $CDCl_3$) δ 8.70 (d, *J* = 8.4 Hz, 1H), 8.39 (s, 1H), 8.04 (d, *J* = 7.6 Hz, 1H), 7.91 (t, *J* = 8.0 Hz, 1H), 7.40 (dd, *J* = 8.1, 3.6 Hz, 3H), 7.37 – 7.30 (m, 2H), 7.07 (dd, *J* = 8.3, 2.5 Hz, 1H), 5.65 – 5.53 (m, 1H), 4.96 (s, 2H), 4.91 (s, 2H), 2.28 – 2.17 (m, 1H), 1.62 (d, *J* = 6.7 Hz, 6H), 1.36 – 1.28 (m, 2H), 1.23 – 1.14 (m, 2H). **^{13}C NMR** (100 MHz, $CDCl_3$) δ 172.5, 167.6, 159.3, 158.8, 151.2, 151.0, 145.9, 141.8, 139.2, 135.7, 133.8, 133.3, 131.3, 128.1, 127.6, 124.0, 122.0, 120.0, 114.6, 110.1, 107.7, 59.9, 49.2, 48.5, 23.8, 8.5, 7.8. **LC-MS (ESI)** calcd for $C_{31}H_{26}Cl_2N_6O_3$ $[M + H]^+$ 601.1, found: 601.1.

Methyl (*R*)-2-(3-(6-(6-((5-cyclopropyl-3-(2,6-dichlorophenyl)isoxazol-4-yl)methoxy)-1-oxoisoindolin-2-yl)pyridin-2-yl)-4*H*-1,2,4-triazol-4-yl)propanoate (**Z9**). Yield 0.1 g (32%); White solid. **^{1}H NMR** (400 MHz, DMSO-d_6) δ 8.93 (s, 1H), 8.59 (d, *J* = 8.4 Hz, 1H), 8.08 (t, *J* = 8.0 Hz, 1H), 7.97 (d, *J* = 7.6 Hz, 1H), 7.62 (d, *J* = 7.4 Hz, 2H), 7.57 – 7.52 (m, 2H), 7.30 (d, *J* = 2.4 Hz, 1H), 7.08 (dd, *J* = 8.4, 2.4 Hz, 1H), 6.33 – 6.23 (m, 1H), 5.75 (s, 2H), 5.03 (s, 2H), 3.69 (s, 3H), 2.57 – 2.53 (m, 1H), 1.87 (d, *J* = 7.4 Hz, 3H), 1.31 – 1.21 (m, 2H), 1.18 – 1.00 (m, 2H). **^{13}C NMR** (100 MHz, DMSO-d_6) δ 172.6, 171.3, 167.4, 159.6, 158.6, 151.3, 150.8, 146.0, 145.1, 140.1, 135.0, 134.5, 133.5, 132.9, 128.8, 127.5, 125.0, 122.0, 119.3, 114.2, 110.8, 108.1, 59.8, 55.3, 54.4, 53.2, 49.2, 18.4, 8.9, 7.7. **LC-MS (ESI)** calcd for $C_{32}H_{26}Cl_2N_6O_5$ $[M + H]^+$ 645.1, found: 645.1.

Methyl 2-(3-(6-(6-((5-cyclopropyl-3-(2,6-dichlorophenyl)isoxazol-4-yl)methoxy)-1-oxoiso-indolin-2-yl)pyridin-2-yl)-4*H*-1,2,4-triazol-4-yl)acetate (**Z10**). Yield 0.1 g (37%); White solid. **^{1}H NMR** (400 MHz, $CDCl_3$) δ 8.71 (d, *J* = 8.5 Hz, 1H), 8.26 (s, 1H), 8.19 (d, *J* = 7.6 Hz, 1H), 7.92 (t, *J* = 8.1 Hz, 1H), 7.41 (d, *J* = 7.1 Hz, 3H), 7.36 – 7.31 (m, 2H), 7.08 (dd, *J* = 8.3, 2.3 Hz, 1H), 5.48 (s, 2H), 4.92 (s, 2H), 4.90 (s, 2H), 3.72 (s, 3H), 2.25 – 2.15 (m, 1H), 1.32 – 1.24 (m, 2H), 1.23 – 1.16 (m, 2H). **^{13}C NMR** (100 MHz, $CDCl_3$) δ 172.6, 167.7, 167.4, 158.8, 151.2, 151.0, 146.1, 145.3, 139.4, 135.7, 133.7, 133.4, 131.3, 128.1, 127.6, 123.9, 122.2, 119.2, 114.9, 110.1, 107.7, 59.9, 52.9, 49.1, 47.6, 8.5, 7.8. **LC-MS (ESI)** calcd for $C_{31}H_{24}Cl_2N_6O_5$ $[M + H]^+$ 631.1, found: 631.1.

6-((5-Cyclopropyl-3-(2,6-dichlorophenyl)isoxazol-4-yl)methoxy)-2-(6-(4-cyclopropyl-4*H*-1,2,4-triazol-3-yl)pyridin-2-yl)isoindolin-1-one (**Z11**). Yield 0.03 g (61%); White solid. **^{1}H NMR** (400 MHz, $CDCl_3$) δ 8.73 (d, *J* = 8.2 Hz, 1H), 8.27 (s, 1H), 7.97 (d, *J* = 7.5 Hz, 1H), 7.92 (t, *J* = 7.9 Hz, 1H), 7.41 (d, *J* = 7.5 Hz, 2H), 7.39 – 7.29 (m, 3H), 7.07 (dd, *J* = 8.3, 2.4 Hz, 1H), 4.99 (s, 2H), 4.90 (s, 2H), 4.07 – 3.78 (m, 1H), 2.27 – 2.13 (m, 1H), 1.35 – 1.29 (m, 2H), 1.23 – 1.16 (m, 2H), 1.15 – 1.06 (m, 2H), 0.98 – 0.94 (m, 2H). **^{13}C NMR** (100 MHz, $CDCl_3$) δ 172.6, 167.7, 159.4, 158.8, 151.3, 145.9, 145.3, 141.9, 139.3, 139.1, 135.8, 133.9, 133.4, 133.3, 131.3, 128.2, 127.7, 127.0, 124.0, 122.1, 120.1, 119.7, 114.7, 110.1, 107.7, 59.9, 49.3, 48.6, 23.9, 8.6, 7.9. **LC-MS (ESI)** calcd for $C_{31}H_{24}Cl_2N_6O_3$ $[M + H]^+$ 599.1, found: 599.1.

(*R*)-2-(3-(6-(6-((5-Cyclopropyl-3-(2,6-dichlorophenyl)isoxazol-4-yl)methoxy)-1-oxoisoindolin-2-yl)pyridin-2-yl)-4*H*-1,2,4-triazol-4-yl)propanoic acid (**Z12**). Yield 0.03 g (88%); white solid. **^{1}H NMR** (400 MHz, DMSO-d_6) δ 8.86 (s, 1H), 8.55 (d, *J* = 8.3 Hz, 1H), 8.02 (t, *J* = 8.0 Hz, 1H), 7.93 (d, *J* = 7.6 Hz, 1H), 7.69 – 7.59 (m, 2H), 7.54 (dd, *J* = 9.1, 7.0 Hz, 1H), 7.49 (d, *J* = 8.4 Hz, 1H), 7.26 (d, *J* = 2.3 Hz, 1H), 7.03 (dt, *J* = 8.4, 2.2 Hz, 1H), 6.04 – 5.98 (m, 1H), 5.14 – 5.03 (m, 1H), 5.01 (s, 2H), 5.00 – 4.92 (m, 1H), 2.56 – 2.52 (m, 1H), 1.83 (d, *J* = 7.4 Hz, 3H), 1.26 – 1.21 (m, 2H), 1.18 – 1.12 (m, 2H). **^{13}C NMR** (100 MHz, DMSO-d_6) δ 172.6, 167.3, 159.6, 158.5, 153.1, 151.2, 146.3, 145.0, 139.9, 135.0, 134.5, 133.5, 132.9, 128.8, 127.5, 124.9, 121.8, 119.2, 113.9, 110.8, 108.1, 59.7, 55.2, 49.4, 18.4, 8.9, 7.7. **LC-MS (ESI)** calcd for $C_{31}H_{24}Cl_2N_6O_5$ $[M + H]^+$ 631.1, found: 631.1.

2-(3-(6-(6-((5-Cyclopropyl-3-(2,6-dichlorophenyl)isoxazol-4-yl)methoxy)-1-oxoisoindolin-2-yl)pyridin-2-yl)-4*H*-1,2,4-triazol-4-yl)acetic acid (**Z13**). Yield 0.01 g (89%); White solid. **^{1}H NMR** (400 MHz, DMSO-d_6) δ 8.62 (s, 1H), 8.50 (dd, *J* = 5.9, 3.4 Hz, 1H), 7.97 (d, *J* = 3.4 Hz, 2H), 7.62 (d, *J* = 8.0 Hz, 2H), 7.54 (dd, *J* = 9.1, 7.1 Hz, 1H), 7.44 (d, *J* = 8.3 Hz, 1H), 7.21 (d, *J* = 2.3 Hz, 1H), 6.97 (dd, *J* = 8.3, 2.4 Hz, 1H), 5.36 (s, 2H), 5.00 (s, 2H), 4.98 (s, 2H), 2.57 – 2.53 (m, 1H), 1.24 – 1.19 (m, 2H), 1.18 – 1.12 (m, 2H). **^{13}C NMR** (100 MHz, DMSO-d_6) δ 172.6, 167.3, 159.6, 158.4, 151.0, 146.7, 139.4, 135.0, 134.6, 133.4, 132.9, 128.8, 127.5, 125.0, 121.6, 118.0, 113.2, 110.8, 107.9, 59.7, 50.4, 49.4, 8.9, 7.7. **LC-MS (ESI)** calcd for $C_{30}H_{22}Cl_2N_6O_5$ $[M + H]^+$ 617.1, found: 617.1.

(*R*)-6-((5-Cyclopropyl-3-(2,6-dichlorophenyl)isoxazol-4-yl)methoxy)-2-(6-(4-(1-hydroxypropan-2-yl)-4*H*-1,2,4-triazol-3-yl)pyridin-2-yl)isoindolin-1-one (**Z14**). Yield 0.1 g (43%); White solid. **^{1}H NMR** (400 MHz, $CDCl_3$) δ 8.61 (dt, *J* = 8.2, 1.4 Hz, 1H), 8.35 (s, 1H), 7.87 – 7.81 (m, 1H), 7.81 – 7.76 (m, 1H), 7.46 – 7.37 (m, 3H), 7.36 – 7.30 (m, 2H), 7.07 (dd, *J* = 8.3, 2.4 Hz, 1H), 5.63 – 5.48 (m, 1H), 5.00 – 4.92 (m, 2H), 4.90 (s, 2H), 4.13 (d, *J* = 11.8 Hz, 1H), 3.94 (dd, *J* = 11.6, 5.5 Hz, 1H), 2.29 – 2.15 (m, 1H), 1.62 (d, *J* = 7.0 Hz, 3H), 1.37 – 1.28 (m, 2H), 1.23 – 1.13 (m, 2H). **^{13}C NMR** (100 MHz, $CDCl_3$) δ 172.6, 167.6, 159.3, 158.8, 151.2, 145.5, 139.2, 135.7, 133.7, 133.3, 131.3, 128.1, 127.6, 124.0, 122.2, 120.1, 114.7, 110.1, 107.7, 59.9, 53.8, 49.4, 17.6, 8.5, 7.8. **LC-MS (ESI)** calcd for $C_{31}H_{26}Cl_2N_6O_4$ $[M + H]^+$ 617.1, found: 617.1.

6-((5-Cyclopropyl-3-(2,6-dichlorophenyl)isoxazol-4-yl)methoxy)-2-(6-(4-(2-hydroxyethyl)-4*H*-1,2,4-triazol-3-yl)pyridin-2-yl)isoindolin-1-one (**Z15**). Yield 0.02 g (50%); White solid. **^{1}H NMR** (400 MHz, $CDCl_3$) δ 8.62 (d, *J* = 8.4 Hz, 1H), 8.23 (s, 1H), 7.89 (d, *J* = 7.5 Hz, 1H), 7.79 (t, *J* = 8.0 Hz, 1H), 7.44 – 7.36 (m, 2H), 7.33 (dt, *J* = 4.3, 2.2 Hz, 2H), 7.08 (dd, *J* = 8.3, 2.1 Hz, 2H), 4.93 (s, 2H), 4.90 (s, 2H), 4.72 (t, *J* = 5.0 Hz, 2H), 4.14 (t, *J* = 5.0 Hz, 2H), 2.21 (ddd, *J* = 8.5, 5.4, 3.3 Hz, 1H), 1.35 – 1.26 (m, 2H), 1.23 – 1.15 (m, 2H). **^{13}C NMR** (100 MHz, $CDCl_3$) δ 172.6, 167.6, 159.3, 158.8, 151.2, 146.3, 146.2, 145.3, 139.1, 135.7, 133.7, 133.3, 131.3, 128.1, 127.6, 124.1, 122.2, 119.3, 114.7, 110.1, 107.7, 61.3, 59.9, 49.4, 49.0, 8.5, 7.8. **LC-MS (ESI)** calcd for $C_{30}H_{24}Cl_2N_6O_4$ $[M + H]^+$ 603.1, found: 603.1.

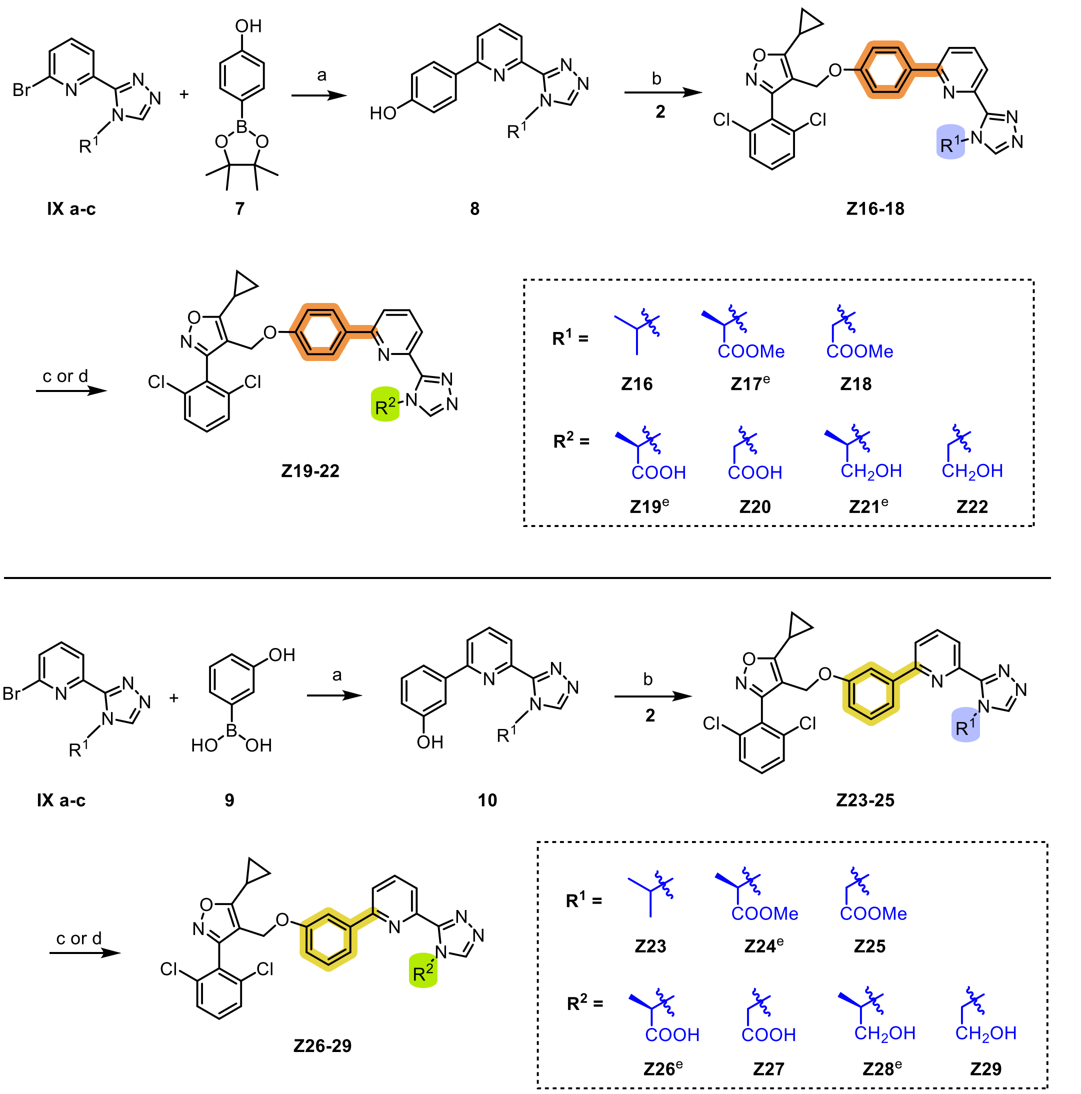

(a) $Pd(PPh_3)_4$, Na_2CO_3, 1,4-dioxane/H_2O, 80 °C, 18 h, 64%-90% yield; (b) K_2CO_3, KI, MeCN, 50 °C, 6 h, 53%-95% yield; (c) $LiOH{\cdot}H_2O$, THF/MeOH/H_2O, r.t., 4 h, 55%-76% yield; (d) $LiAlH_4$, THF, 0 °C, 4 h, 43%-89% yield.(e) Absolute configuration is R.

Scheme S4: Synthesis of intermediates **8**, **10** and compounds **Z16–29**

Compound **IXa** (0.266 g, 1 mmol), **7** (0.330 g, 1.5 mmol), Na_2CO_3 (0.318 g, 3 mmol), and $Pd(PPh_3)_4$ (0.058 g, 0.05 mmol) were dissolved in a mixture of 1,4-dioxane/H_2O 4:1 (2.5 mL/0.625 mL) and the reaction mixture was stirred at 80 °C for 18 h under N_2 atmosphere. Then, the organic phase was removed and extracted with ethyl acetate (20 mL × 3). Finally, the solvent was removed under reduced pressure and the residue purified by column chromatography to obtain intermediates **8**.

The synthetic methods for products **10**, and **Z16**–**Z29** are similar to those mentioned earlier.

5-Cyclopropyl-3-(2,6-dichlorophenyl)-4-((4-(6-(4-isopropyl-4*H*-1,2,4-triazol-3-yl)pyridin-2-yl)phenoxy)methyl)isoxazole (**Z16**). Yield 0.1 g (95%); White solid. **^{1}H NMR** (400 MHz, $CDCl_3$) δ 8.40 (s, 1H), 8.18 (d, *J* = 7.7 Hz, 1H), 7.88 (d, *J* = 8.5 Hz, 2H), 7.84 (d, *J* = 7.8 Hz, 1H), 7.71 (d, *J* = 7.9 Hz, 1H), 7.40 (d, *J* = 7.4 Hz, 2H), 7.32 (dd, *J* = 9.0, 7.0 Hz, 1H), 6.93 (d, *J* = 8.8 Hz, 2H), 5.88 – 5.68 (m, 1H), 4.88 (s, 2H), 2.41 – 2.06 (m, 1H), 1.60 (d, *J* = 6.8 Hz, 6H), 1.55 – 1.23 (m, 2H), 1.23 – 0.83 (m, 2H). **^{13}C NMR** (100 MHz, $CDCl_3$) δ 172.6, 159.3, 159.3, 156.1, 151.3, 147.7, 141.9, 141.9, 137.9, 137.5, 135.8, 131.9, 131.3, 130.2, 128.1, 128.0, 127.7, 127.4, 121.9, 119.9, 115.0, 110.4, 59.5, 48.7, 23.8, 8.4, 7.8. **LC-MS (ESI)** calcd for $C_{29}H_{25}Cl_2N_5O_2$ $[M + H]^+$ 546.1, found: 546.1.

Methyl (*R*)-2-(3-(6-(4-((5-cyclopropyl-3-(2,6-dichlorophenyl)isoxazol-4-yl)methoxy)phenyl)pyridin-2-yl)-4*H*-1,2,4-triazol-4-yl)propanoate (**Z17**). Yield 0.1 g (88%); White solid. **^{1}H NMR** (400 MHz, $CDCl_3$) δ 8.45 (s, 1H), 8.26 (d, *J* = 7.8 Hz, 1H), 7.90 – 7.79 (m, 3H), 7.68 (d, *J* = 8.0 Hz, 1H), 7.41 (d, *J* = 8.0 Hz, 2H), 7.36 – 7.30 (m, 1H), 6.93 (d, *J* = 8.4 Hz, 2H), 6.48 (q, *J* = 7.5 Hz, 1H), 4.88 (s, 2H), 3.62 (s, 3H), 2.26 – 2.15 (m, 1H), 1.91 (d, *J* = 7.5 Hz, 3H), 1.35 – 1.25 (m, 2H), 1.19 – 1.10 (m, 2H). **^{13}C NMR** (100 MHz, $CDCl_3$) δ 172.6, 170.6, 159.4, 159.3, 156.3, 151.2, 147.4, 143.3, 138.0, 135.8, 131.7, 131.3, 128.2, 128.1, 127.7, 121.7, 120.4, 114.9, 110.3, 59.5, 54.5, 52.8, 18.7, 8.4, 7.8. **LC-MS (ESI)** calcd for $C_{30}H_{25}Cl_2N_5O_4$ $[M + H]^+$ 590.1, found: 590.2.

Methyl 2-(3-(6-(4-((5-cyclopropyl-3-(2,6-dichlorophenyl)isoxazol-4-yl)methoxy)phenyl)pyridin-2-yl)-4*H*-1,2,4-triazol-4-yl)acetate (**Z18**). Yield 0.1 g (65%); Yellow solid. **^{1}H NMR** (400 MHz, $CDCl_3$) δ 8.29 (d, *J* = 7.8 Hz, 1H), 8.25 (d, *J* = 1.4 Hz, 1H), 7.85 (td, *J* = 7.9, 1.4 Hz, 1H), 7.81 – 7.74 (m, 2H), 7.66 (d, *J* = 8.0 Hz, 1H), 7.45 – 7.38 (m, 2H), 7.32 (ddd, *J* = 8.8, 7.1, 1.4 Hz, 1H), 6.99 – 6.84 (m, 2H), 5.47 (s, 2H), 4.88 (s, 2H), 3.58 (s, 3H), 2.26 – 2.15 (m, 1H), 1.33 – 1.28 (m,

2H), 1.19 – 1.12 (m, 2H). **^{13}C NMR** (100 MHz, $CDCl_3$) δ 172.5, 171.1, 167.5, 159.3, 159.3, 156.3, 151.4, 147.2, 146.2, 146.1, 138.0, 135.8, 131.8, 131.3, 128.2, 128.1, 127.7, 121.1, 120.5, 114.9, 110.3, 59.5, 52.6, 47.9, 8.4, 7.8. **LC-MS (ESI)** calcd for $C_{29}H_{23}Cl_2N_5O_4$ [M + H]$^+$ 576.1, found: 576.2.

(*R*)-2-(3-(6-(4-((5-Cyclopropyl-3-(2,6-dichlorophenyl)isoxazol-4-yl)methoxy)phenyl)pyridin-2-yl)-4*H*-1,2,4-triazol-4-yl)propanoic acid (**Z19**). Yield 0.02 g (53%); White solid. **^{1}H NMR** (400 MHz, DMSO-d_6) δ 8.84 (s, 1H), 8.05 (d, *J* = 7.6 Hz, 1H), 7.99 (dd, *J* = 8.8, 5.8 Hz, 3H), 7.94 (d, *J* = 7.9 Hz, 1H), 7.62 (d, *J* = 7.9 Hz, 2H), 7.57 – 7.50 (m, 1H), 6.93 (d, *J* = 8.2 Hz, 2H), 6.16 – 6.04 (m, 1H), 4.94 (s, 2H), 2.48 – 2.39 (m, 1H), 1.80 (d, *J* = 7.2 Hz, 3H), 1.22 – 1.17 (m, 2H), 1.16 – 1.12 (m, 2H). **^{13}C NMR** (100 MHz, DMSO-d_6) δ 172.6, 165.8, 159.6, 159.4, 155.8, 151.2, 147.9, 145.1, 138.9, 135.1, 132.9, 131.4, 128.8, 128.6, 127.4, 121.5, 120.4, 115.3, 110.9, 59.3, 55.3, 19.4, 8.8, 7.7. **LC-MS (ESI)** calcd for $C_{29}H_{23}Cl_2N_5O_4$ [M + H]$^+$ 576.1, found: 576.1.

2-(3-(6-(4-((5-Cyclopropyl-3-(2,6-dichlorophenyl)isoxazol-4-yl)methoxy)phenyl)pyr-idin-2-yl)-4*H*-1,2,4-triazol-4-yl)acetic acid (**Z20**). Yield 0.02 g (75%); White solid. **^{1}H NMR** (400 MHz, DMSO-d_6) δ 8.52 (s, 1H), 8.05 – 7.90 (m, 4H), 7.85 (d, *J* = 7.9 Hz, 1H), 7.61 (d, *J* = 7.9 Hz, 2H), 7.52 (t, *J* = 8.1 Hz, 1H), 6.87 (d, *J* = 7.8 Hz, 2H), 5.21 (s, 2H), 4.90 (s, 2H), 2.48 – 2.39 (m, 1H), 1.24 – 1.16 (m, 2H), 1.16 – 1.10 (m, 2H). **^{13}C NMR** (100 MHz, DMSO-d_6) δ 172.5, 159.6, 159.3, 155.7, 151.4, 148.1, 147.7, 138.6, 135.1, 132.9, 131.5, 128.8, 128.6, 127.4, 120.7, 120.0, 115.2, 110.9, 59.2, 49.4, 8.9, 7.7. **LC-MS (ESI)** calcd for $C_{28}H_{21}Cl_2N_5O_4$ [M + H]$^+$ 562.0, found: 562.1.

(*R*)-2-(3-(6-(4-((5-Cyclopropyl-3-(2,6-dichlorophenyl)isoxazol-4-yl)methoxy)phen-yl)pyridin-2-yl)-4*H*-1,2,4-triazol-4-yl)propan-1-ol (**Z21**). Yield 0.04 g (87%); White solid. **^{1}H NMR** (400 MHz, $CDCl_3$) δ 8.37 (s, 1H), 7.94 (d, *J* = 7.7 Hz, 1H), 7.83 – 7.77 (m, 2H), 7.73 (t, *J* = 7.9 Hz, 1H), 7.62 (d, *J* = 8.0 Hz, 1H), 7.44 – 7.38 (m, 2H), 7.35 – 7.29 (m, 1H), 6.92 (d, *J* = 8.5 Hz, 2H), 5.66 – 5.58 (m, 1H), 4.87 (s, 2H), 4.19 – 4.11 (m, 1H), 3.88 (m, 1H), 2.24 – 2.16 (m, 1H), 1.58 (d, *J* = 7.0 Hz, 3H), 1.33 – 1.28 (m, 2H), 1.18 – 1.11 (m, 2H). **^{13}C NMR** (100 MHz, $CDCl_3$) δ 172.8, 172.6, 159.4, 159.4, 159.3, 156.3, 151.5, 147.1, 143.0, 137.9, 135.7, 131.7, 131.7, 131.3, 131.0, 129.9, 128.1, 127.6, 126.9, 122.1, 120.3, 115.1, 110.3, 65.8, 59.8, 59.5, 54.0, 17.5, 8.4, 8.3, 7.8. **LC-MS (ESI)** calcd for $C_{29}H_{25}Cl_2N_5O_3$ [M + H]$^+$ 562.1, found: 562.1.

2-(3-(6-(4-((5-Cyclopropyl-3-(2,6-dichlorophenyl)isoxazol-4-yl)methoxy)phenyl)pyr-idin-2-yl)-4*H*-1,2,4-triazol-4-yl)ethan-1-ol (**Z22**). Yield 0.03 g (74%); White solid. **^{1}H NMR** (400 MHz, $CDCl_3$) δ 8.22 (s, 1H), 7.94 (d, *J* = 7.7 Hz, 1H), 7.78 (d, *J* = 8.3 Hz, 2H), 7.68 (t, *J* = 7.9 Hz, 1H), 7.59 (d, *J* = 8.0 Hz, 1H), 7.41 (d, *J* = 8.0 Hz, 2H), 7.33 (d, *J* = 8.1 Hz, 1H), 6.92 (d, *J* = 8.4 Hz, 2H), 4.87 (s, 2H), 4.72 (t, *J* = 4.8 Hz, 2H), 4.14 (t, *J* = 4.8 Hz, 2H), 2.24 – 2.16 (m, 1H), 1.33 – 1.25 (m, 2H), 1.20 – 1.11 (m, 2H). **^{13}C NMR** (100 MHz, $CDCl_3$) δ 172.6, 159.4, 159.3, 156.3, 146.9, 137.8, 135.7, 131.7, 131.3, 128.1, 128.1, 127.6, 121.3, 120.2, 115.0, 110.3, 61.4, 59.5, 49.4, 8.4, 7.8. **LC-MS (ESI)** calcd for $C_{28}H_{23}Cl_2N_5O_3$ $[M + H]^+$ 548.1, found: 548.1.

5-Cyclopropyl-3-(2,6-dichlorophenyl)-4-((3-(6-(4-isopropyl-4*H*-1,2,4-triazol-3-yl)pyridin-2-yl)phenoxy)methyl)isoxazole (**Z23**). Yield 0.1 g (75%); White solid. **^{1}H NMR** (400 MHz, $CDCl_3$) δ 8.40 (s, 1H), 8.25 (d, *J* = 7.8 Hz, 1H), 7.90 (t, *J* = 7.9 Hz, 1H), 7.74 (d, *J* = 7.9 Hz, 1H), 7.54 (d, *J* = 7.7 Hz, 1H), 7.46 (t, *J* = 2.1 Hz, 1H), 7.35 (d, *J* = 7.9 Hz, 3H), 7.31 – 7.24 (m, 1H), 6.92 (dd, *J* = 8.1, 2.6 Hz, 1H), 5.74 (p, *J* = 6.8 Hz, 1H), 4.87 (s, 2H), 2.24 – 2.16 (m, 1H), 1.56 (d, *J* = 6.8 Hz, 6H), 1.33 – 1.24 (m, 2H), 1.19 – 1.08 (m, 2H). **^{13}C NMR** (100 MHz, $CDCl_3$) δ 172.7, 159.3, 158.8, 156.2, 151.2, 147.8, 142.0, 140.3, 138.0, 137.4, 135.7, 131.3, 129.9, 128.1, 127.6, 122.7, 120.8, 119.7, 115.8, 113.1, 110.4, 59.4, 48.7, 23.8, 23.8, 8.4, 7.8. **LC-MS (ESI)** calcd for $C_{29}H_{25}Cl_2N_5O_2$ $[M + H]^+$ 546.1, found: 546.1.

Methyl (*R*)-2-(3-(6-(3-((5-cyclopropyl-3-(2,6-dichlorophenyl)isoxazol-4-yl)meth-oxy)phenyl)pyridin-2-yl)-4*H*-1,2,4-triazol-4-yl)propanoate (**Z24**). Yield 0.1 g (95%); White solid. **^{1}H NMR** (400 MHz, $CDCl_3$) δ 8.47 (s, 1H), 8.33 (d, *J* = 7.9 Hz, 1H), 7.90 (t, *J* = 7.9 Hz, 1H), 7.71 (d, *J* = 7.9 Hz, 1H), 7.47 (d, *J* = 7.8 Hz, 1H), 7.42 – 7.33 (m, 4H), 7.31 – 7.25 (m, 1H), 6.91 (dd, *J* = 8.2, 2.5 Hz, 1H), 6.44 (q, *J* = 7.5 Hz, 1H), 4.91 (s, 2H), 3.56 (s, 3H), 2.22 (tt, *J* = 8.4, 5.1 Hz, 1H), 1.87 (d, *J* = 7.5 Hz, 3H), 1.34 – 1.24 (m, 2H), 1.18 – 1.08 (m, 2H). **^{13}C NMR** (100 MHz, $CDCl_3$) δ 172.7, 170.5, 159.3, 158.7, 156.4, 151.0, 147.4, 143.4, 140.0, 138.1, 135.7, 131.2, 129.8, 128.0, 127.6, 126.3, 122.4, 121.3, 119.9, 115.8, 113.3, 110.4, 59.4, 54.5, 52.7, 18.6, 8.4, 7.7. **LC-MS (ESI)** calcd for $C_{30}H_{25}Cl_2N_5O_4$ $[M + H]^+$ 590.1, found: 590.1.

Methyl 2-(3-(6-(3-((5-cyclopropyl-3-(2,6-dichlorophenyl)isoxazol-4-yl)meth-oxy)phenyl)pyridin-2-yl)-4*H*-1,2,4-triazol-4-yl)acetate (**Z25**). Yield 0.1 g (55%); Yellow solid. **^{1}H NMR** (400 MHz, $CDCl_3$) δ 8.37 (d, *J* = 7.8 Hz, 1H), 8.25 (s, 1H), 7.89 (t, *J* = 7.9 Hz, 1H), 7.69 (d, *J* = 8.0 Hz, 1H), 7.44 (d, *J* = 7.8 Hz, 1H), 7.40 – 7.31 (m, 4H), 7.28 – 7.26 (m, 1H), 6.90 (dd, *J* = 8.1, 2.5 Hz, 1H), 5.42 (s, 2H), 4.91 (s, 2H), 3.50 (s, 3H), 2.22 (tt, *J* = 8.4, 5.1 Hz, 1H), 1.31 – 1.27 (m, 2H), 1.16 – 1.11 (m, 2H). **^{13}C NMR** (100 MHz, $CDCl_3$) δ 172.7, 167.4, 159.3, 158.7, 156.5, 151.3, 147.2, 146.2, 140.1, 138.1, 135.8, 131.2, 129.7, 128.1, 127.7, 121.8, 121.4, 119.9, 115.7, 113.5, 110.4, 59.4, 52.5, 47.9, 8.4, 7.8. **LC-MS (ESI)** calcd for $C_{29}H_{23}Cl_2N_5O_4$ $[M + H]^+$ 576.1, found: 576.1.

(*R*)-2-(3-(6-(3-((5-Cyclopropyl-3-(2,6-dichlorophenyl)isoxazol-4-yl)meth-oxy)phenyl)pyridin-2-yl)-4*H*-1,2,4-triazol-4-yl)propanoic acid (**Z26**). Yield 0.02 g (70%); White solid. **^{1}H NMR** (400 MHz, DMSO-d6) δ 8.72 (s, 1H), 8.05 (dd, J = 11.2, 7.6 Hz, 2H), 8.01 – 7.96 (m, 1H), 7.71 – 7.65 (m, 2H), 7.59 (dd, J = 8.0, 4.2 Hz, 2H), 7.50 (t, J = 7.8 Hz, 1H), 7.34 (t, J = 7.4 Hz, 1H), 6.80 (d, J = 7.8 Hz, 1H), 5.98 – 5.85 (m, 1H), 5.18 – 4.96 (m, 2H), 2.61 – 2.54 (m, 1H), 1.78 – 1.61 (m, 3H), 1.18 – 1.08 (m, 4H). **^{13}C NMR** (100 MHz, DMSO-d6) δ 172.6, 159.6, 158.9, 155.6, 148.5, 140.1, 138.9, 135.1, 135.0, 132.8, 130.3, 128.8, 128.8, 127.6, 122.4, 120.9, 120.0, 117.0, 112.6, 111.1, 59.5, 55.3, 39.0, 18.3, 8.9, 7.7. **LC-MS (ESI)** calcd for $C_{29}H_{23}Cl_2N_5O_4$ $[M + H]^+$ 576.1, found: 576.2.

2-(3-(6-(3-((5-Cyclopropyl-3-(2,6-dichlorophenyl)isoxazol-4-yl)methoxy)phen-yl)pyridin-2-yl)-4*H*-1,2,4-triazol-4-yl)acetic acid (**Z27**). Yield 0.02 g (89%); White solid. **^{1}H NMR** (400 MHz, DMSO-d_6) δ 8.51 (s, 1H), 8.10 (d, *J* = 7.7 Hz, 1H), 8.00 (t, *J* = 7.8 Hz, 1H), 7.92 (d, *J* = 7.8 Hz, 1H), 7.69 – 7.62 (m, 2H), 7.60 (d, *J* = 8.0 Hz, 2H), 7.50 (t, *J* = 8.1 Hz, 1H), 7.30 (t, *J* = 7.6 Hz, 1H), 6.75 (d, *J* = 7.9 Hz, 1H), 5.11 (s, 2H), 5.08 (s, 2H), 2.64 – 2.56 (m, 1H), 1.17 – 1.07 (m, 4H). **^{13}C NMR** (100 MHz, DMSO-d_6) δ 172.5, 159.7, 158.8, 155.8, 151.5, 148.5, 147.8, 140.1, 138.7, 135.1, 132.8, 130.2, 128.8, 127.7, 121.6, 120.9, 120.1, 116.9, 112.7, 111.2, 59.5, 50.1, 8.9, 7.7. **LC-MS (ESI)** calcd for $C_{28}H_{21}Cl_2N_5O_4$ $[M + H]^+$ 562.0, found: 562.1.

(*R*)-2-(3-(6-(3-((5-Cyclopropyl-3-(2,6-dichlorophenyl)isoxazol-4-yl)methoxy)phen-yl)pyridin-2-yl)-4*H*-1,2,4-triazol-4-yl)propan-1-ol (**Z28**). Yield 0.02 g (78%); White solid. **^{1}H NMR** (400 MHz, $CDCl_3$) δ 8.38 (s, 1H), 8.05 – 8.01 (m, 1H), 7.79 (td, *J* = 7.9, 1.5 Hz, 1H), 7.67 (d, *J* = 7.9 Hz, 1H), 7.47 (d, *J* = 7.6 Hz, 1H), 7.40 (t, *J* = 2.2 Hz, 1H), 7.40 – 7.31 (m, 3H), 7.31 – 7.23 (m, 1H), 6.91 (dd, *J* = 8.2, 2.5 Hz, 1H), 5.67 – 5.56 (m, 1H), 4.89 (s, 2H), 4.09 (dd, *J* = 11.8, 3.5 Hz, 1H), 3.85 (dd, *J* = 11.8, 6.7 Hz, 1H), 2.26 – 2.15 (m, 1H), 1.56 (d, *J* = 6.7 Hz, 3H), 1.31 – 1.24 (m, 2H), 1.17 – 1.09 (m, 2H). **^{13}C NMR** (100 MHz, $CDCl_3$) δ 172.7, 159.3, 158.9, 156.4, 151.4, 147.3, 142.9, 140.1, 138.1, 135.8, 135.7, 131.3, 129.9, 128.1, 128.0, 127.7, 122.9, 121.2, 119.8, 116.1, 113.2, 110.4, 65.9, 59.51 54.0, 17.6, 8.4, 7.8. **LC-MS (ESI)** calcd for $C_{29}H_{25}Cl_2N_5O_3$ $[M + H]^+$ 562.1, found: 562.1.

2-(3-(6-(3-((5-Cyclopropyl-3-(2,6-dichlorophenyl)isoxazol-4-yl)methoxy)phen-yl)pyridin-2-yl)-4*H*-1,2,4-triazol-4-yl)ethan-1-ol (**Z29**). Yield 0.03 g (60%); White solid. **^{1}H NMR** (400 MHz, $CDCl_3$) δ 8.23 (s, 1H), 8.01 (dd, *J* = 7.5, 1.8 Hz, 1H), 7.73 (t, *J* = 7.8 Hz, 1H), 7.63 (dd, *J* = 7.9, 2.6 Hz, 1H), 7.50 – 7.39 (m, 2H), 7.40 – 7.33 (m, 3H), 7.33 – 7.23 (m, 1H), 6.90 (dd, *J* = 8.2, 2.5 Hz, 1H), 4.90 (s, 2H), 4.71 (t, *J* = 3.3 Hz, 2H), 4.10 (t, *J* = 3.3 Hz, 2H), 2.27 – 2.17 (m, 1H), 1.33 – 1.24 (m, 2H), 1.18 – 1.09 (m, 2H). **^{13}C NMR** (100 MHz, $CDCl_3$) δ 172.8, 172.6, 159.3, 158.8, 156.4, 147.1, 140.1, 137.9, 135.7, 133.4, 131.7, 131.3, 131.0, 129.9, 129.86 128.1, 127.7, 126.9, 122.1, 121.1, 119.8, 115.9, 115.7, 113.5, 110.4, 61.4, 59.9, 49.3, 8.4, 7.8. **LC-MS (ESI)** calcd for $C_{28}H_{23}Cl_2N_5O_3$ $[M + H]^+$ 548.1, found: 548.1.

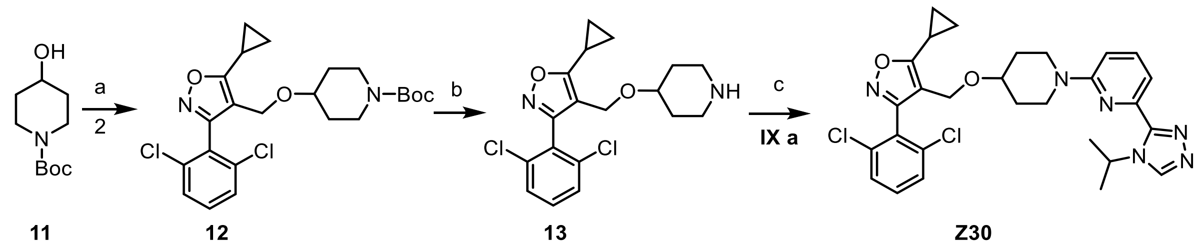

11 12 13 Z30

(a) NaH, DMF, r.t., 16 h; (b) TFA, DCM, 0 °C, 3 h; (c) $Pd_2(dba)_3$, Xantphos, Cs_2CO_3, 1,4-dioxane, 80 °C, 18 h, 21% yield.

Scheme S5: Synthesis of compound **Z30**

Compound **11** (0.3 g, 1.49 mmol) is dissolved in DMF (15 mL). Then, sodium hydride (0.2 g, 8.33 mmol) is gradually added in batches at 0 °C, and the mixture stirred at room temperature for 0.5 h. Then, intermediate **2** (0.5 g, 1.45 mmol) is added and the reaction allowed to proceed at room temperature for 16 h. After the reaction is completed, 10 mL of water are slowly added under an ice bath to quench the reaction. The mother liquor is extracted with ethyl acetate (50 mL × 3), washed with saturated sodium chloride solution, dried with anhydrous sodium sulfate, and concentrated to obtain intermediate **12**. Without further purification the compound is directly used in the next step. Intermediate **12** is dissolved in dichloromethane. Trifluoroacetic acid (0.17 mL, 2.24 mmol) is slowly added under ice-bath cooling, and the mixture stirred at room temperature for 3 h. After the reaction is completed, saturated sodium bicarbonate solution is slowly added under an ice-bath cooling to adjust the pH to neutral, the mixture extracted with dichloromethane (20 mL × 3), dried with anhydrous sodium sulfate, and concentrated to obtain yellow oily intermediate **13** which is directly used in the next step without further purification.

Compound **13** (0.66 g, 1.79 mmol), **IXa** (0.40 g, 1.49 mmol), $Pd_2(dba)_3$ (68 mg, 0.0745 mmol), Xantphos (129 mg, 0.224 mmol) and Cs_2CO_3 (0.97 g, 2.98 mmol) are dissolved in 1,4-dioxane (15 mL) and the mixture stirred at 80 °C for 18 h under N_2 atmosphere. After the reaction is completed, the mixture was concentrated under reduced pressure, water was added and extracted with EA (50 mL × 3). Finally, the organic phase is concentrated and purified by column chromatography to obtain 5-cyclopropyl-3-(2,6-dichlorophenyl)-4-(((1-(6-(4-isopropyl-4*H*-1,2,4-triazol-3-yl)pyridin-2-yl)piperidin-4-yl)oxy)methyl)isoxazole (**Z30**). Yield 0.02 g (21%); colorless oil. **^{1}H NMR** (400 MHz, $CDCl_3$) δ 8.32 (s, 1H), 7.59 (t, *J* = 7.8 Hz, 1H), 7.54 (d, *J* = 7.3 Hz, 1H), 7.38 (d, *J* = 8.0 Hz, 2H), 7.29 (d, *J* = 7.2 Hz, 1H), 6.68 (d, *J* = 8.3 Hz, 1H), 5.63 – 5.51 (m, 1H), 4.35 (s, 2H), 3.72 – 3.62 (m, 2H), 3.56 – 3.44 (m, 1H), 3.28 – 3.16 (m, 2H), 2.21 – 2.10 (m, 1H), 1.82 – 1.68 (m, 2H), 1.52 (d, *J* = 6.7 Hz, 6H), 1.50 – 1.44 (m, 2H), 1.30 – 1.24 (m, 2H), 1.16 – 1.09 (m, 2H). **^{13}C NMR** (100 MHz, $CDCl_3$) δ 171.8, 159.4, 158.4, 151.7, 145.9, 141.5, 138.4, 135.7, 131.1, 128.3, 127.9, 113.3, 111.7, 107.7, 73.5, 58.5, 48.2, 42.6, 29.9, 23.8, 8.3, 7.7. **LC-MS (ESI)** calcd for $C_{28}H_{30}Cl_2N_6O_2$ $[M + H]^+$ 553.1, found: 553.2.

2. References

[1] Ren, Q., Chen, Y., Zhou, Z., Cai, Z., Jiao, S., Huang, W., Wang, B., Chen, S., Wang, W., Cao, Z., Yang, Z., Deng, L., Hu, L., Zhang, L., Li, Z. *J. Med. Chem.*, **2023**, *66*, 6082–6104.

[2] Pang, L., Wang, T., Huang, J., Wang, J., Niu, X., Fan, H., Wan, P., Wang, Z. *Bioorg. Chem.*, **2024**, *144*, 107167.

[3] Tang, L., Li, M., Bai, C., Feng X., Hu H., Yao, Y., Li, B., Li H., Qin, G., Xi N., Lv, G., Zhang, L. *RSC Med. Chem.*, **2024**, *15*, 856-873.

3. Oil Red O staining and image analysis

HepG2 cells were seeded into 12-well plates at 1 mL per well (1 × 10^5 cells/mL) and cultured overnight. When cells reached ≈50–60% confluence, culture medium was replaced. The control group received complete medium only, whereas the model group received OA at a final concentration of 600 µM. For compound-treatment groups, cells were co-incubated with OA (600 µM) and the indicated compounds (Selonsertib, GW4064, **Z8**, or **Z30**; each at 10 µM final concentration). After 24 h of incubation, cells were subjected to downstream assays.

ORO working solution was prepared by mixing ORO stock solution with water at a 3:2 ratio, allowing the mixture to stand briefly, and filtering to remove precipitates. Cells were washed twice with PBS and fixed with 4% paraformaldehyde for 20–30 min at room temperature. After washing, cells were briefly incubated with 60% isopropanol for permeabilization, then stained with ORO working solution for 20–30 min in the dark. Excess dye was removed by repeated washing until the background was clear. Nuclei were counterstained with hematoxylin for ≈1–2 min, followed by rinsing with water. Images were acquired under a light microscope, and ORO-positive lipid staining was quantified using image software (Fiji distribution, NIH, Bethesda, MD, USA; version 20220612).

4. Dual-luciferase reporter assay (FXR)

CHO cells were seeded into 96-well plates and maintained at 37 °C in a humidified incubator with 5% CO_2. Transfection was performed when cells reached ≈50% confluence. The culture medium was replaced with 100 µL serum-free F-12K medium containing pcDNA-Hfxr (50 ng), pGL3-FXRE-luc (50 ng), pRL-TK (10 ng), and HG-TransGene transfection reagent (0.25 µL) per well. After 4 h, the transfection medium was replaced with complete F-12K medium, and cells were incubated for an additional 18 h. Transfected cells were then treated with test compounds at 10 µM for 24 h. Firefly and Renilla luciferase activities were measured using a Dual-Luciferase Reporter Assay kit on a multimode plate reader according to the manufacturer's instructions. Firefly luciferase activity was normalized to the corresponding Renilla luciferase activity to obtain relative FXR agonistic activity.

5. ADP-Glo™ kinase assay (ASK1)

Kinase reaction buffer (40 mM Tris, 20 mM $MgCl_2$, 0.1 mg/mL BSA, and 50 µM DTT) was prepared, and ASK1 kinase, ATP, MBP substrate, and test compounds were diluted in the same buffer as needed. In a 384-well plate, 1 µL of test compound (10 µM) was added to each well, and 1 µL kinase buffer was added to control wells. Subsequently, 2 µL of ASK1 (15 ng) and MBP (0.25 µg/µL) mixture and 2 µL ATP (62.5 µM) were added to each well. The reaction mixture was incubated at room temperature for 1 h. Then, 5 µL ADP-Glo™ reagent was added to terminate the kinase reaction and deplete remaining ATP, followed by incubation at room temperature for 40 min. Next, 10 µL kinase detection reagent was added to convert ADP to ATP and generate a luminescent signal. After equilibration at room temperature for 0.5 h, luminescence was recorded using a plate reader, and kinase activity was calculated accordingly.

6. MTT assay

HepG2 cells were collected into a 15 mL centrifuge tube and pelleted at 1200 rpm for 5 min. After removal of the supernatant, the cell pellet was resuspended in 1–2 mL of complete culture medium, and the cell number was determined. The cell suspension (100 µL) was dispensed into each well of a 96-well plate. To minimize edge effects, peripheral wells were filled with PBS (100 µL/well), and three wells containing culture medium only were included as blanks. Cells were incubated at 37 °C in a humidified atmosphere with 5% CO_2 for 24 h.

Cells were treated with the test compound (10 µM), with a vehicle-only group included as the 0-compound control. Each condition was assayed in triplicate. After incubation at 37 °C in a humidified atmosphere of 5% CO_2 for 24 h, 10 µL of 10% MTT solution was added to each well and the plates were further incubated for 4 h. The supernatant was then carefully aspirated, and 100 µL of DMSO was added to each well to solubilize the formazan crystals by shaking for 10 min. Absorbance (OD) was subsequently measured using a microplate reader.

Cell viability (%) was calculated as:

$$\text{Cell viability (\%)} = (OD_{treated} - OD_{blank}) / (OD_{0\text{-}drug} - OD_{blank}) \times 100\%$$

7. Copies of ^{1}H and ^{13}C NMR spectra

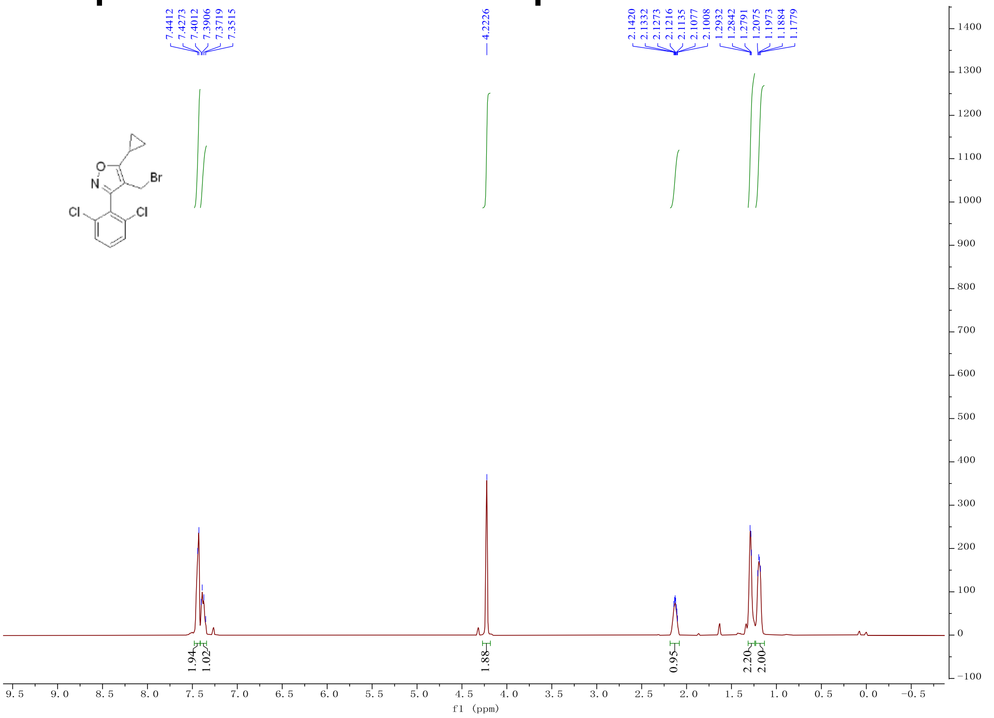

Figure S1: 1H NMR (400 MHz, $CDCl_3$) spectrum of intermediate **2**

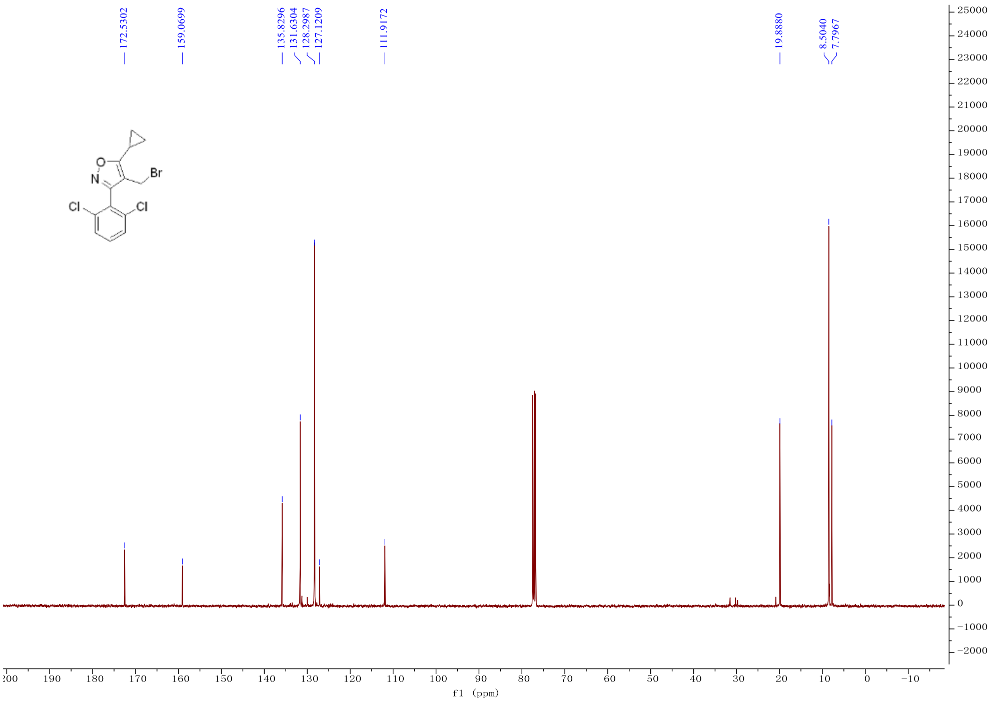

Figure S2: ^{13}C NMR (100 MHz, $CDCl_3$) spectrum of intermediate **2**

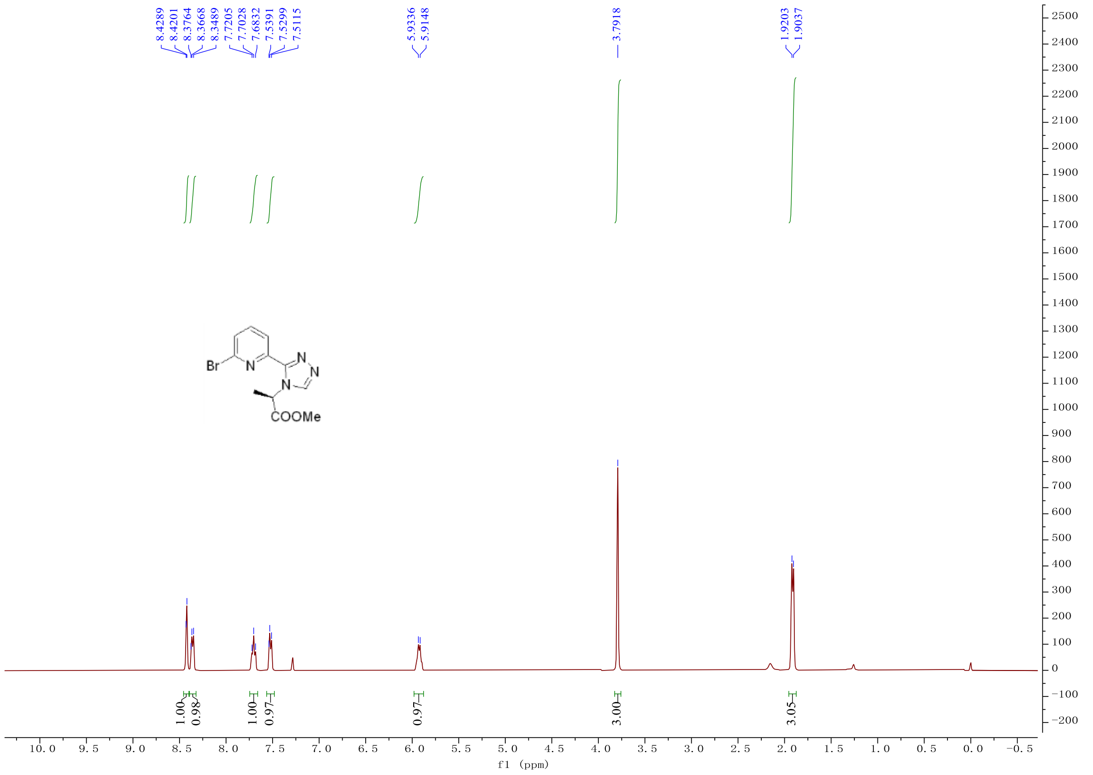

Figure S3: ^{1}H NMR (400 MHz, $CDCl_3$) spectrum of intermediate **IXb**

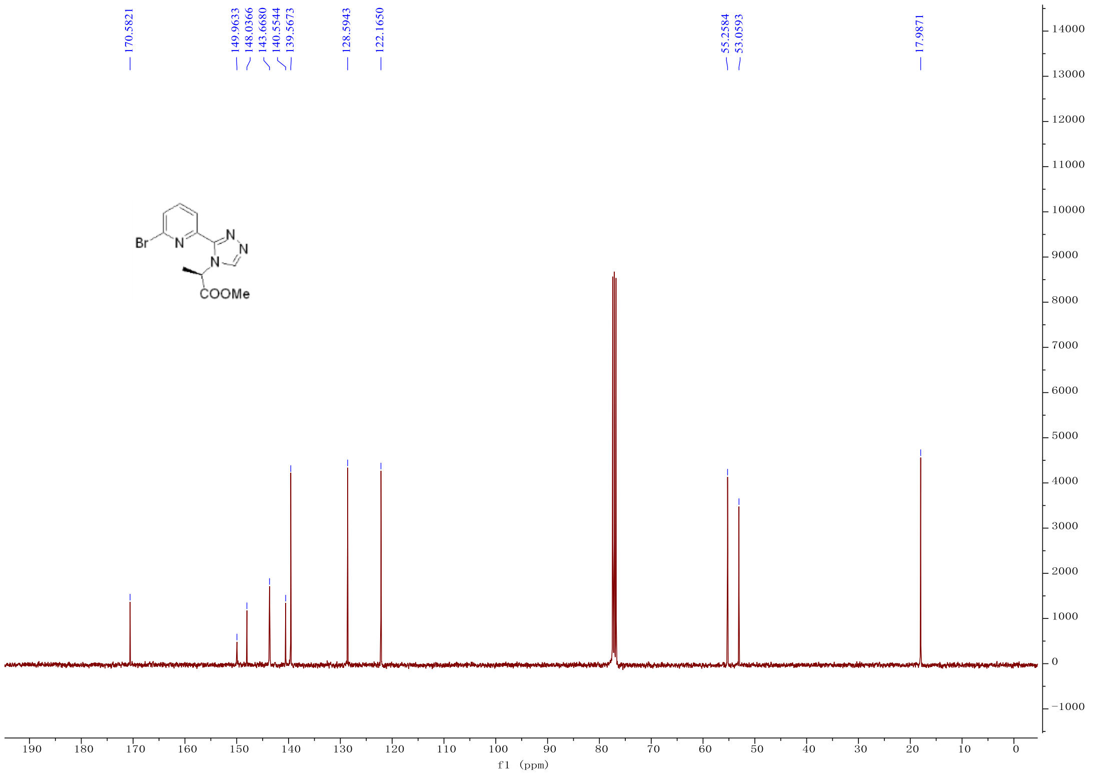

Figure S4: ^{13}C NMR (100 MHz, $CDCl_3$) spectrum of intermediate **IXb**

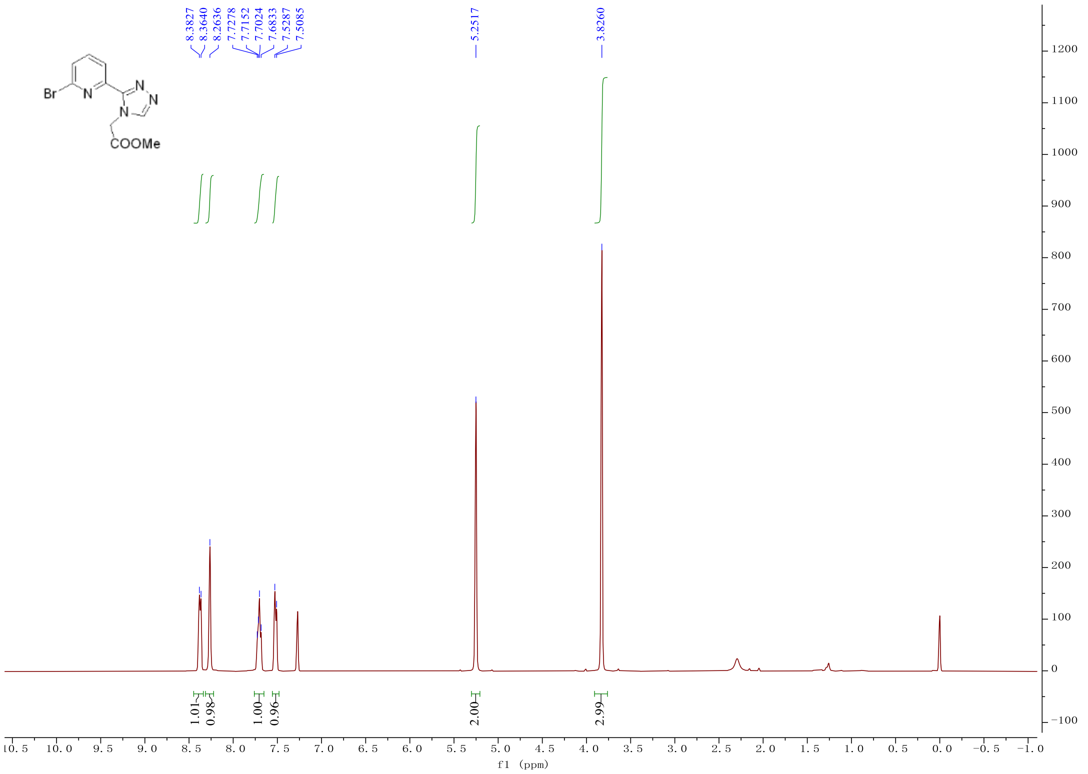

Figure S5: ^{1}H NMR (400 MHz, $CDCl_3$) spectrum of intermediate **IXc**

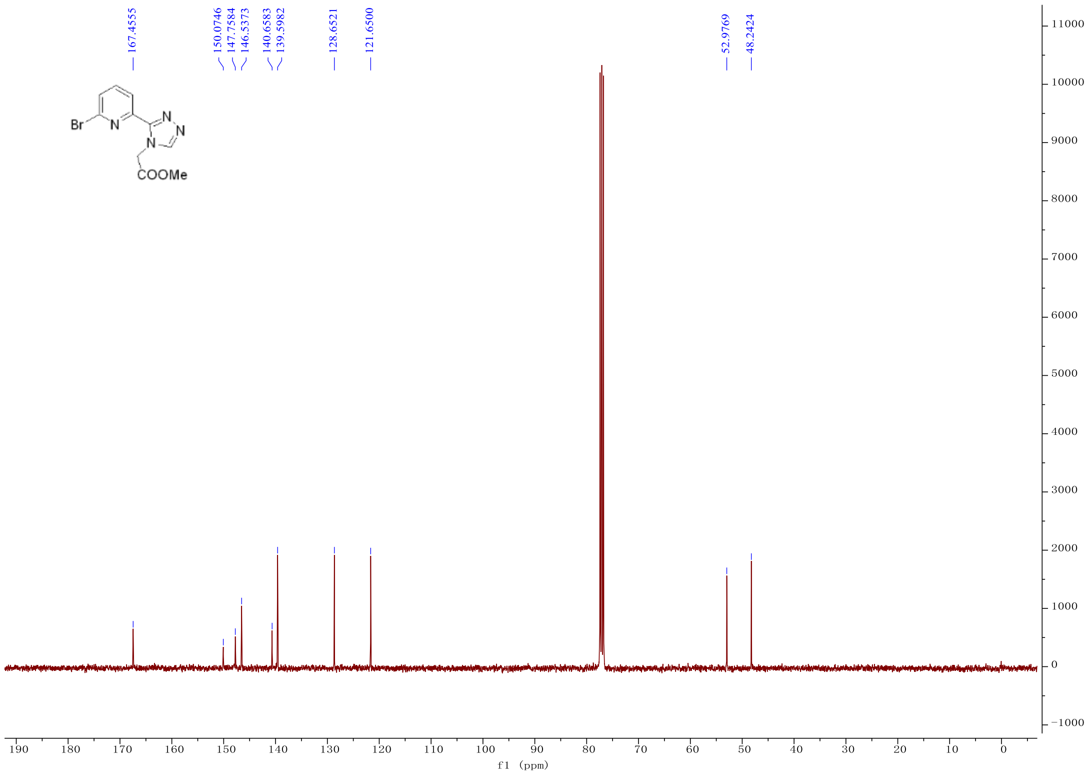

Figure S6: ^{13}C NMR (100 MHz, $CDCl_3$) spectrum of intermediate **IXc**

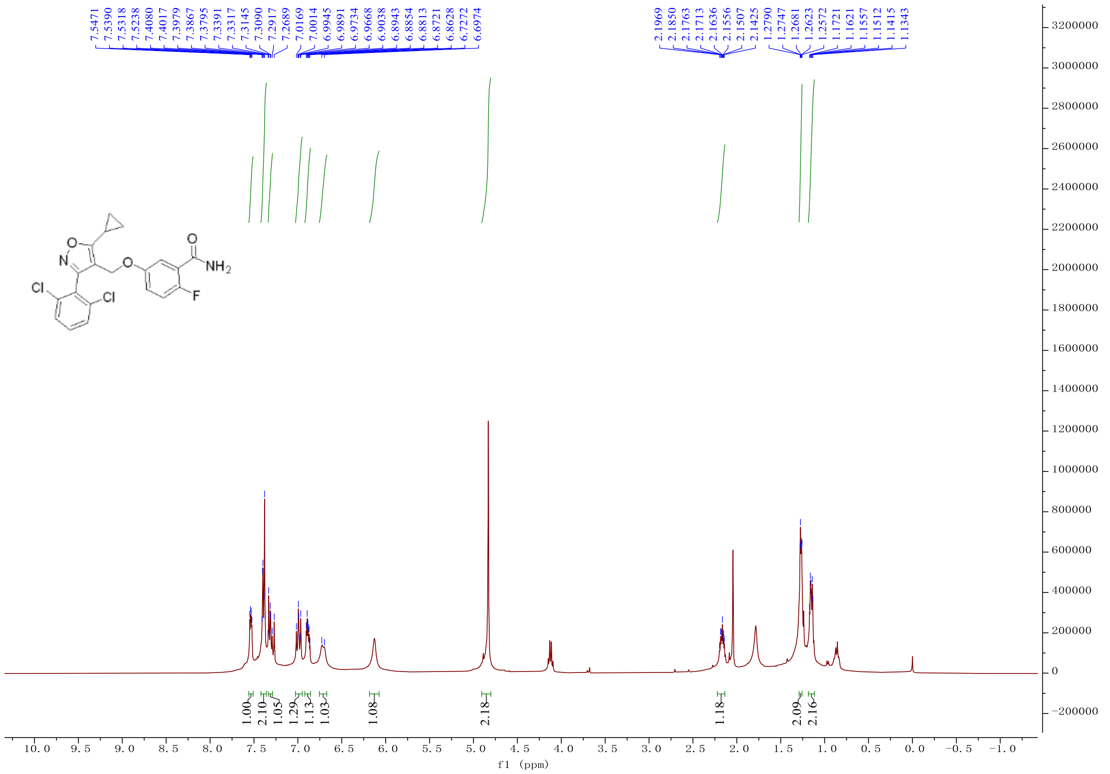

Figure S7: ^{1}H NMR (400 MHz, $CDCl_3$) spectrum of intermediate **4**

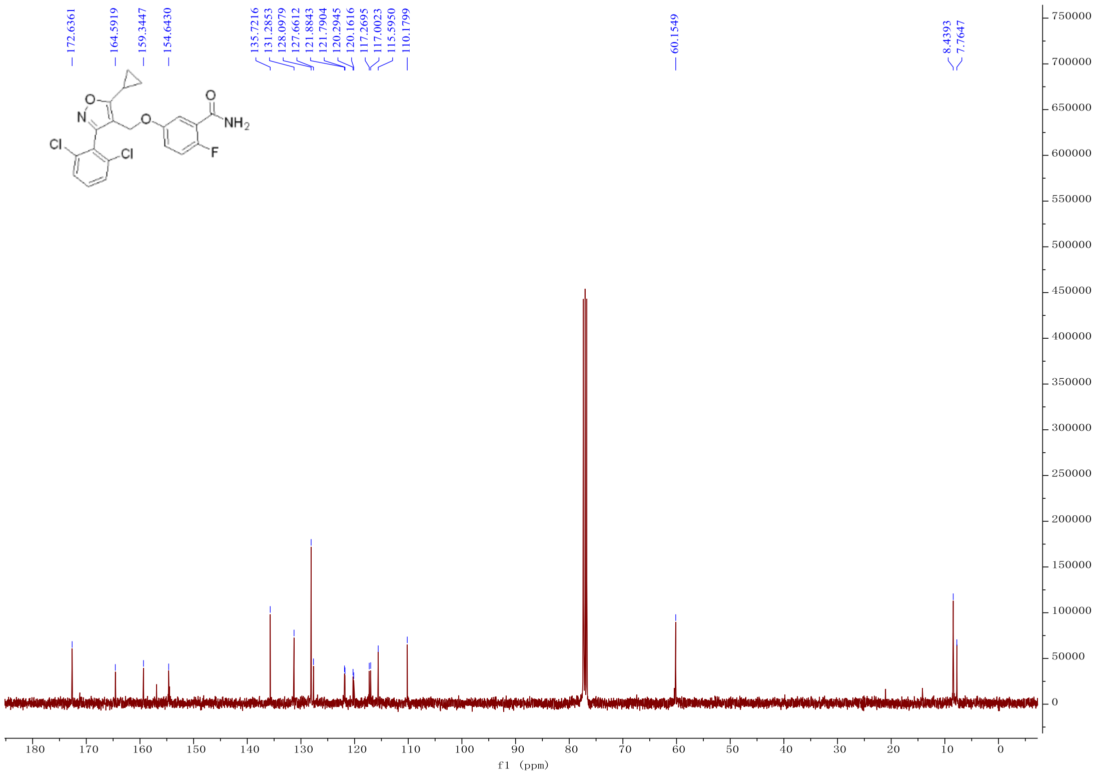

Figure S8: ^{13}C NMR (100 MHz, $CDCl_3$) spectrum of intermediate **4**

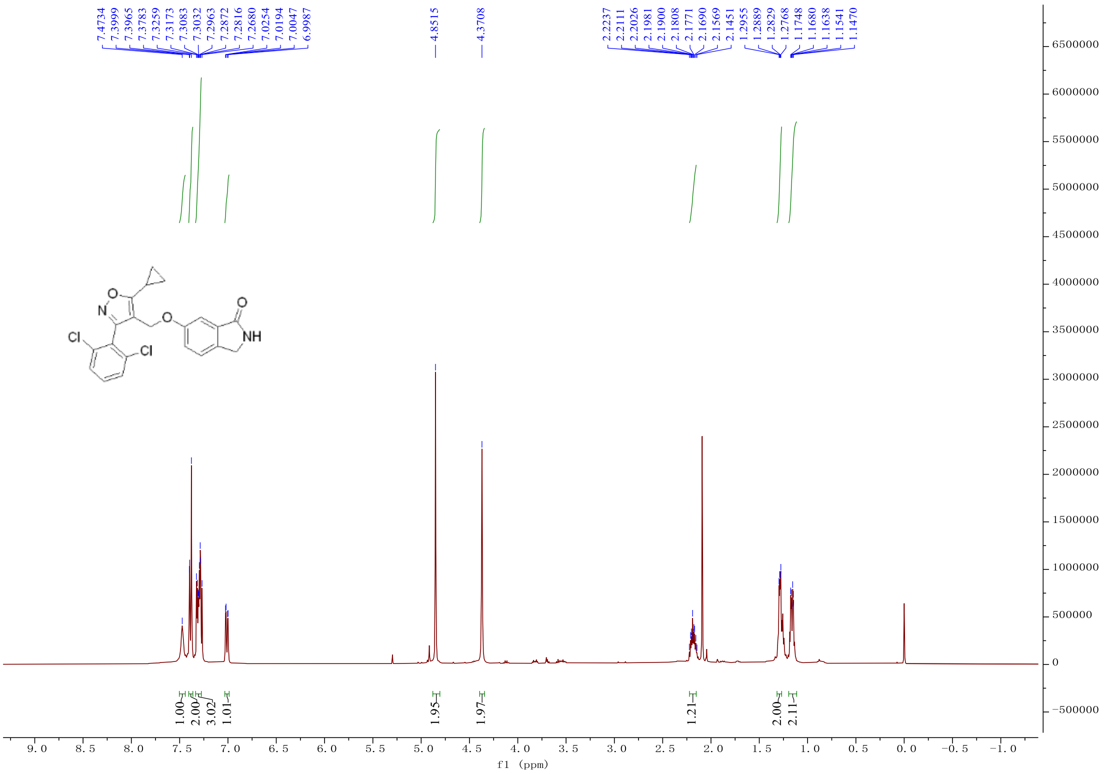

Figure S9: ^{1}H NMR (400 MHz, $CDCl_3$) spectrum of intermediate **6**

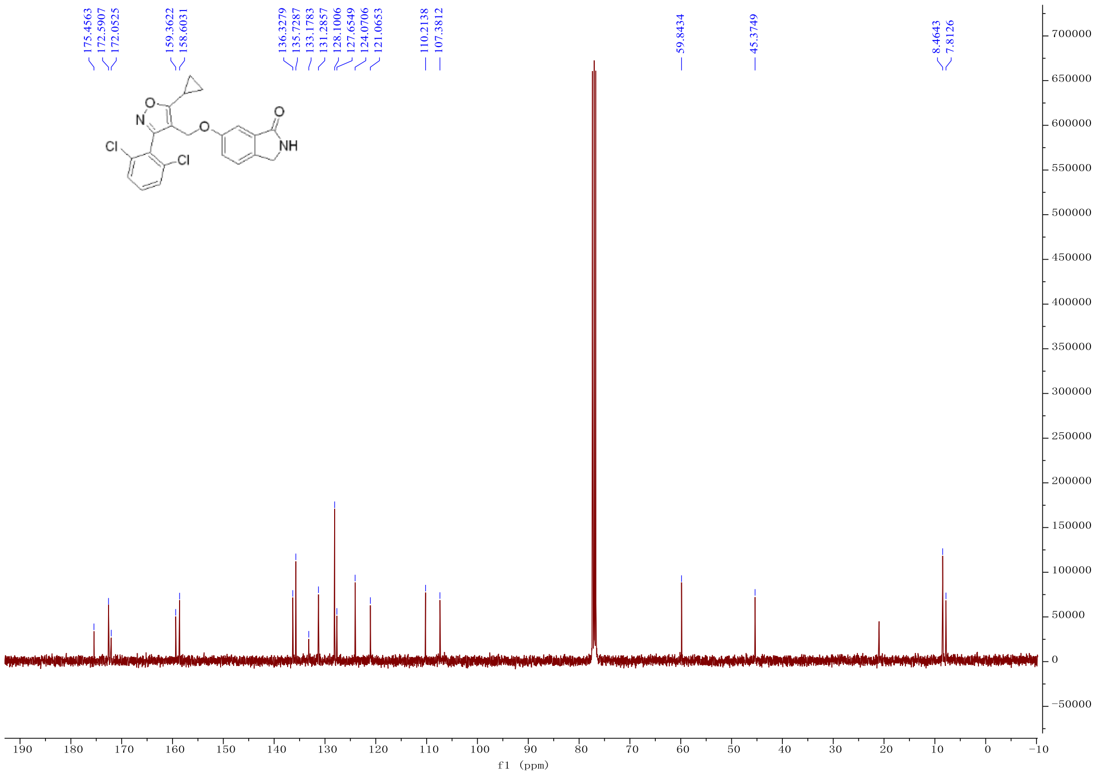

Figure S10: ^{13}C NMR (100 MHz, $CDCl_3$) spectrum of intermediate **6**

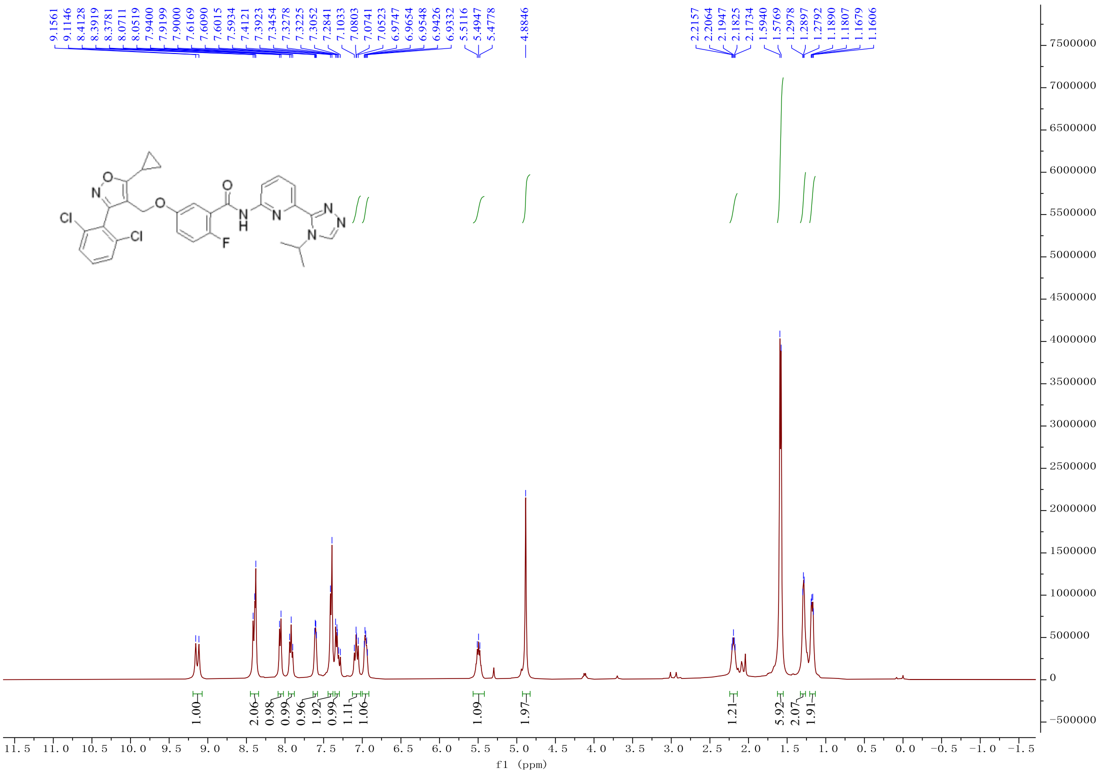

Figure S11: ^{1}H NMR (400 MHz, $CDCl_3$) spectrum of **Z-1**

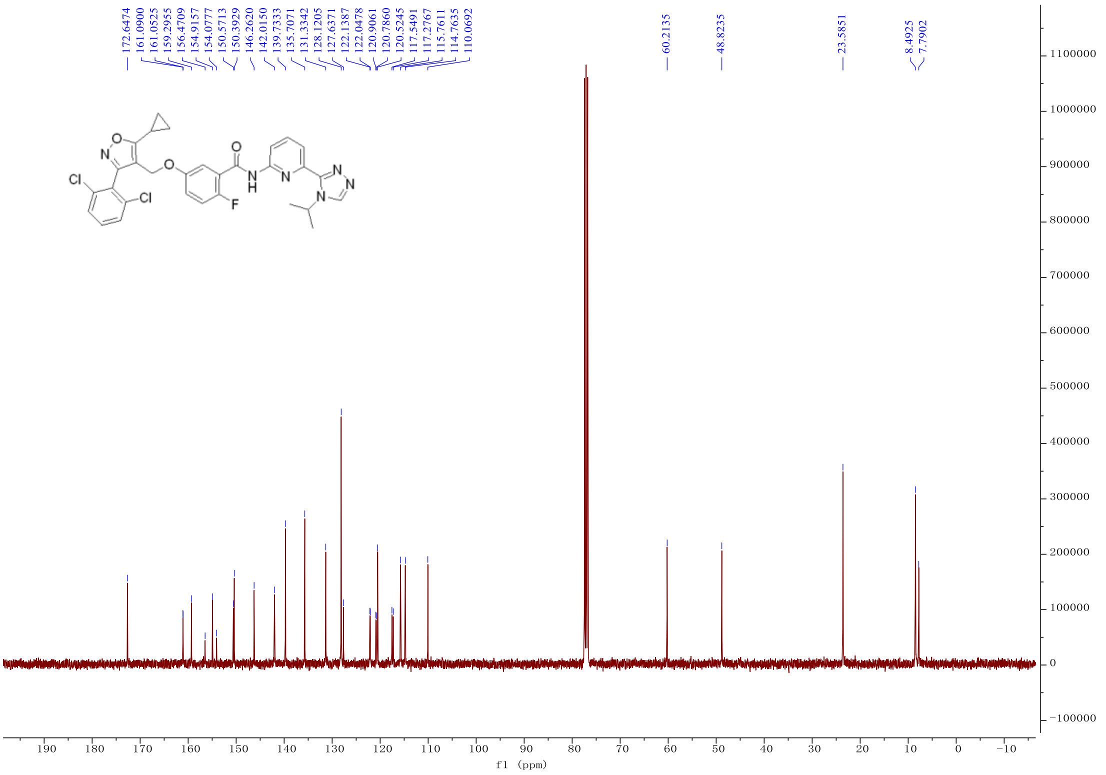

Figure S12: ^{13}C NMR (100 MHz, $CDCl_3$) spectrum of **Z-1**

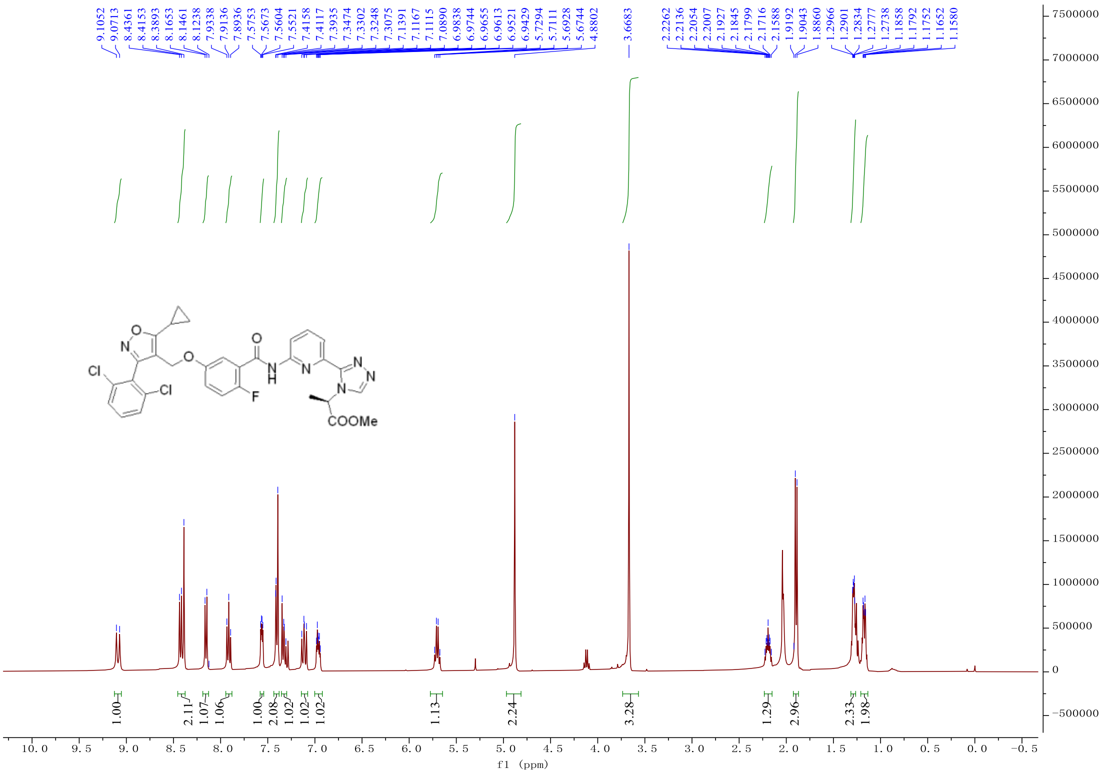

Figure S13: ^{1}H NMR (400 MHz, $CDCl_3$) spectrum of **Z-2**

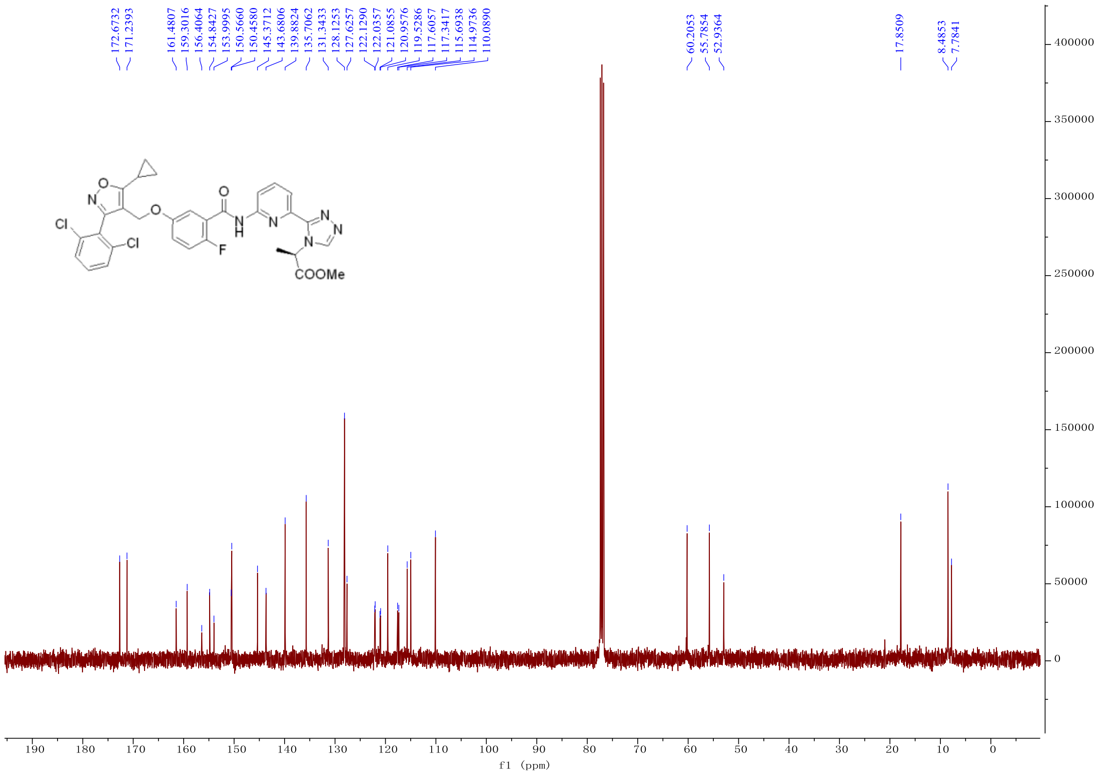

Figure S14: ^{13}C NMR (100 MHz, $CDCl_3$) spectrum of **Z-2**

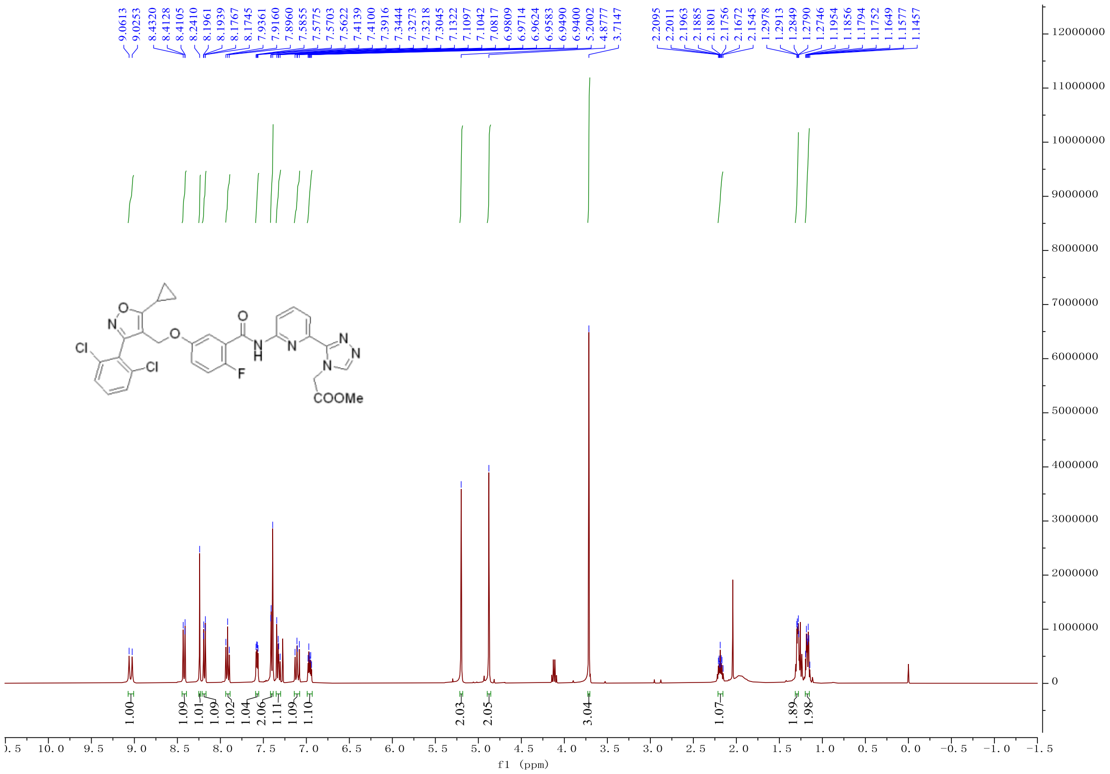

Figure S15: ^{1}H NMR (400 MHz, $CDCl_3$) spectrum of **Z-3**

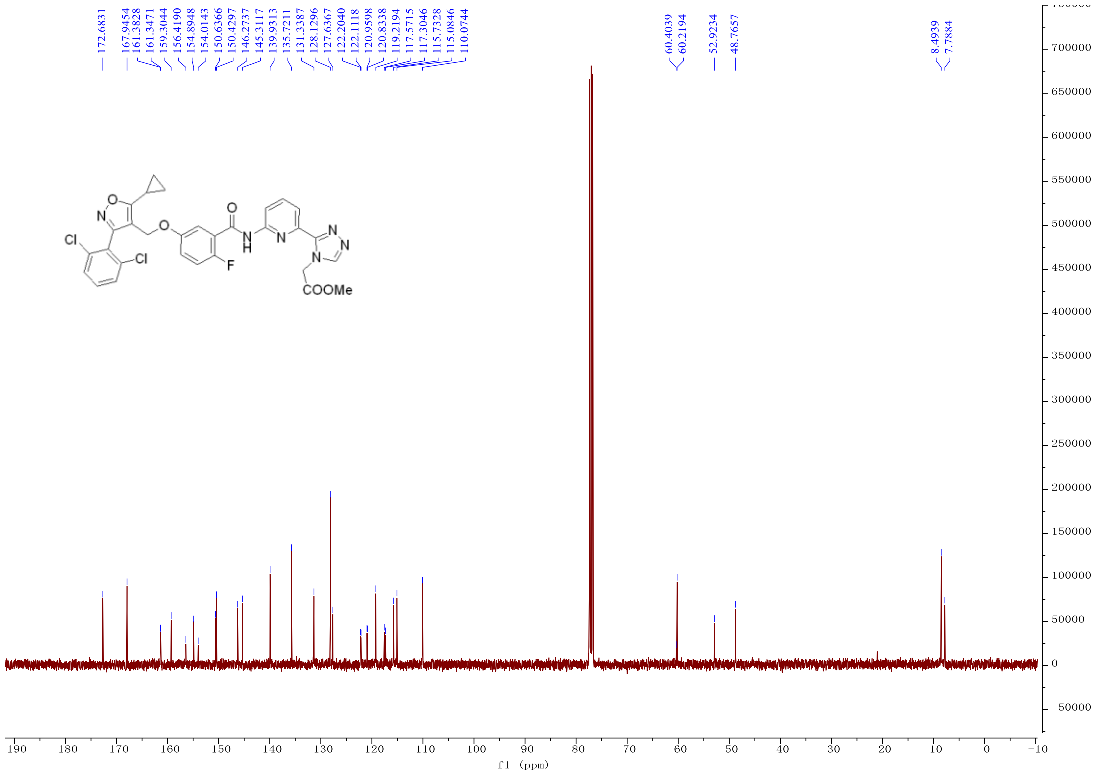

Figure S16: ^{13}C NMR (100 MHz, $CDCl_3$) spectrum of **Z-3**

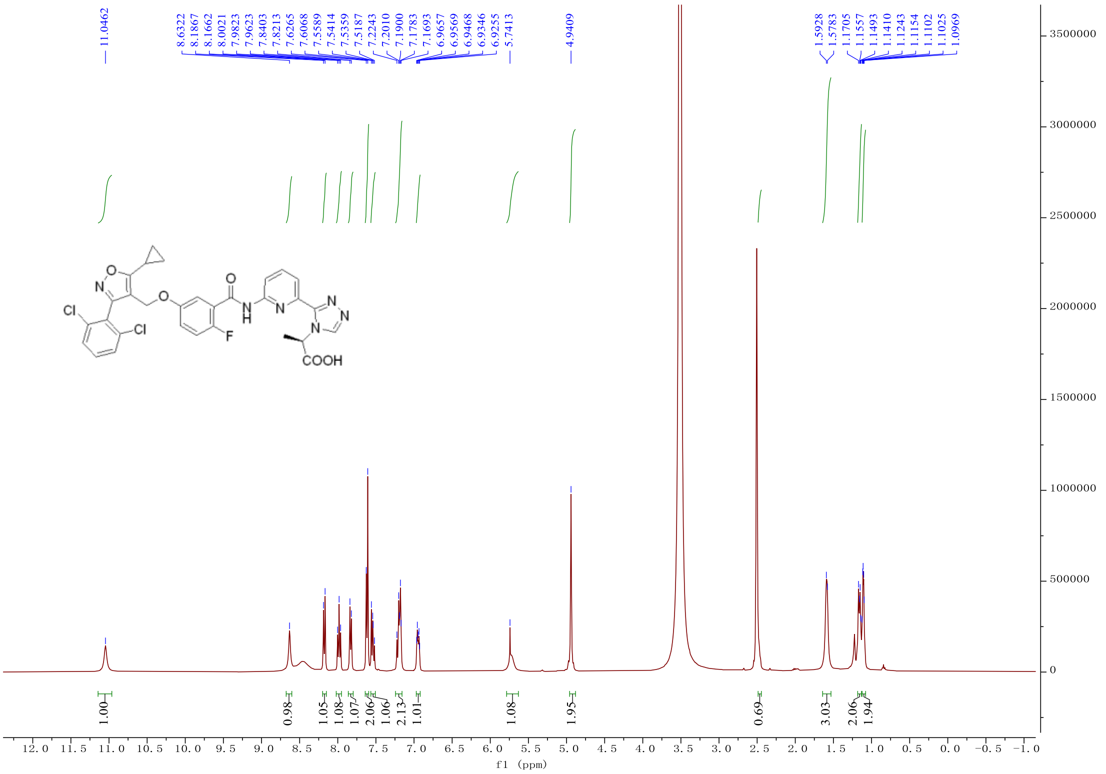

Figure S17: ^{1}H NMR (400 MHz, $CDCl_3$) spectrum of **Z-4**

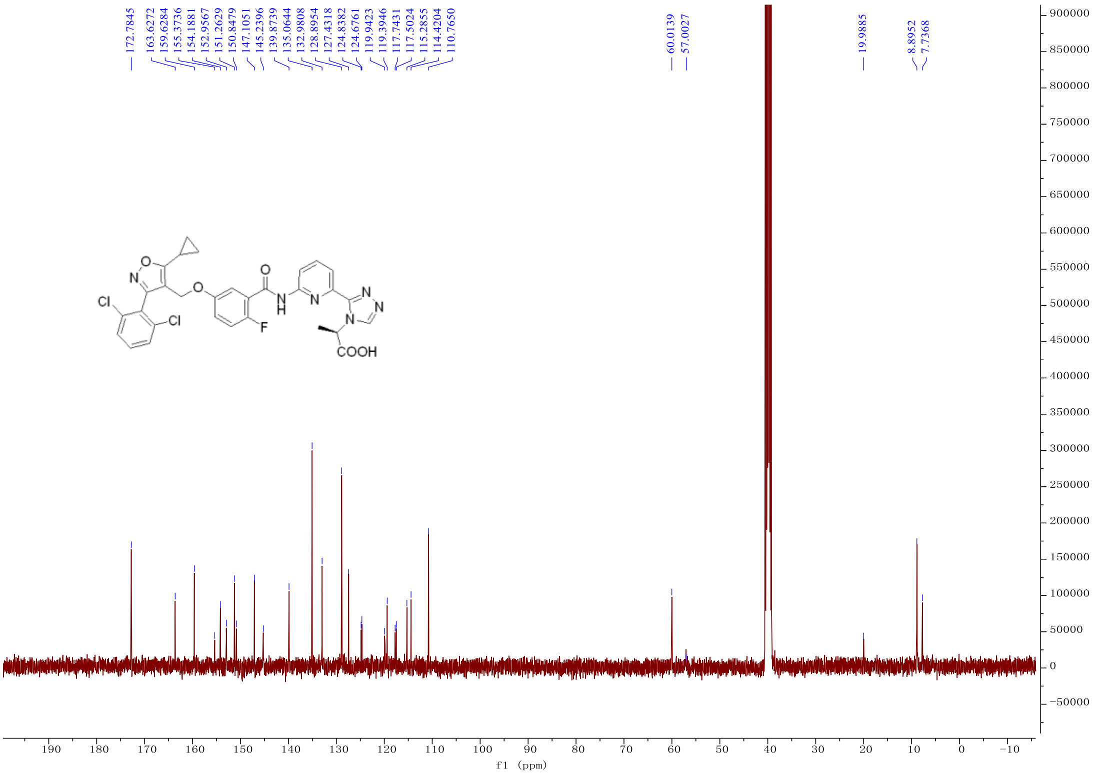

Figure S18: ^{13}C NMR (100 MHz, $CDCl_3$) spectrum of **Z-4**

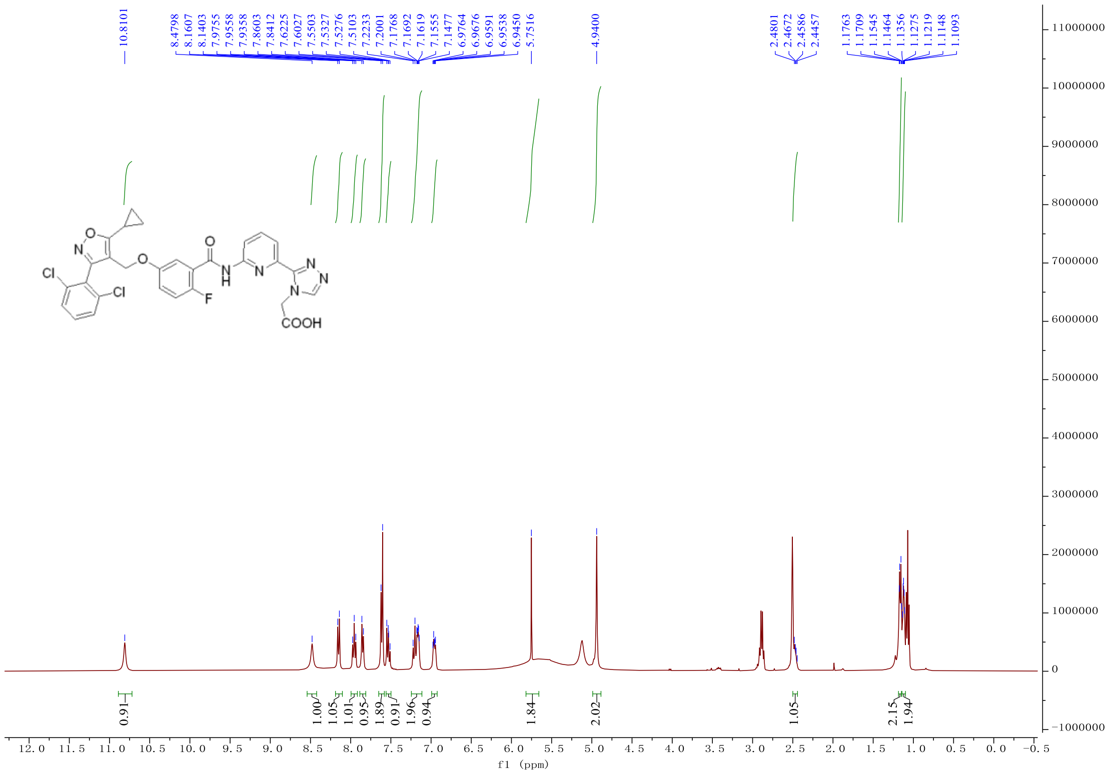

Figure S19: ^{1}H NMR (400 MHz, $CDCl_3$) spectrum of **Z-5**

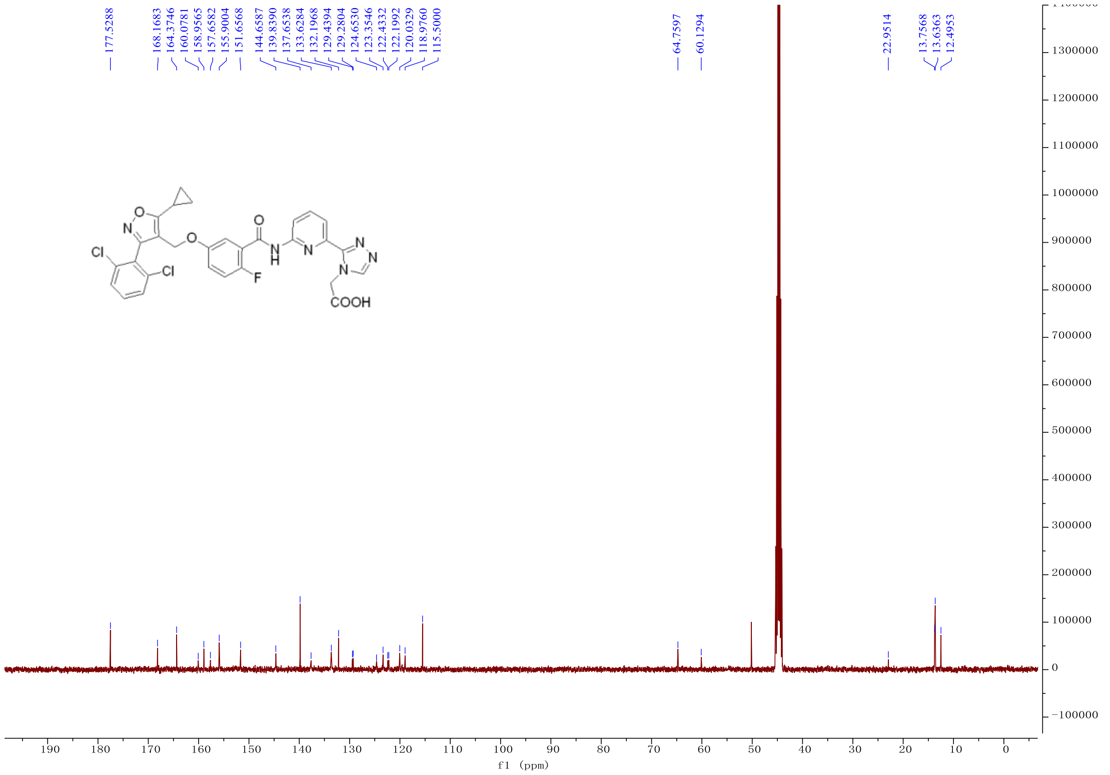

Figure S20: ^{13}C NMR (100 MHz, $CDCl_3$) spectrum of **Z-5**

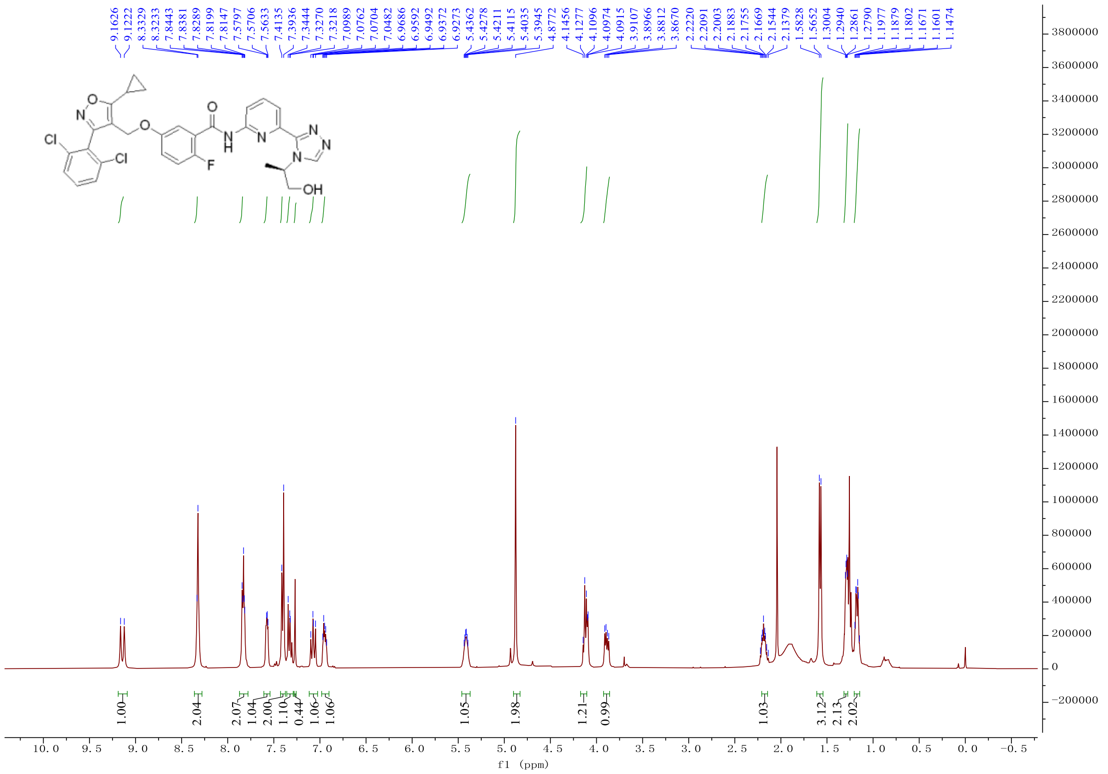

Figure S21: ^{1}H NMR (400 MHz, $CDCl_3$) spectrum of **Z-6**

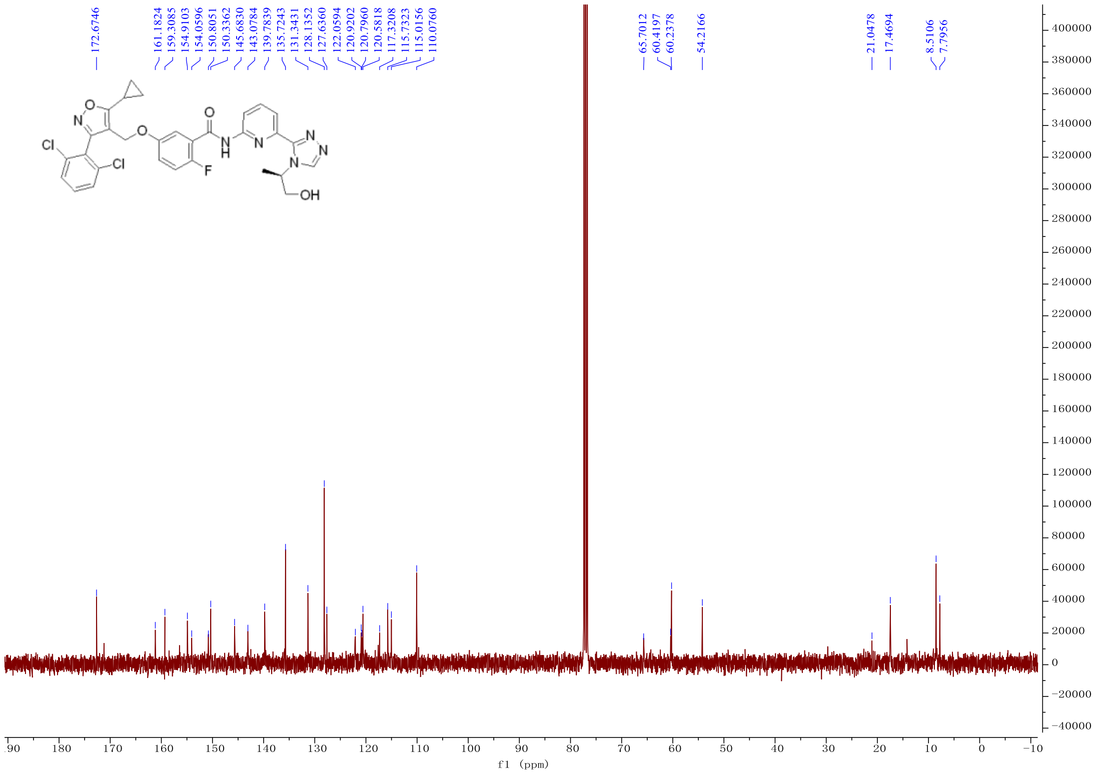

Figure S22: ^{13}C NMR (100 MHz, $CDCl_3$) spectrum of **Z-6**

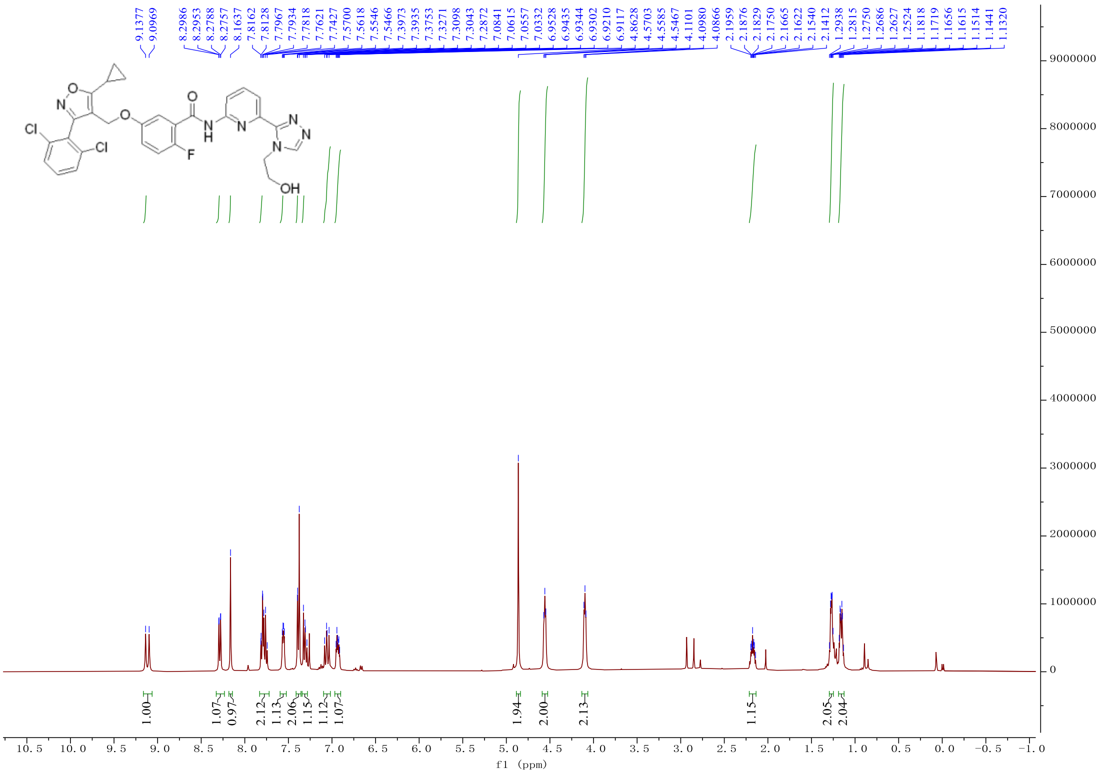

Figure S23: ^{1}H NMR (400 MHz, $CDCl_3$) spectrum of **Z-7**

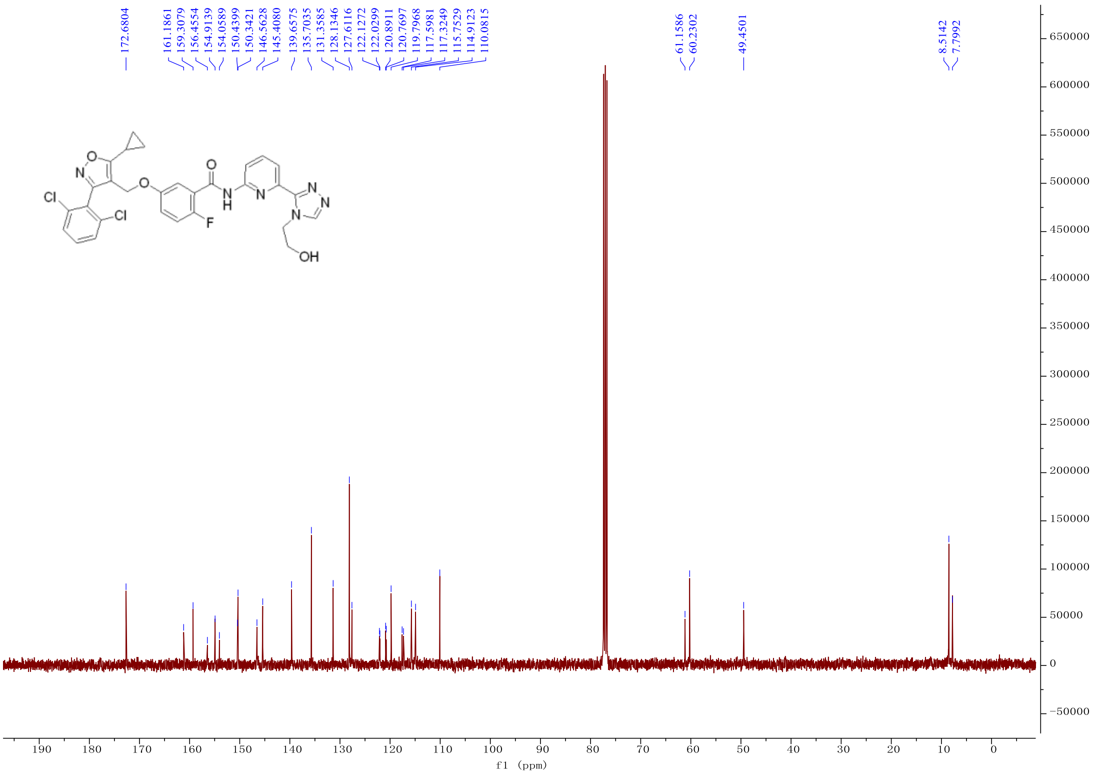

Figure S24: ^{13}C NMR (100 MHz, $CDCl_3$) spectrum of **Z-7**

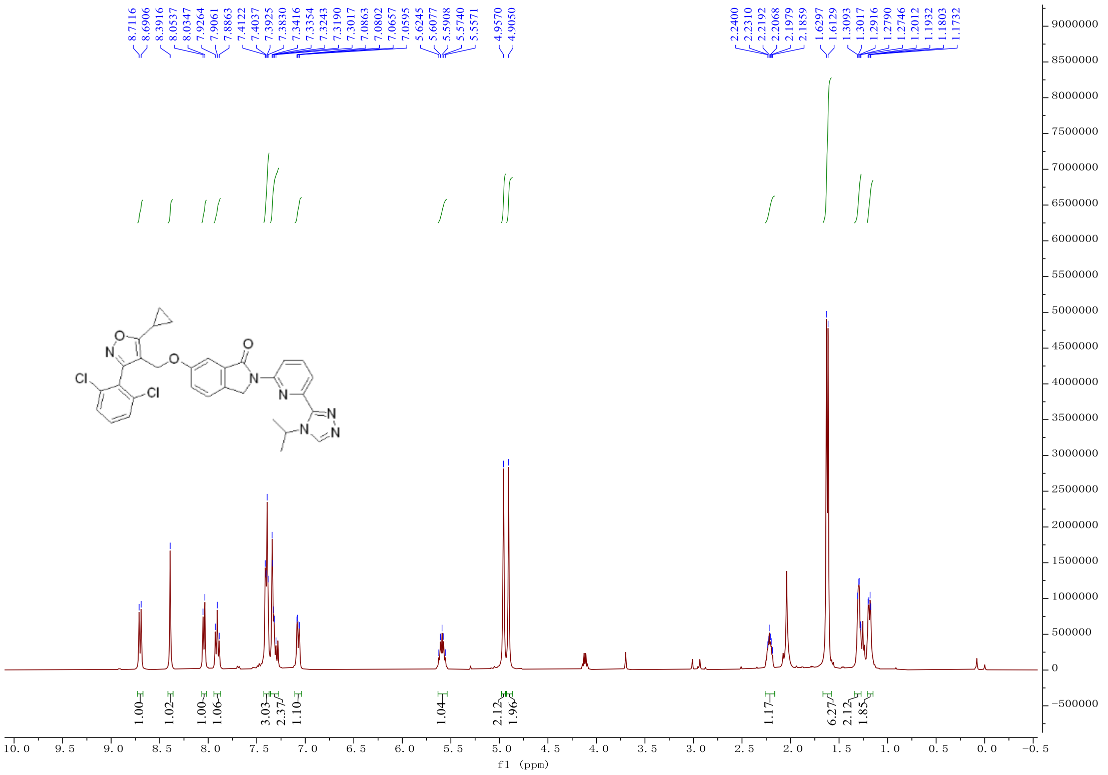

Figure S25: ^{1}H NMR (400 MHz, $CDCl_3$) spectrum of **Z-8**

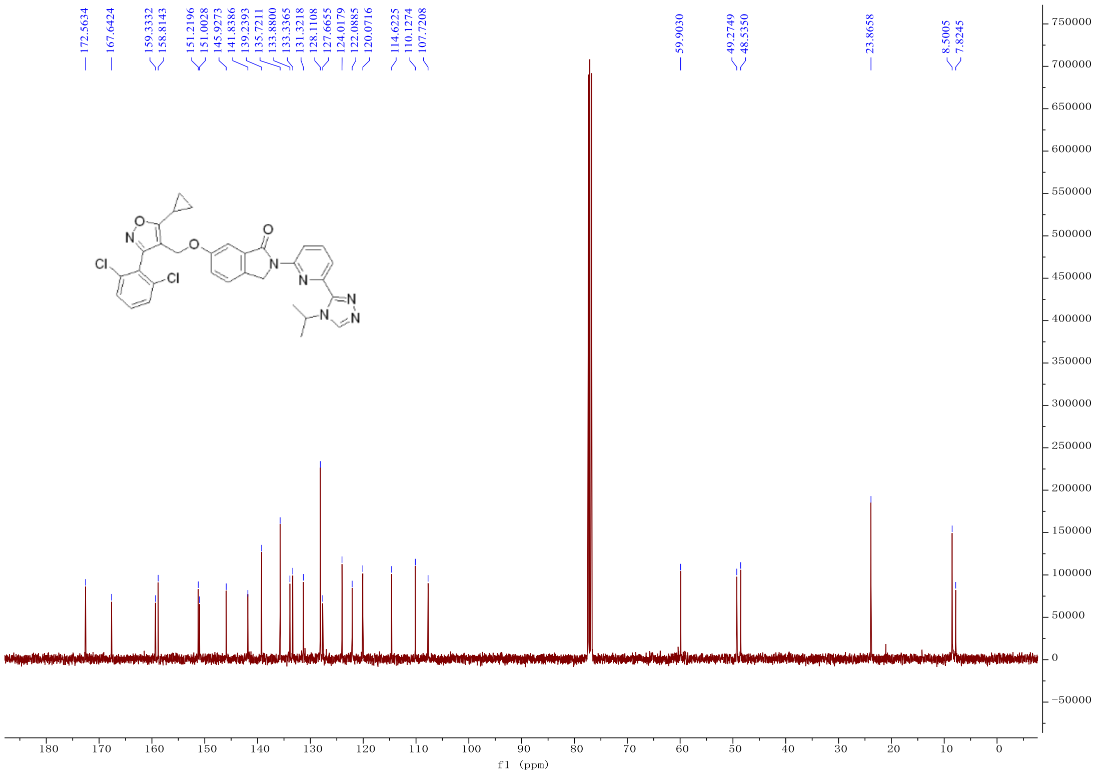

Figure S26: ^{13}C NMR (100 MHz, $CDCl_3$) spectrum of **Z-8**

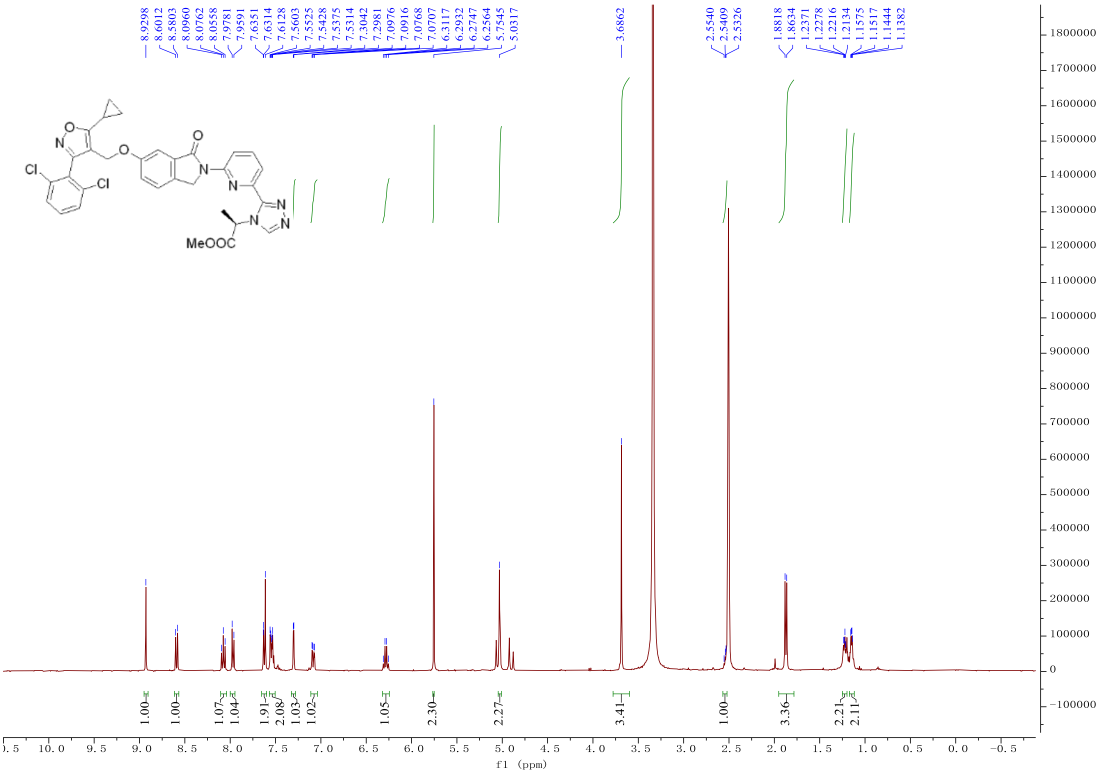

Figure S27: ^{1}H NMR (400 MHz, $CDCl_3$) spectrum of **Z-9**

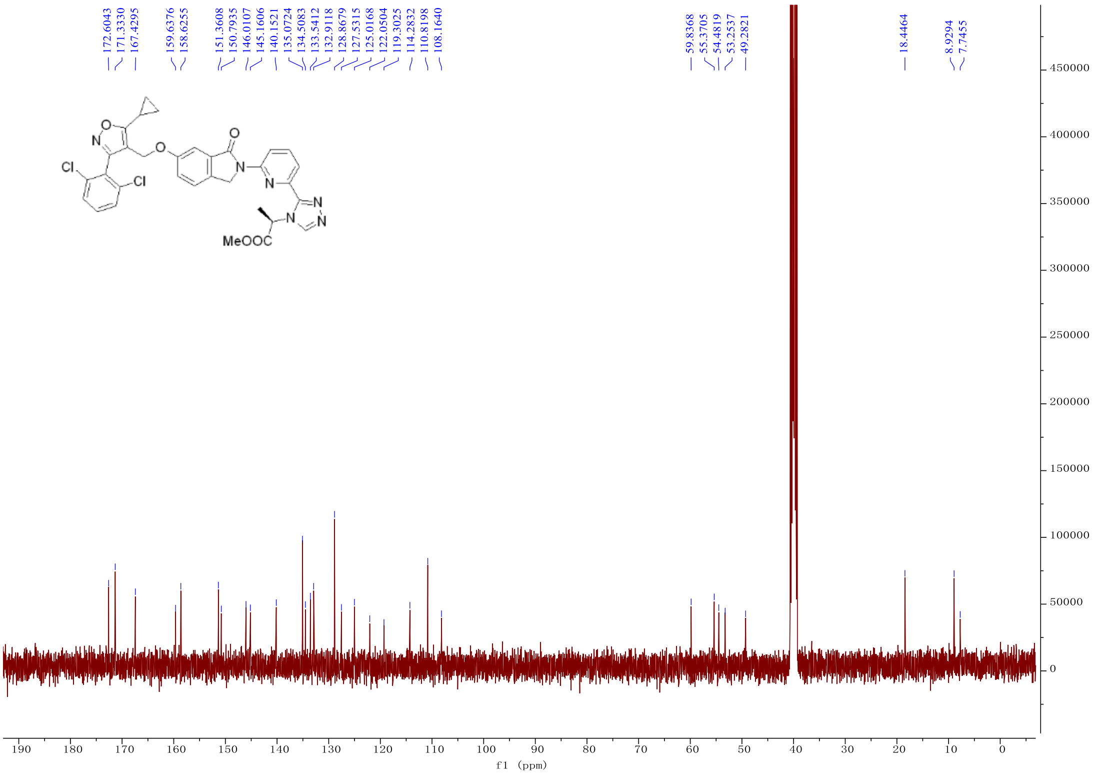

Figure S28: ^{13}C NMR (100 MHz, $CDCl_3$) spectrum of **Z-9**

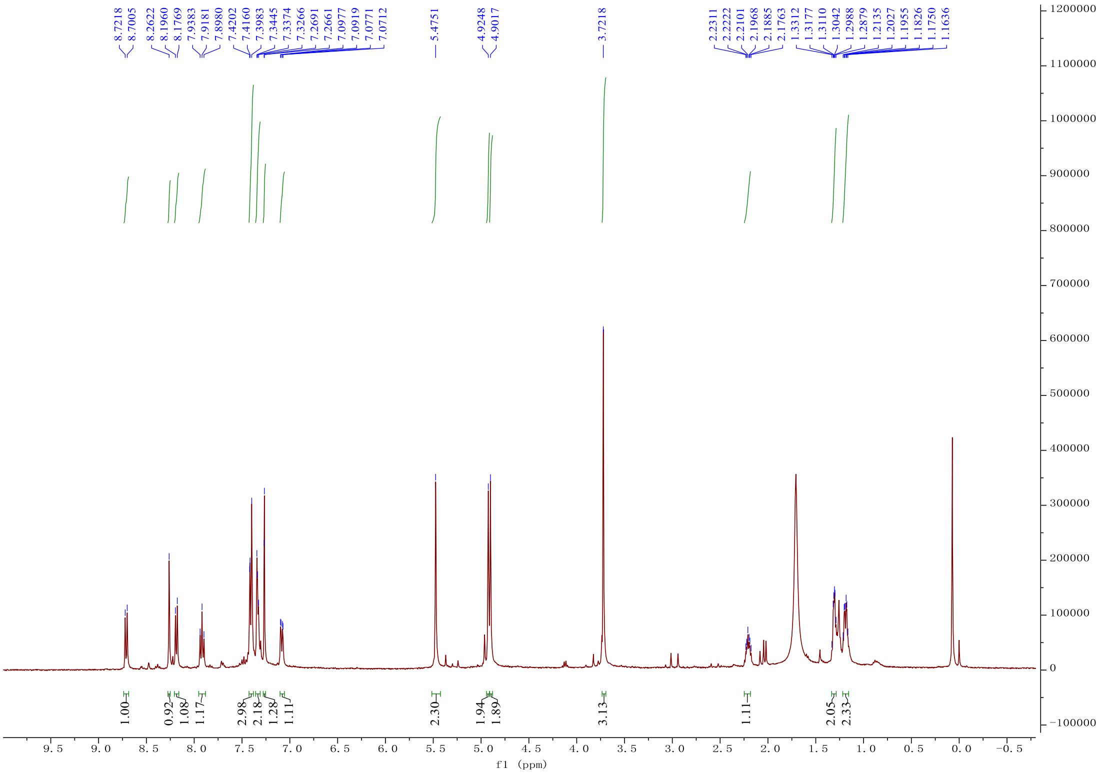

Figure S29: ^{1}H NMR (400 MHz, CDCl3) spectrum of **Z-10**

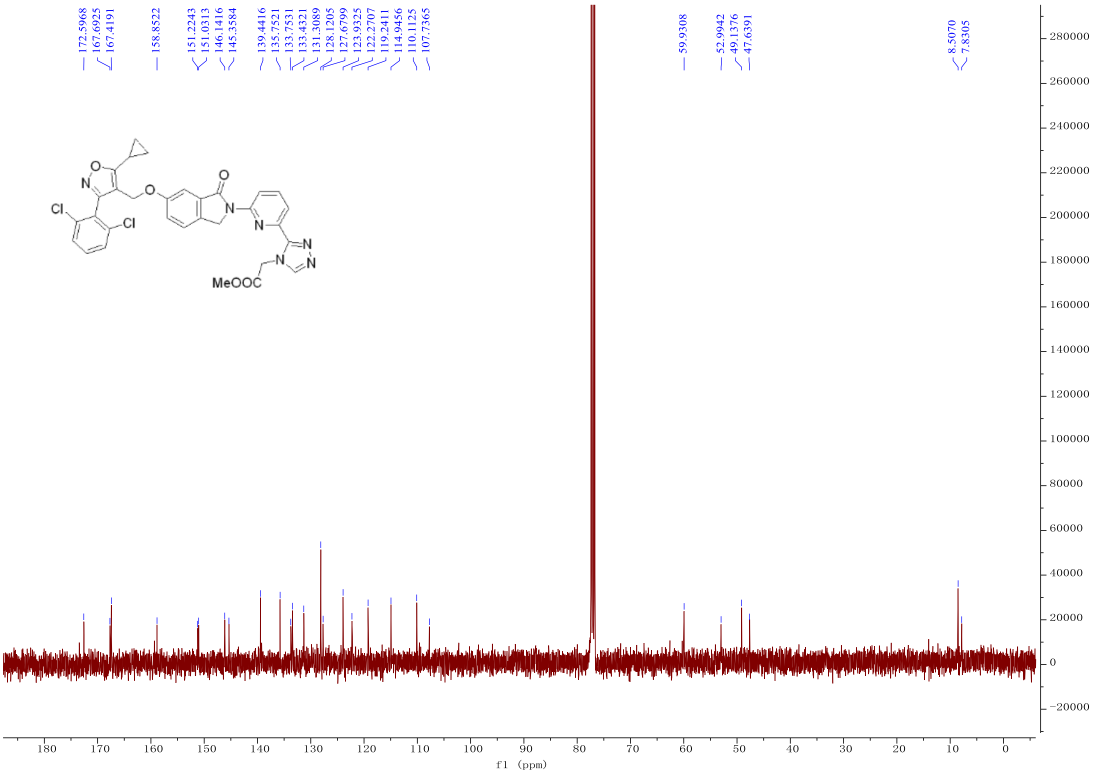

Figure S30: ^{13}C NMR (100 MHz, CDCl3) spectrum of **Z-10**

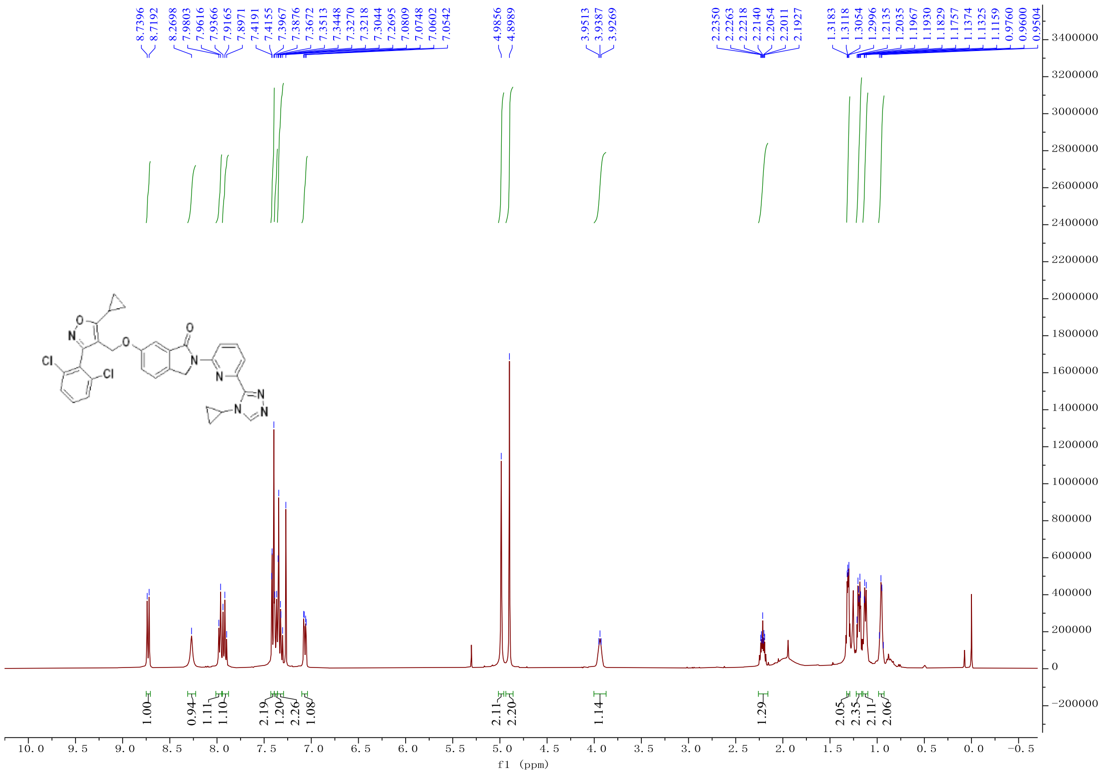

Figure S31: ^{1}H NMR (400 MHz, $CDCl_3$) spectrum of **Z-11**

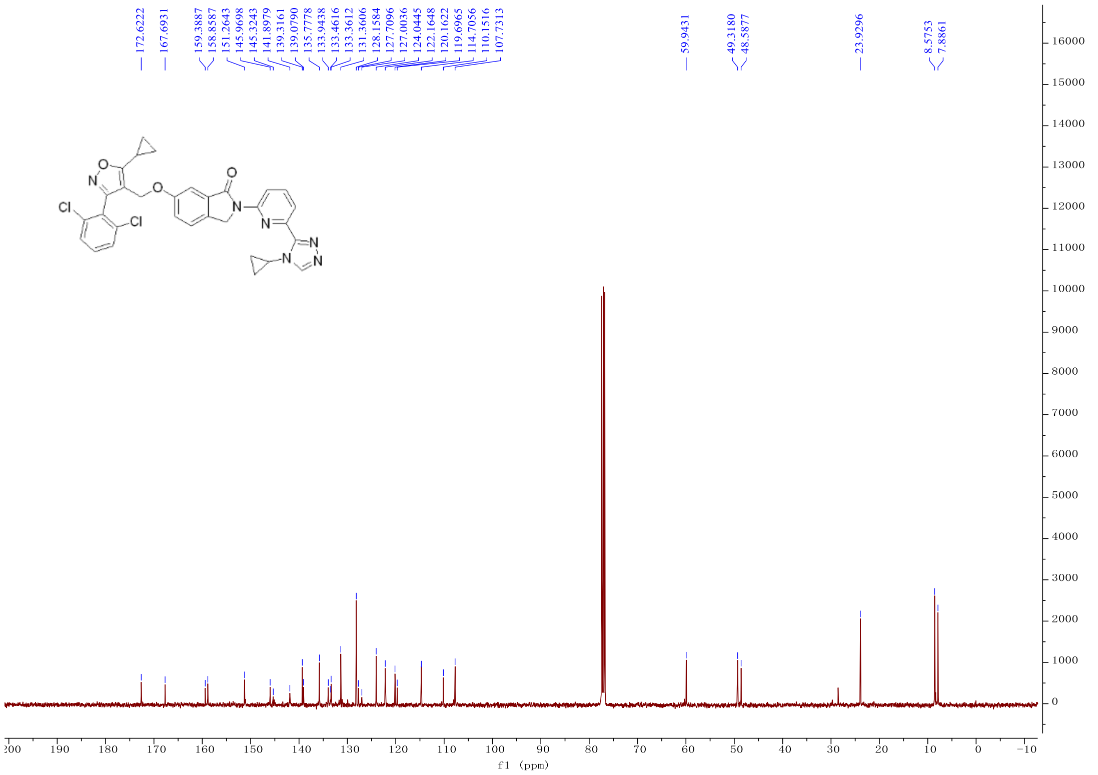

Figure S32: ^{13}C NMR (100 MHz, $CDCl_3$) spectrum of **Z-11**

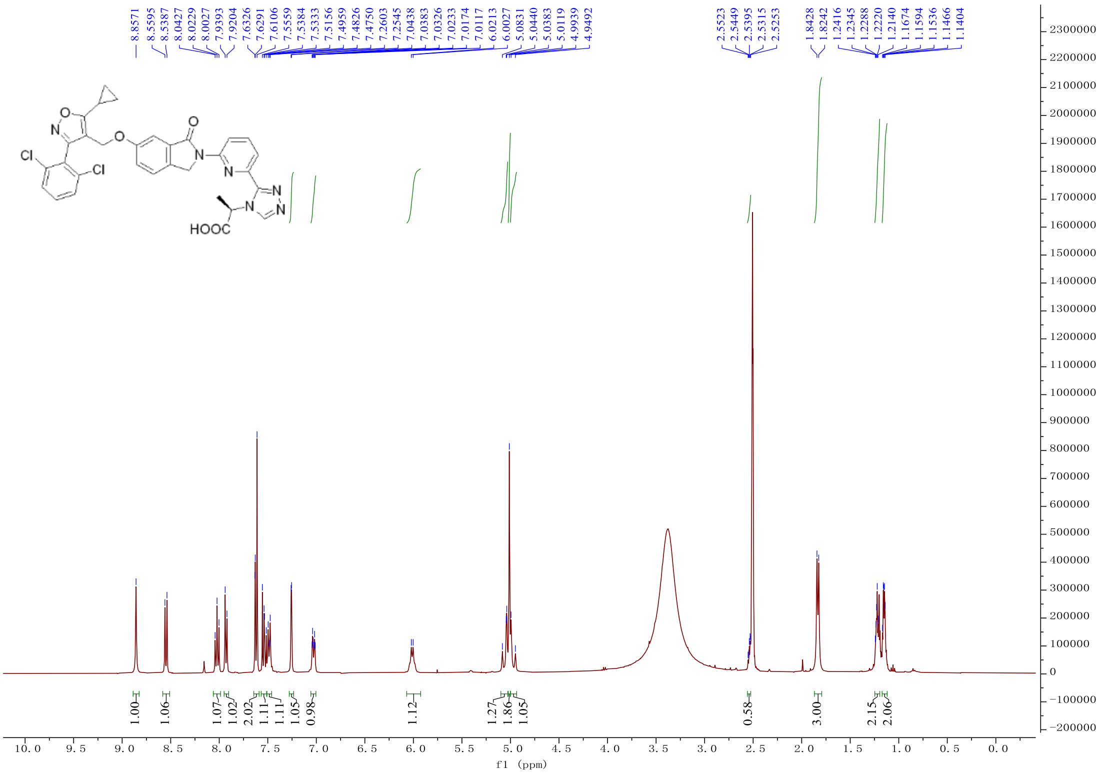

Figure S33: ^{1}H NMR (400 MHz, CDCl3) spectrum of **Z-12**

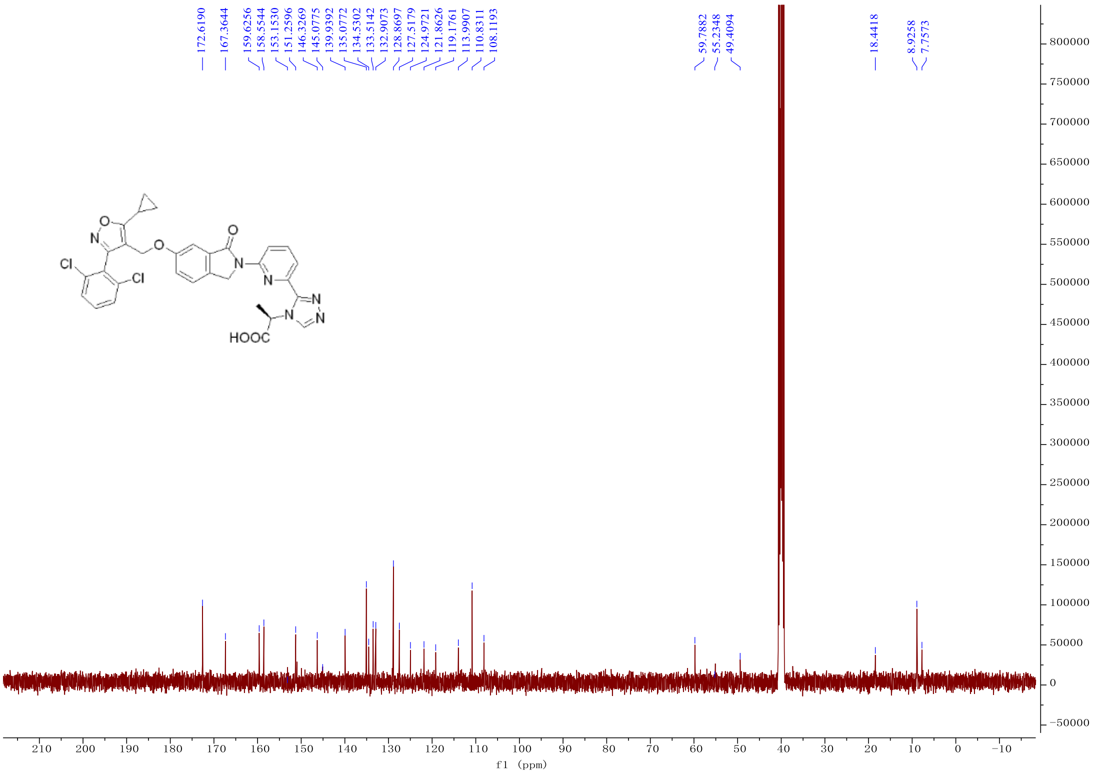

Figure S34: ^{13}C NMR (100 MHz, CDCl3) spectrum of **Z-12**

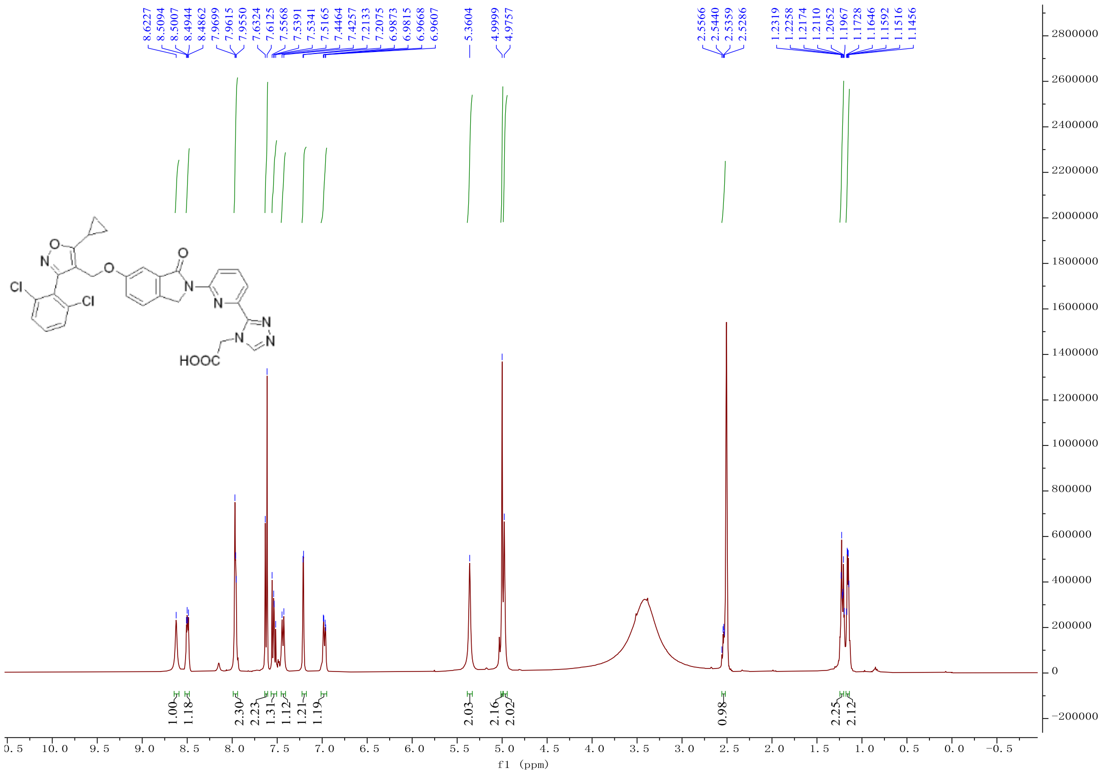

Figure S35: ^{1}H NMR (400 MHz, $CDCl_3$) spectrum of **Z-13**

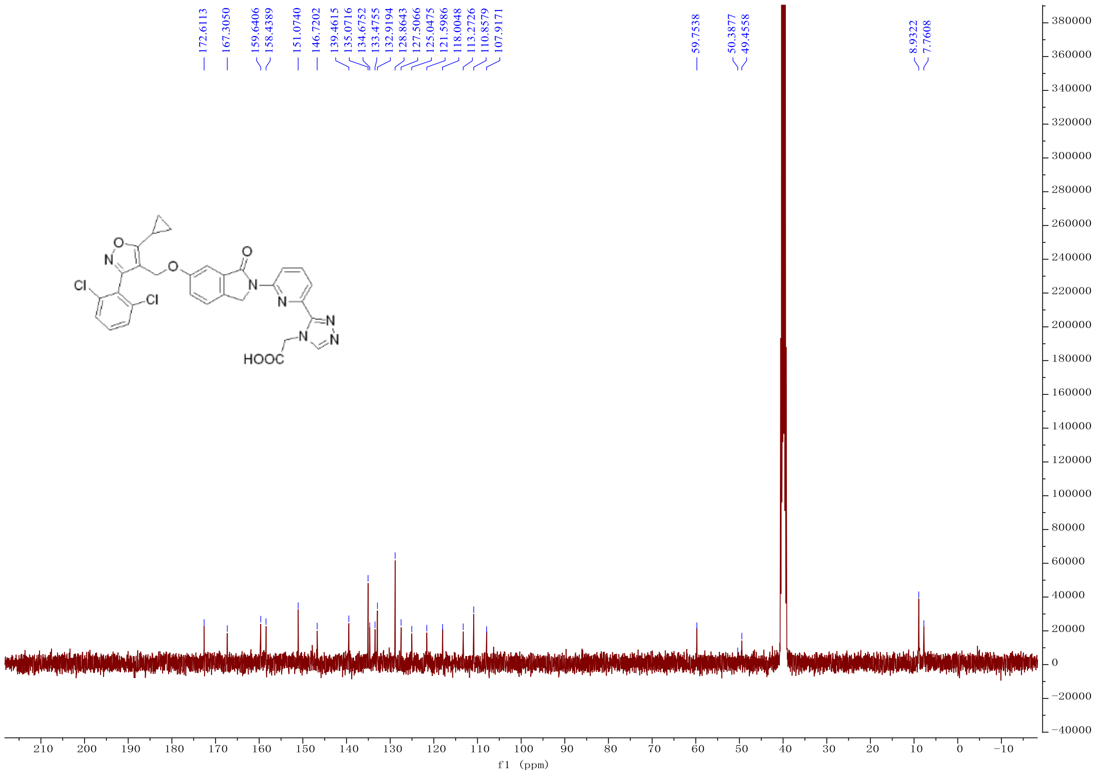

Figure S36: ^{13}C NMR (100 MHz, $CDCl_3$) spectrum of **Z-13**

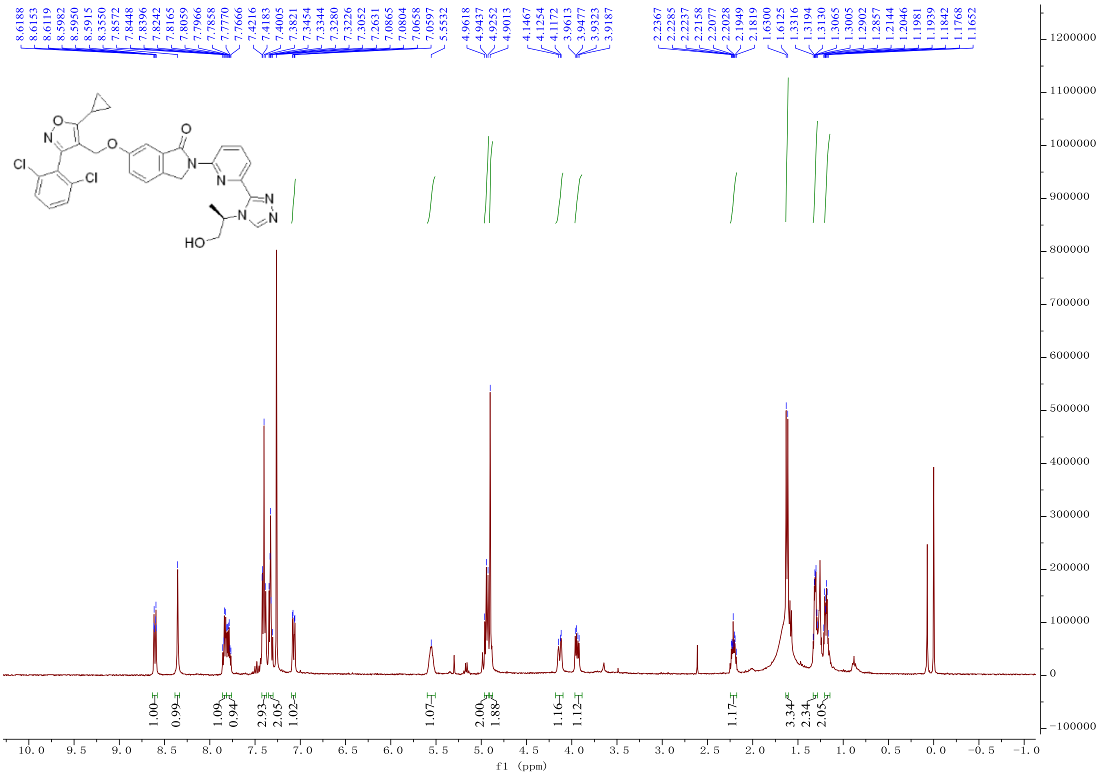

Figure S37: ^{1}H NMR (400 MHz, $CDCl_3$) spectrum of **Z-14**

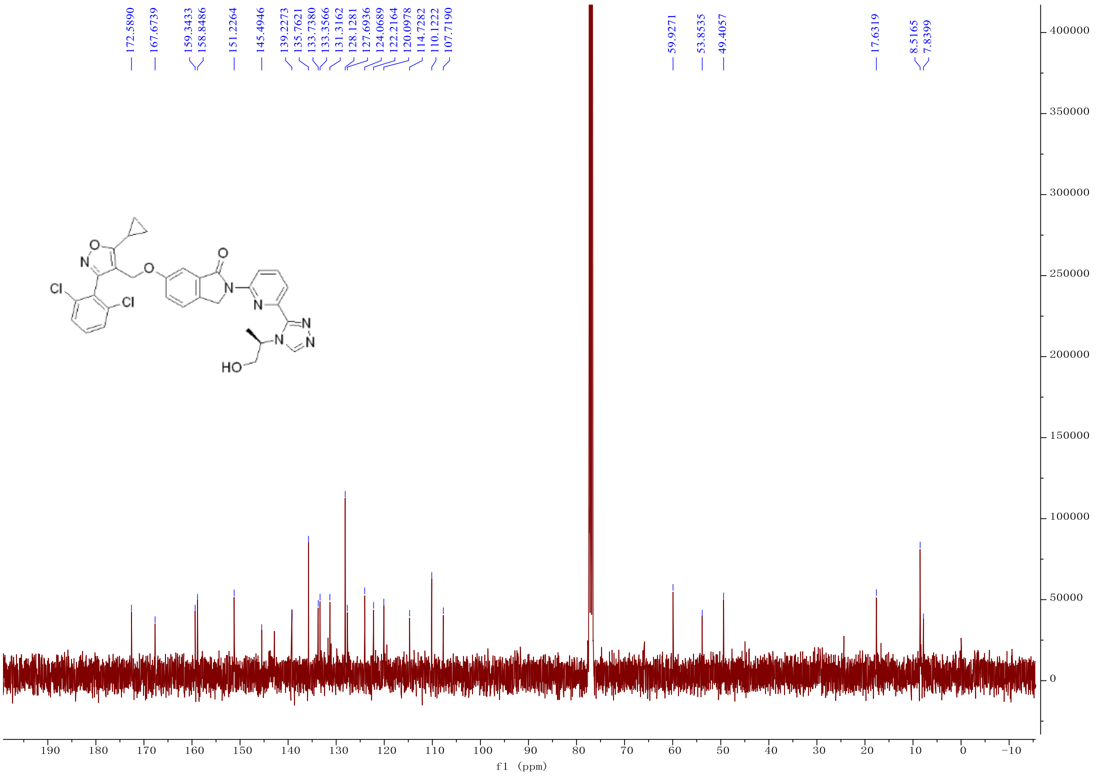

Figure S38: ^{13}C NMR (100 MHz, $CDCl_3$) spectrum of **Z-14**

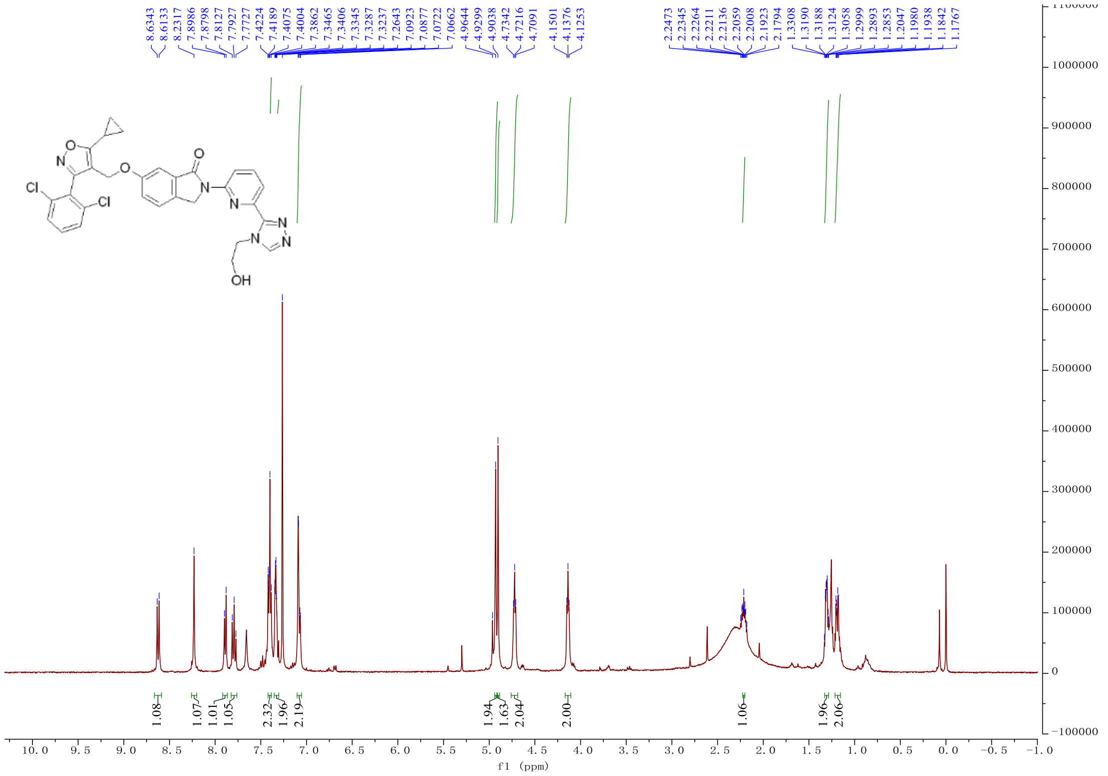

Figure S39: ^{1}H NMR (400 MHz, $CDCl_3$) spectrum of **Z-15**

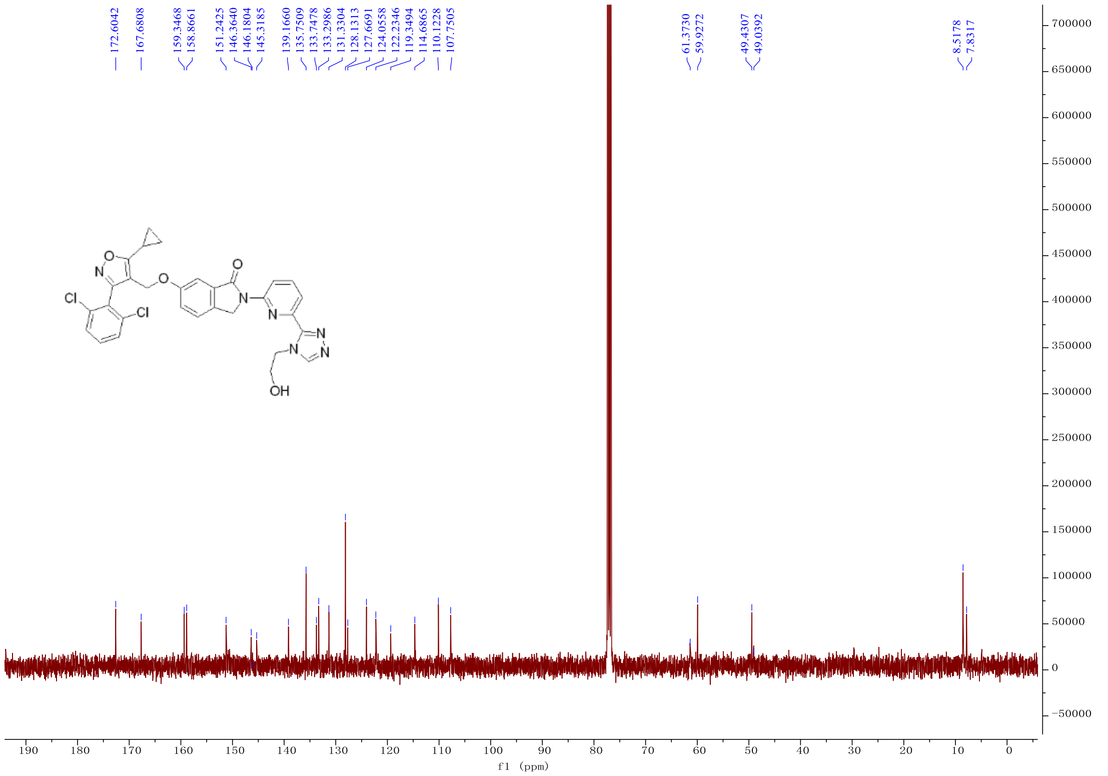

Figure S40: ^{13}C NMR (100 MHz, $CDCl_3$) spectrum of **Z-15**

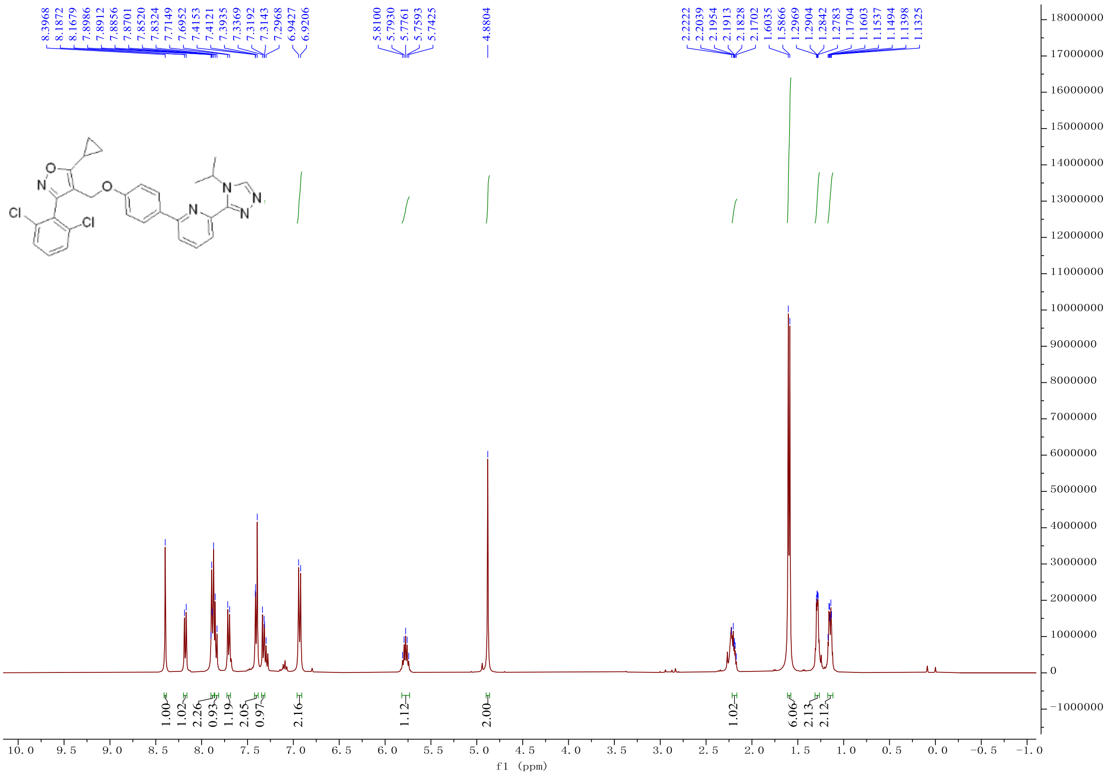

Figure S41: ^{1}H NMR (400 MHz, $CDCl_3$) spectrum of **Z-16**

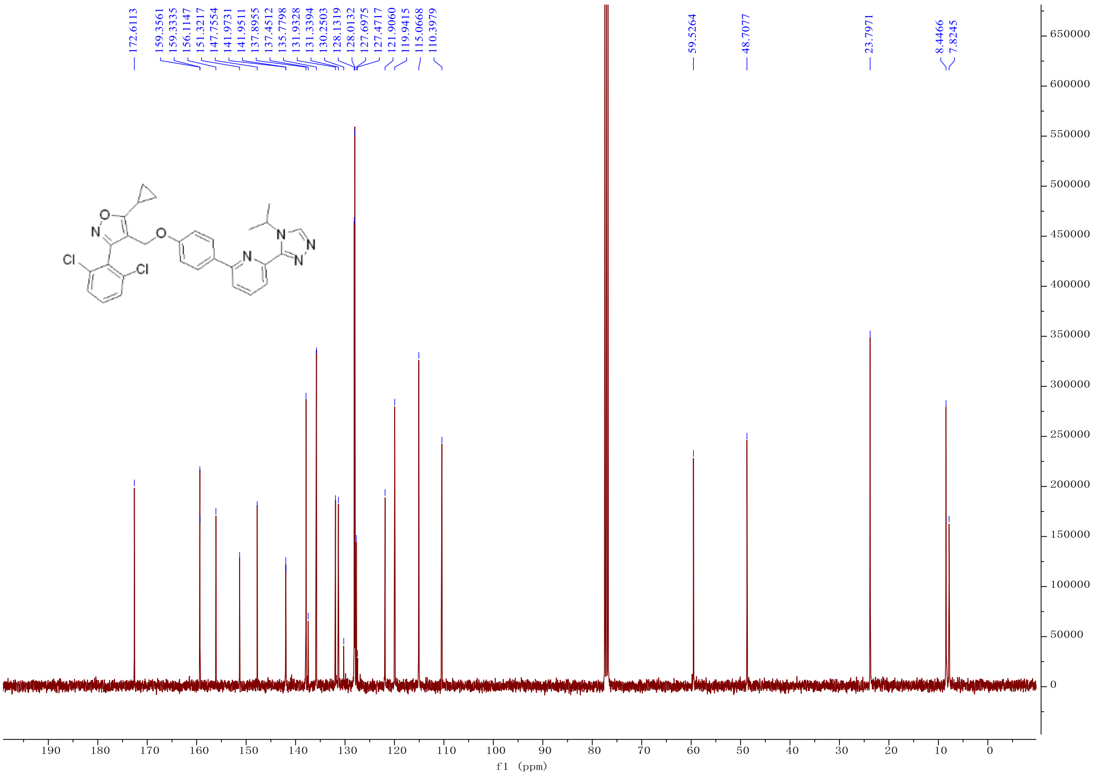

Figure S42: ^{13}C NMR (100 MHz, $CDCl_3$) spectrum of **Z-16**

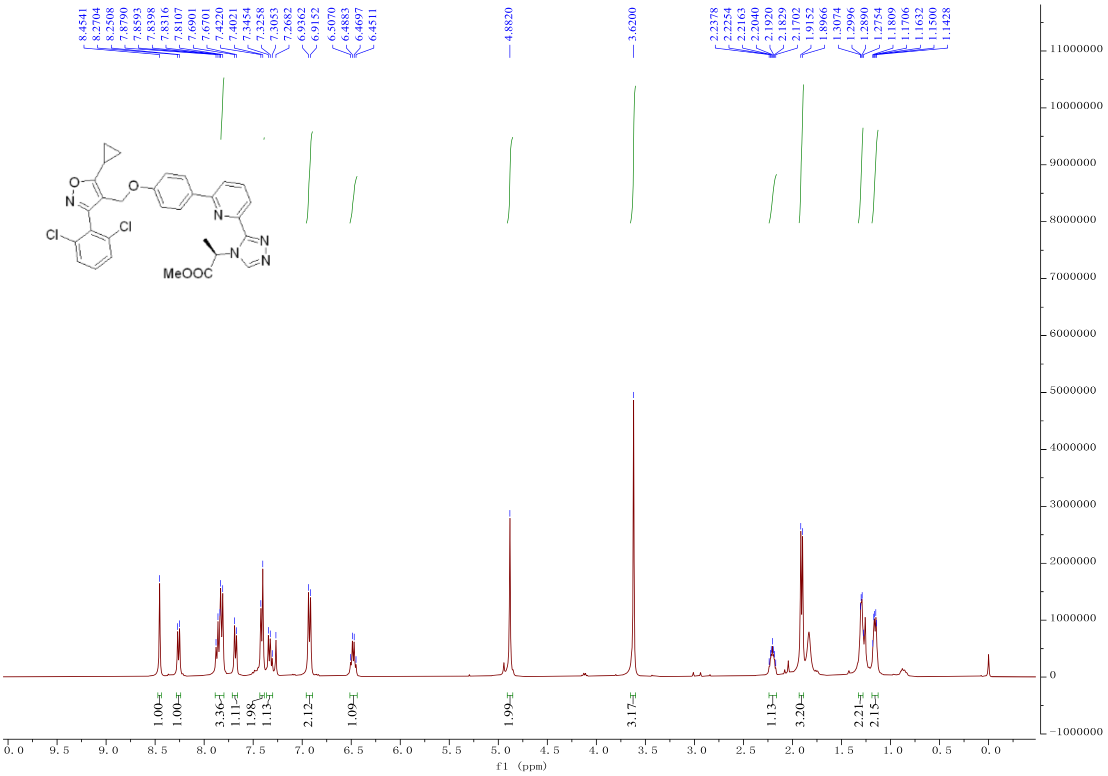

Figure S43: ^{1}H NMR (400 MHz, $CDCl_3$) spectrum of **Z-17**

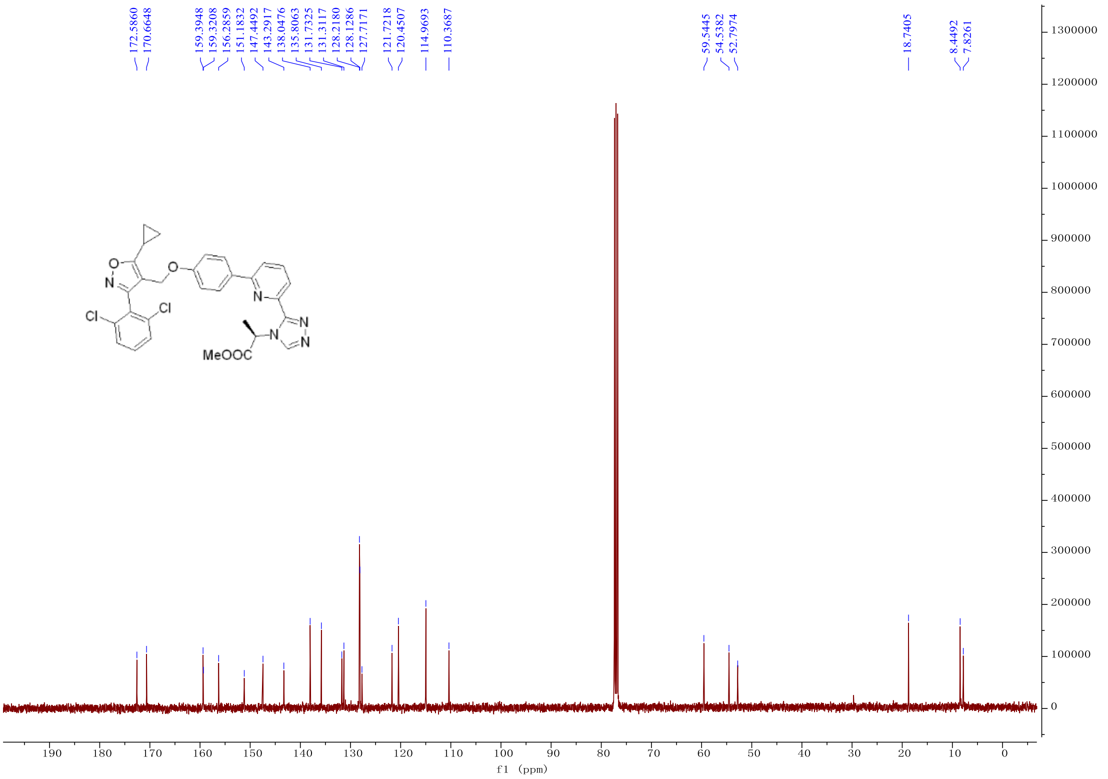

Figure S44: ^{13}C NMR (100 MHz, $CDCl_3$) spectrum of **Z-17**

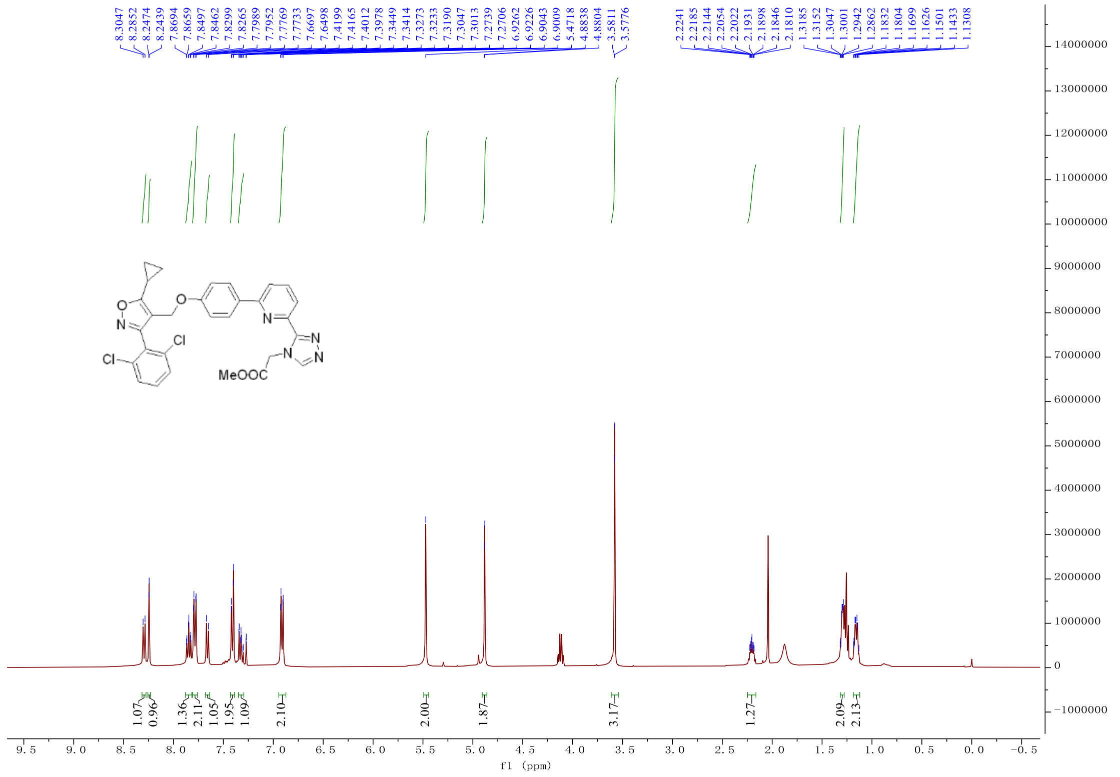

Figure S45: ^{1}H NMR (400 MHz, $CDCl_3$) spectrum of **Z-18**

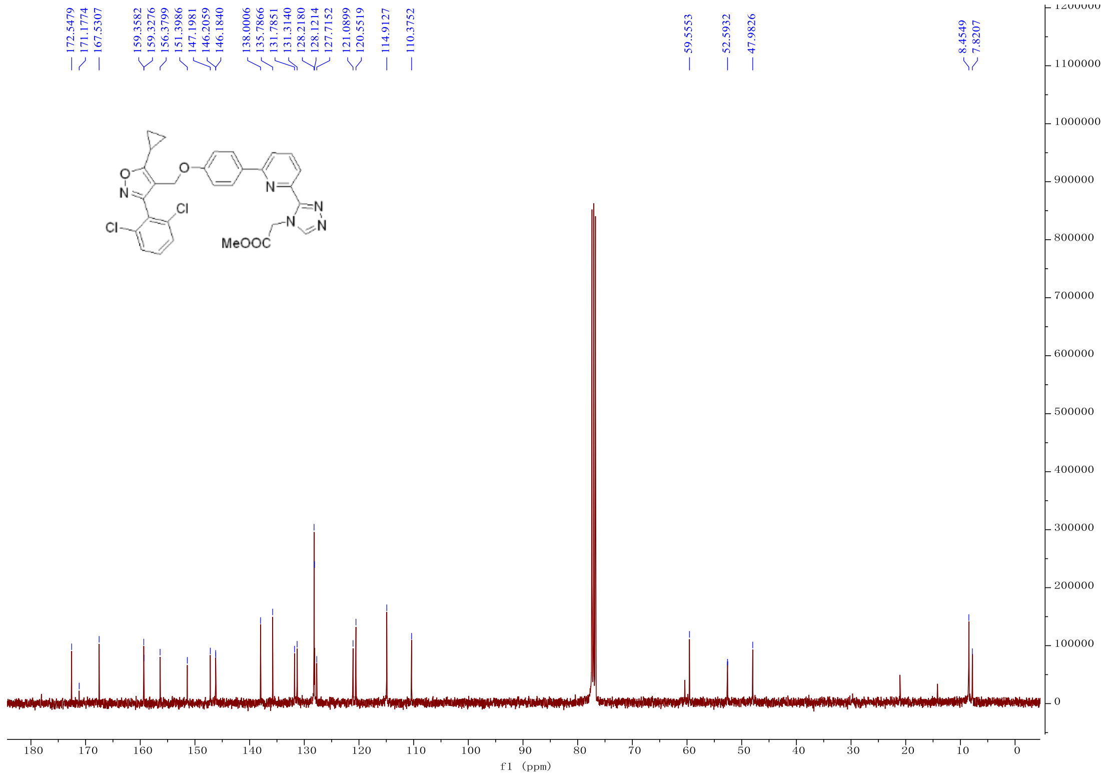

Figure S46: ^{13}C NMR (100 MHz, $CDCl_3$) spectrum of **Z-18**

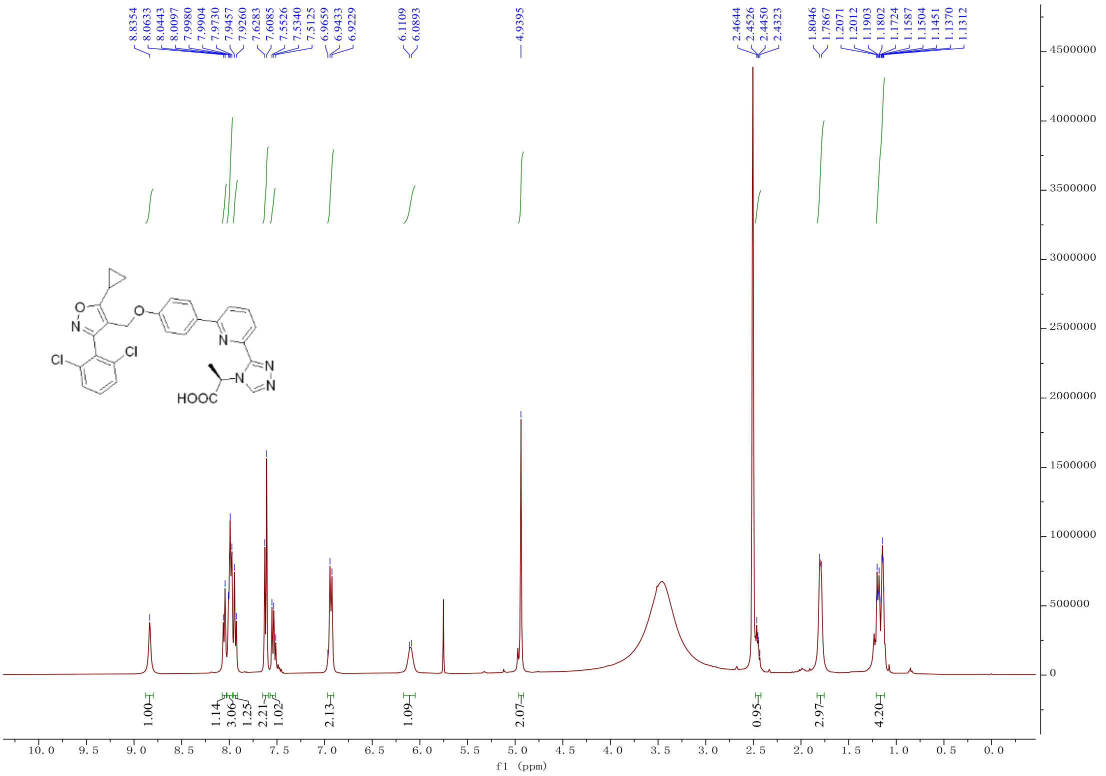

Figure S47: ^{1}H NMR (400 MHz, $CDCl_3$) spectrum of **Z-19**

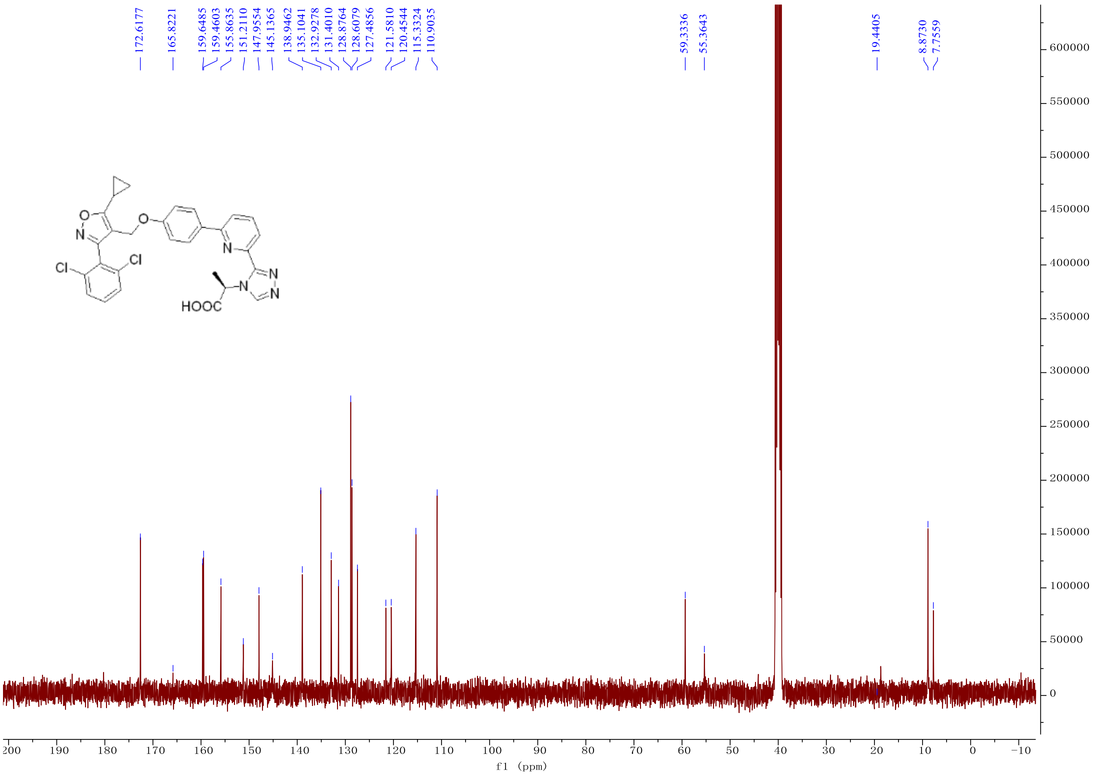

Figure S48: ^{13}C NMR (100 MHz, $CDCl_3$) spectrum of **Z-19**

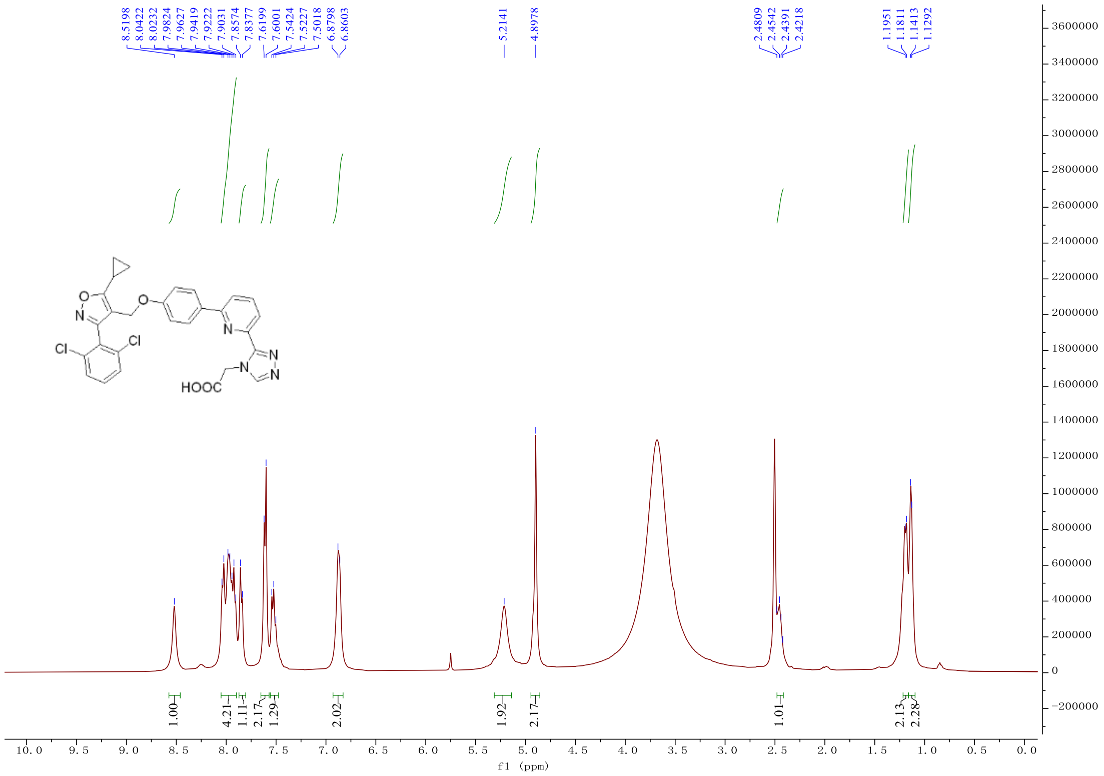

Figure S49: ^{1}H NMR (400 MHz, $CDCl_3$) spectrum of **Z-20**

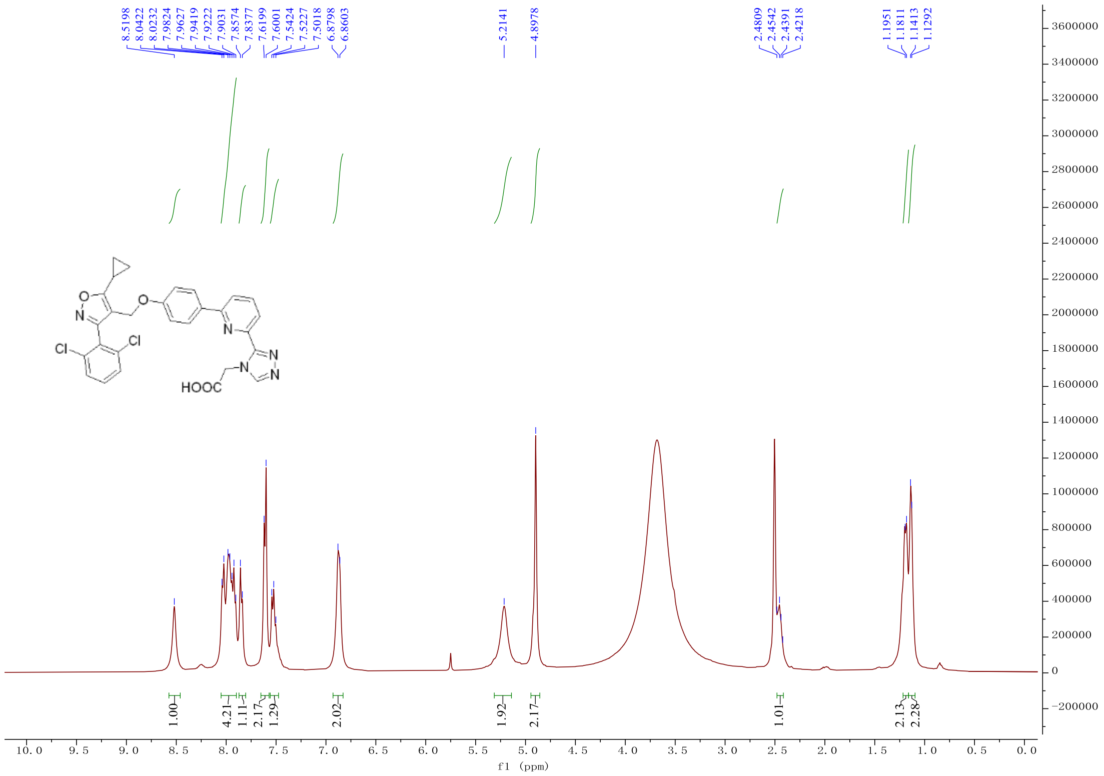

Figure S50: ^{13}C NMR (100 MHz, $CDCl_3$) spectrum of **Z-20**

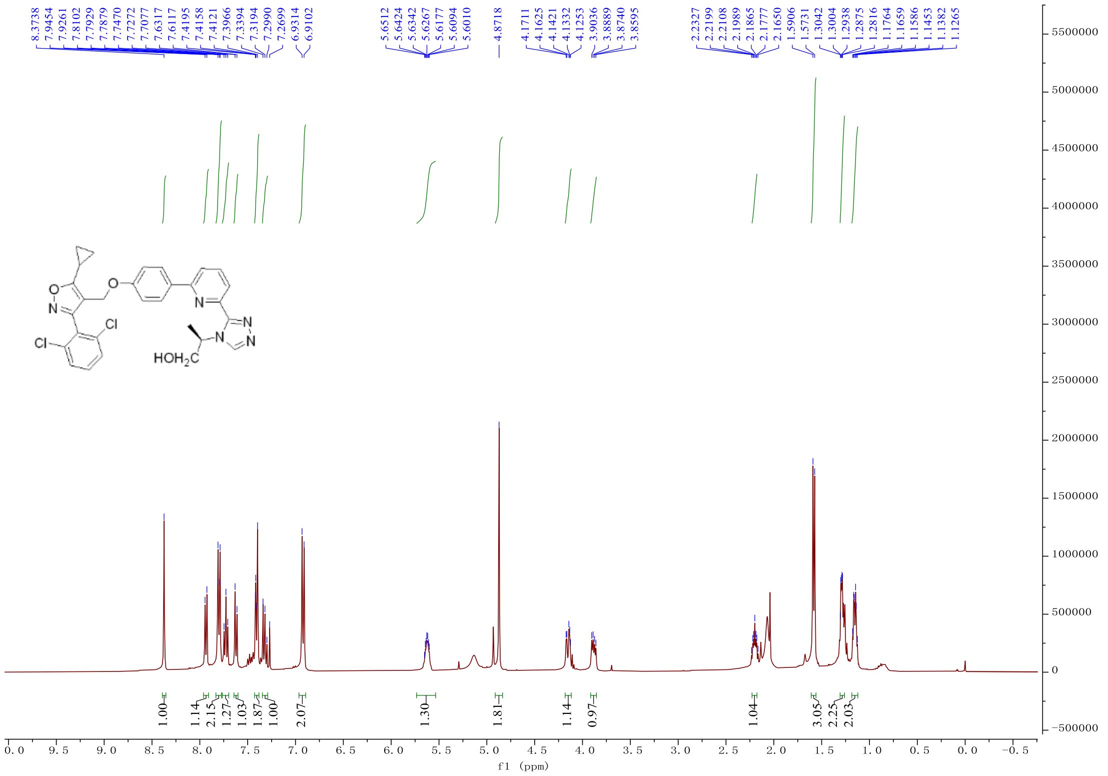

Figure S51: ^{1}H NMR (400 MHz, $CDCl_3$) spectrum of **Z-21**

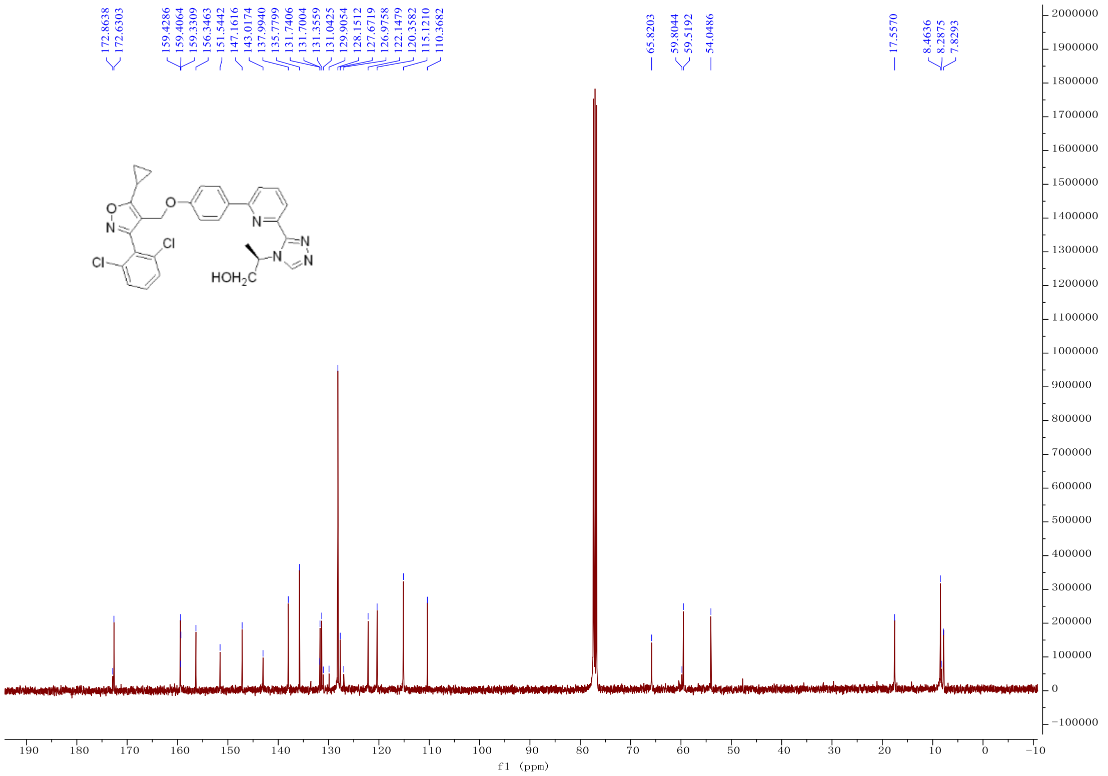

Figure S52: ^{13}C NMR (100 MHz, $CDCl_3$) spectrum of **Z-21**

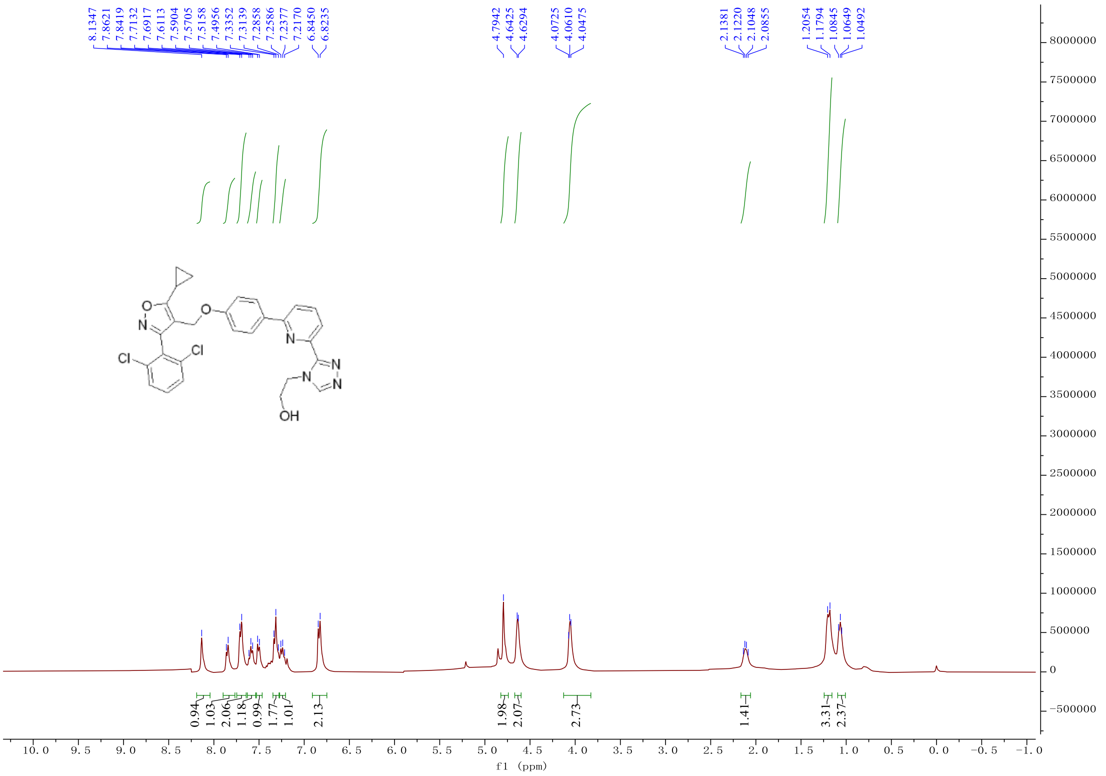

Figure S53: ^{1}H NMR (400 MHz, $CDCl_3$) spectrum of **Z-22**

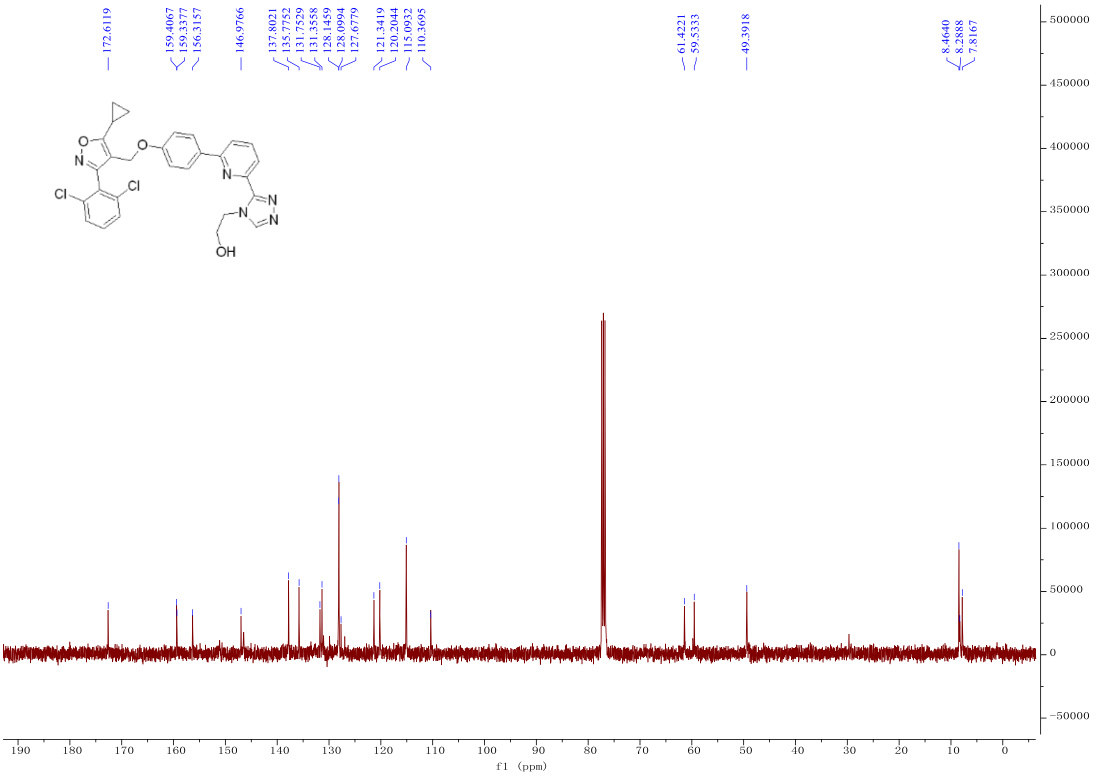

Figure S54: ^{13}C NMR (100 MHz, $CDCl_3$) spectrum of **Z-22**

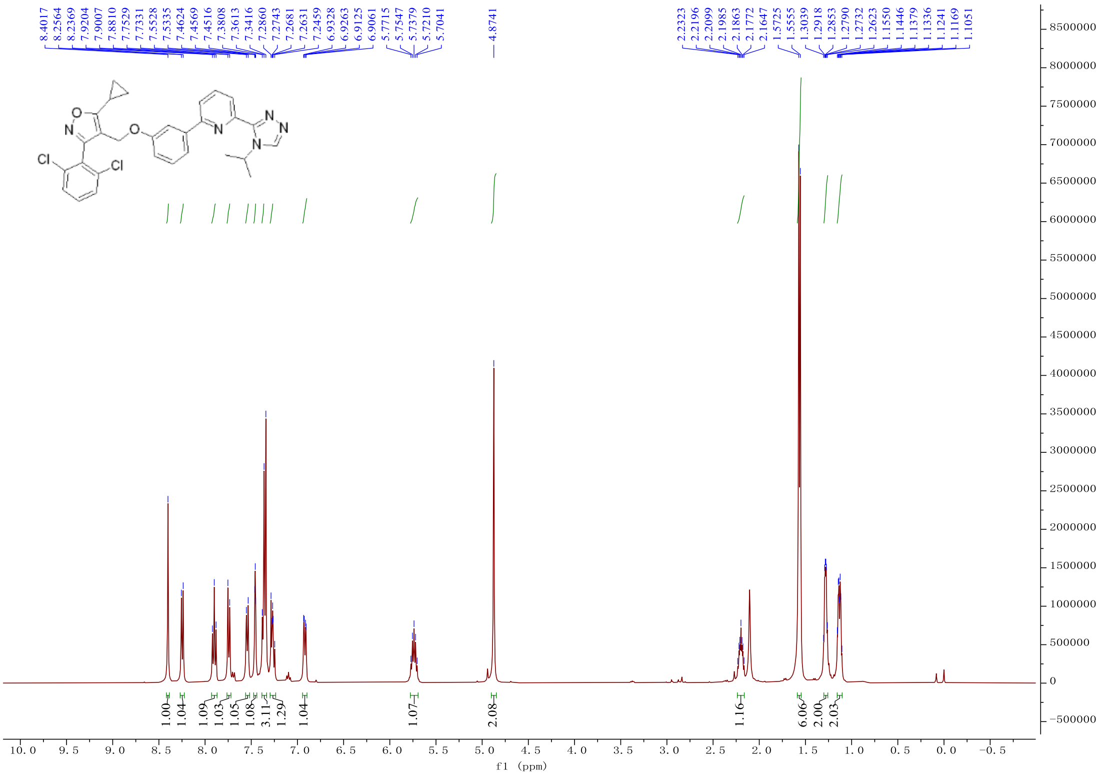

Figure S55: ^{1}H NMR (400 MHz, $CDCl_3$) spectrum of **Z-23**

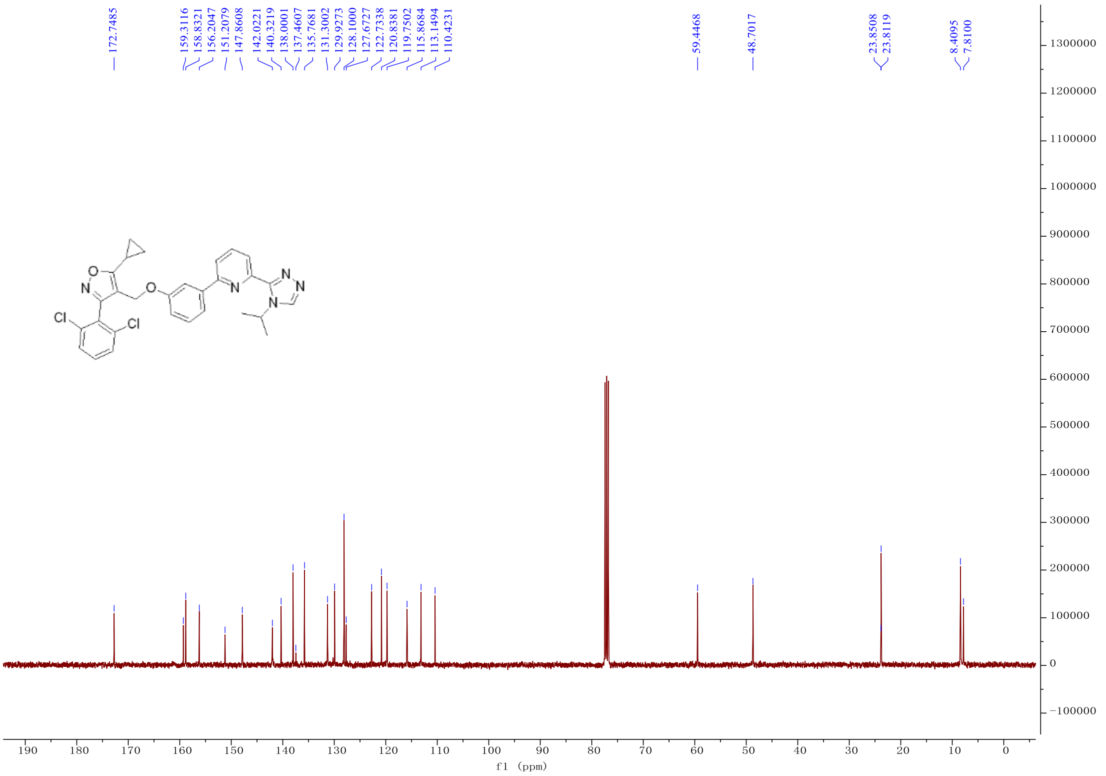

Figure S56: ^{13}C NMR (100 MHz, $CDCl_3$) spectrum of **Z-23**

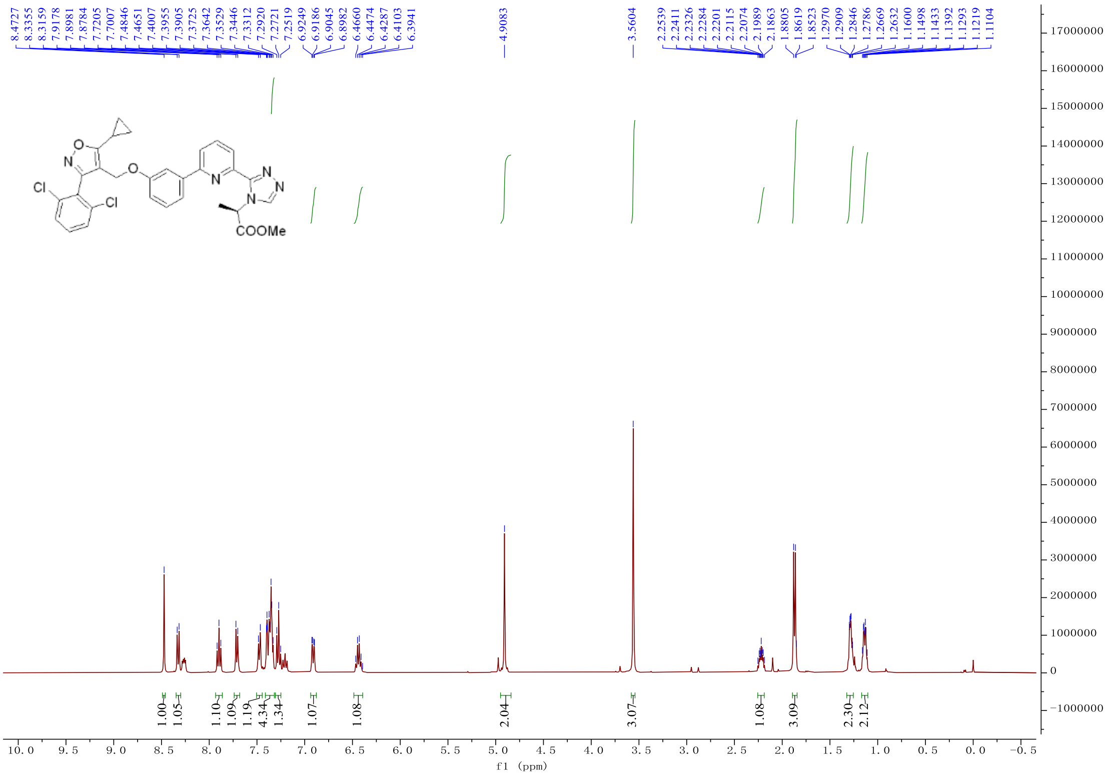

Figure S57: ^{1}H NMR (400 MHz, $CDCl_3$) spectrum of **Z-24**

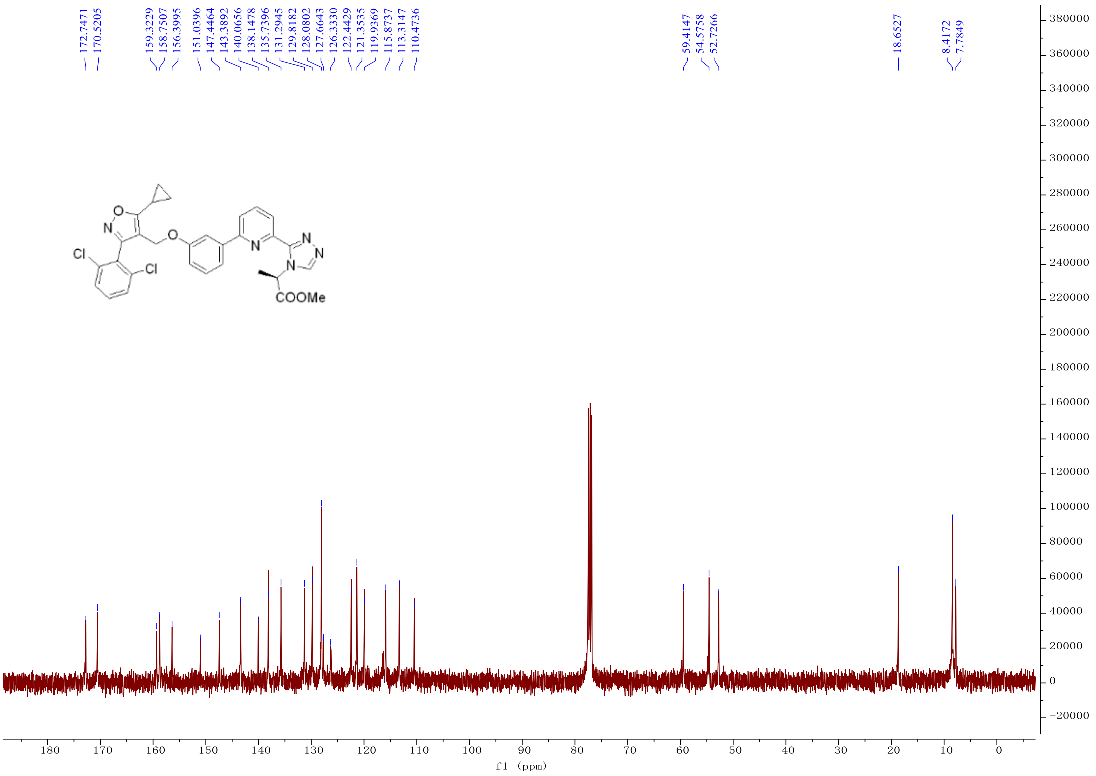

Figure S58: ^{13}C NMR (100 MHz, $CDCl_3$) spectrum of **Z-24**

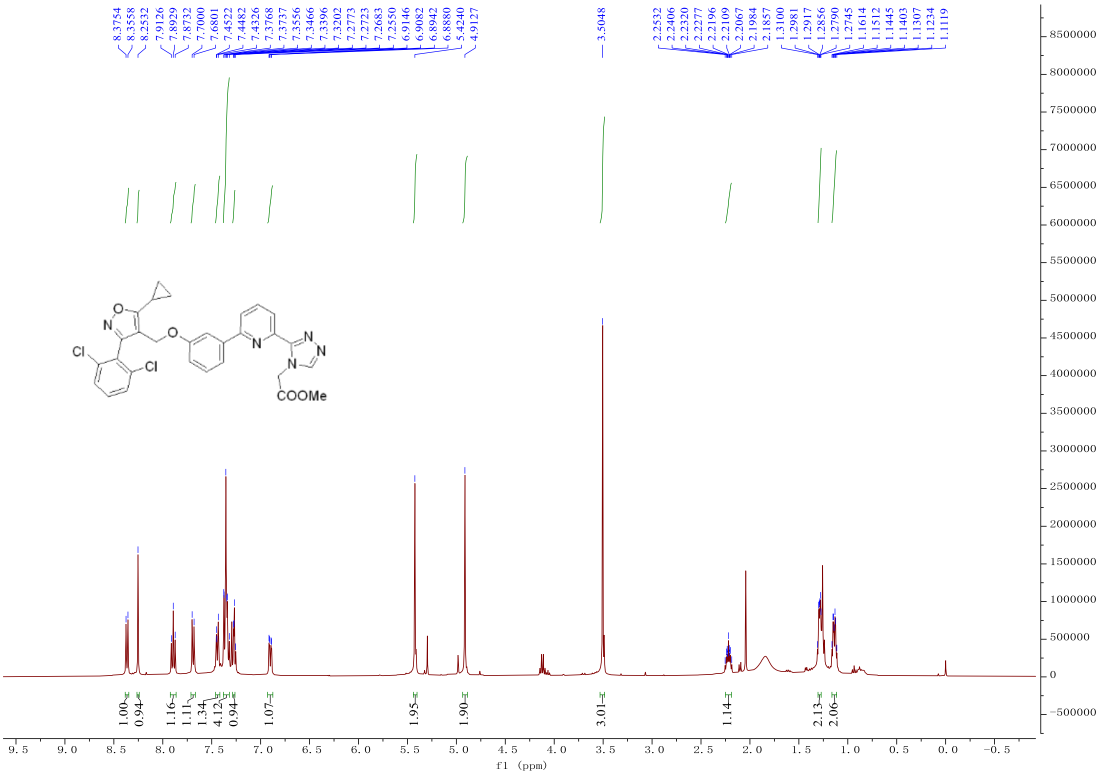

Figure S59: ^{1}H NMR (400 MHz, $CDCl_3$) spectrum of **Z-25**

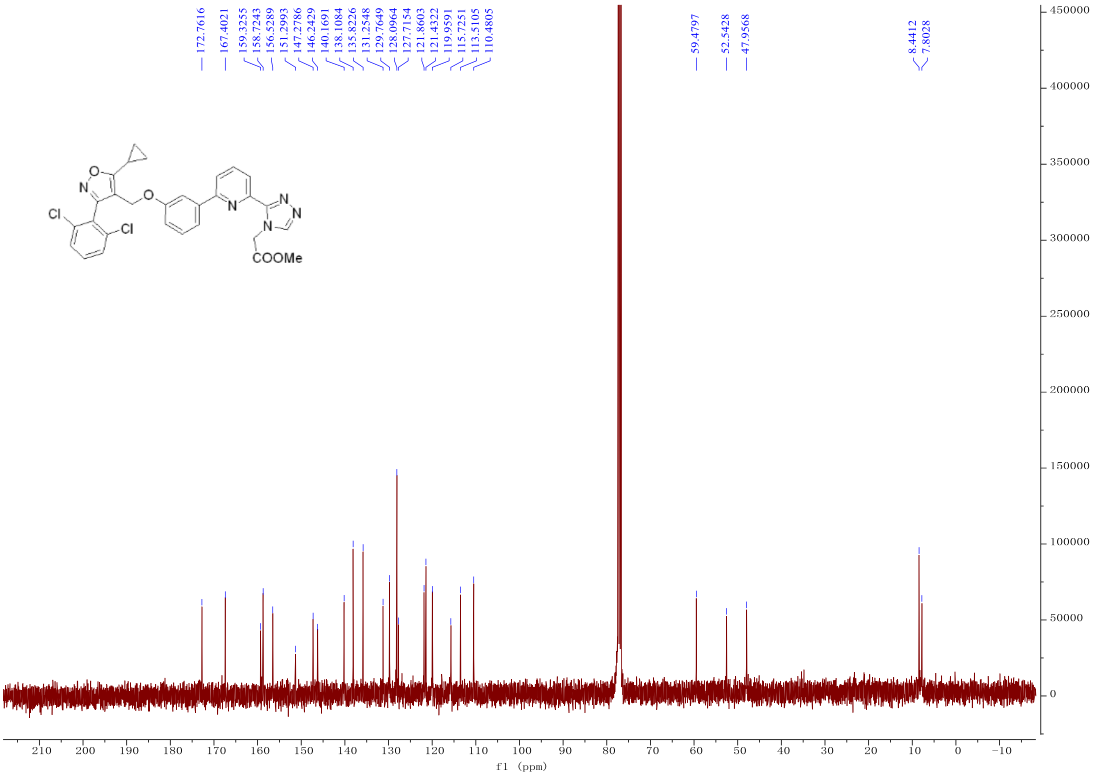

Figure S60: ^{13}C NMR (100 MHz, $CDCl_3$) spectrum of **Z-25**

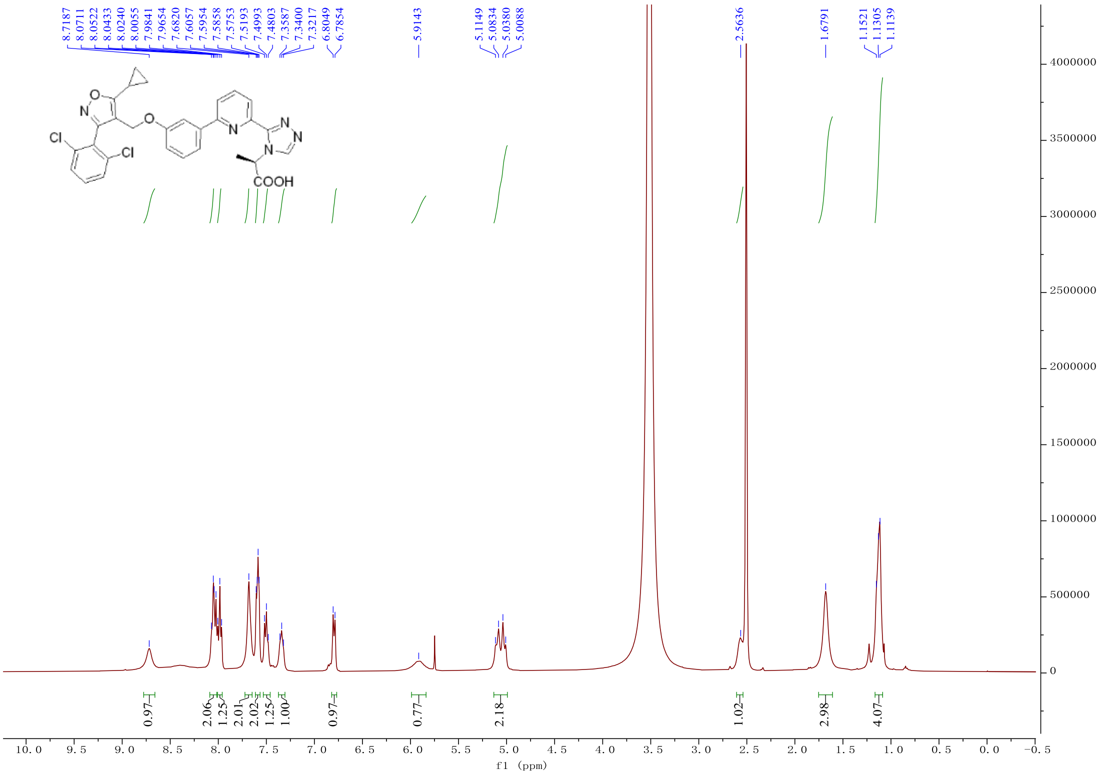

Figure S61: ^{1}H NMR (400 MHz, CDCl$_3$) spectrum of **Z-26**

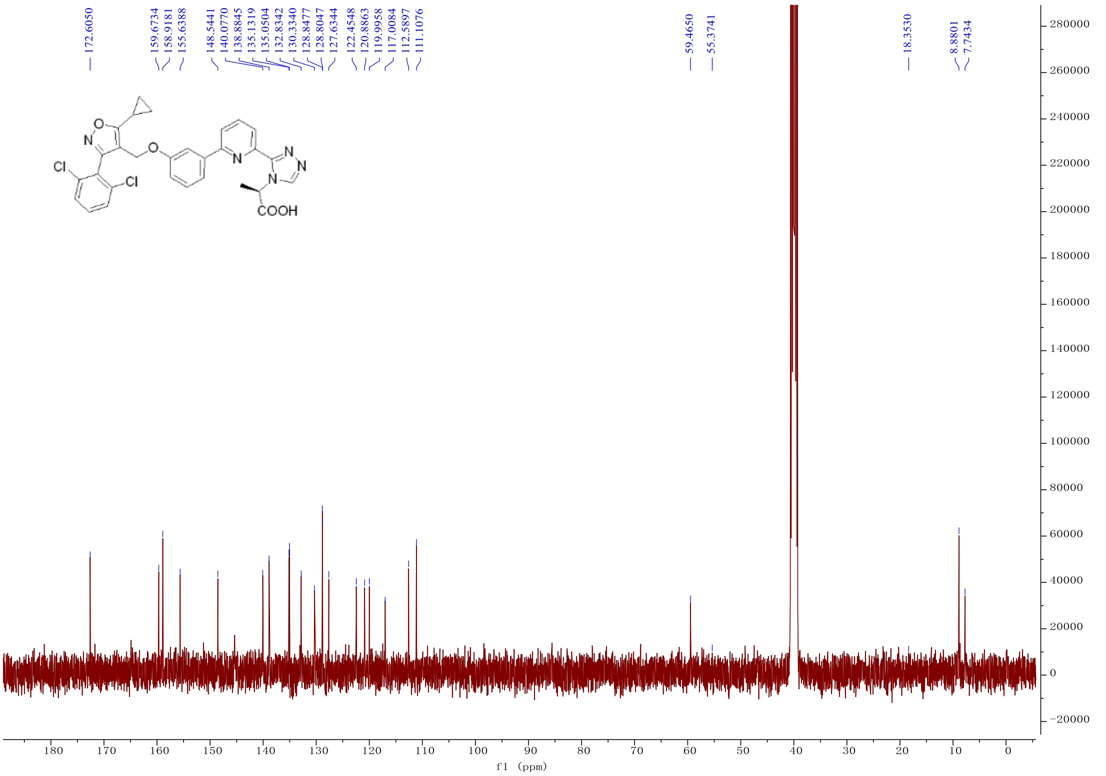

Figure S62: ^{13}C NMR (100 MHz, CDCl$_3$) spectrum of **Z-26**

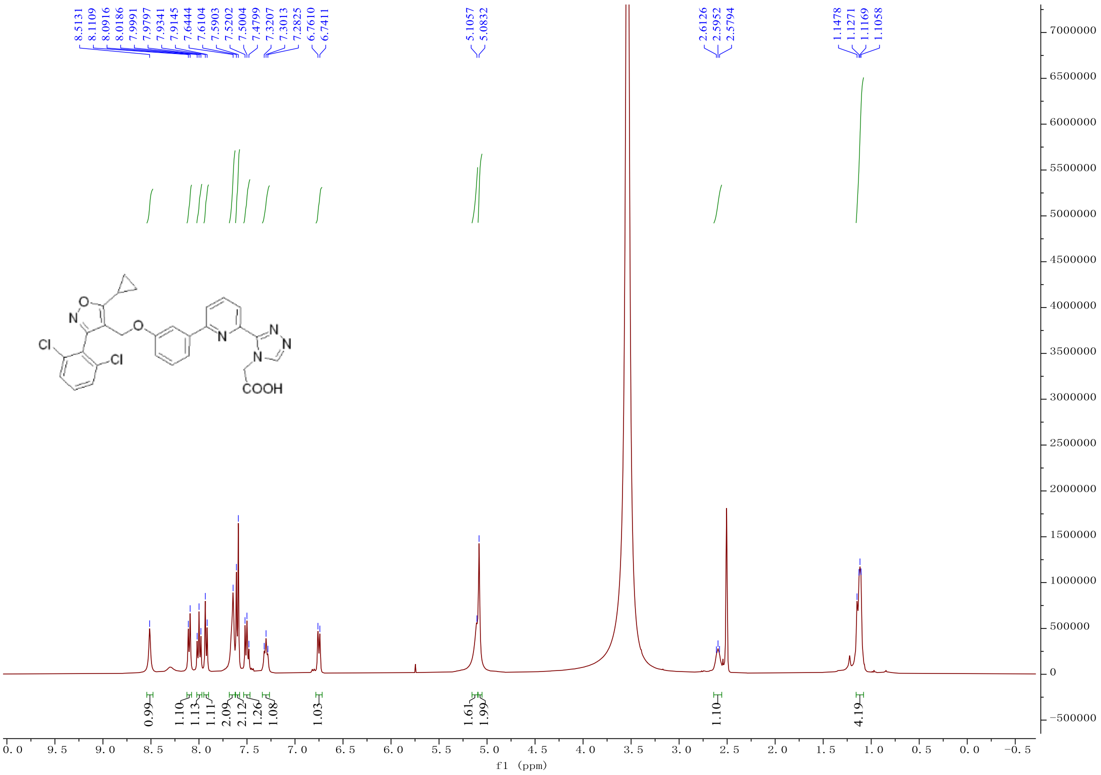

Figure S63: ^{1}H NMR (400 MHz, $CDCl_3$) spectrum of **Z-27**

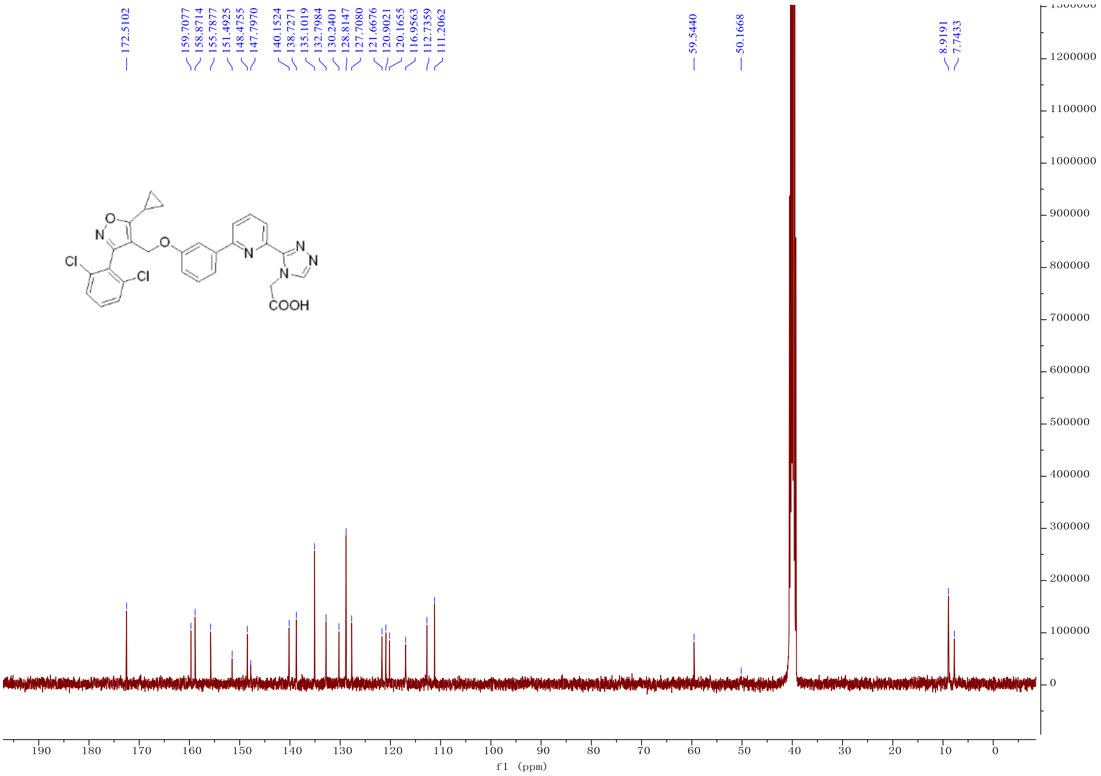

Figure S64: ^{13}C NMR (100 MHz, $CDCl_3$) spectrum of **Z-27**

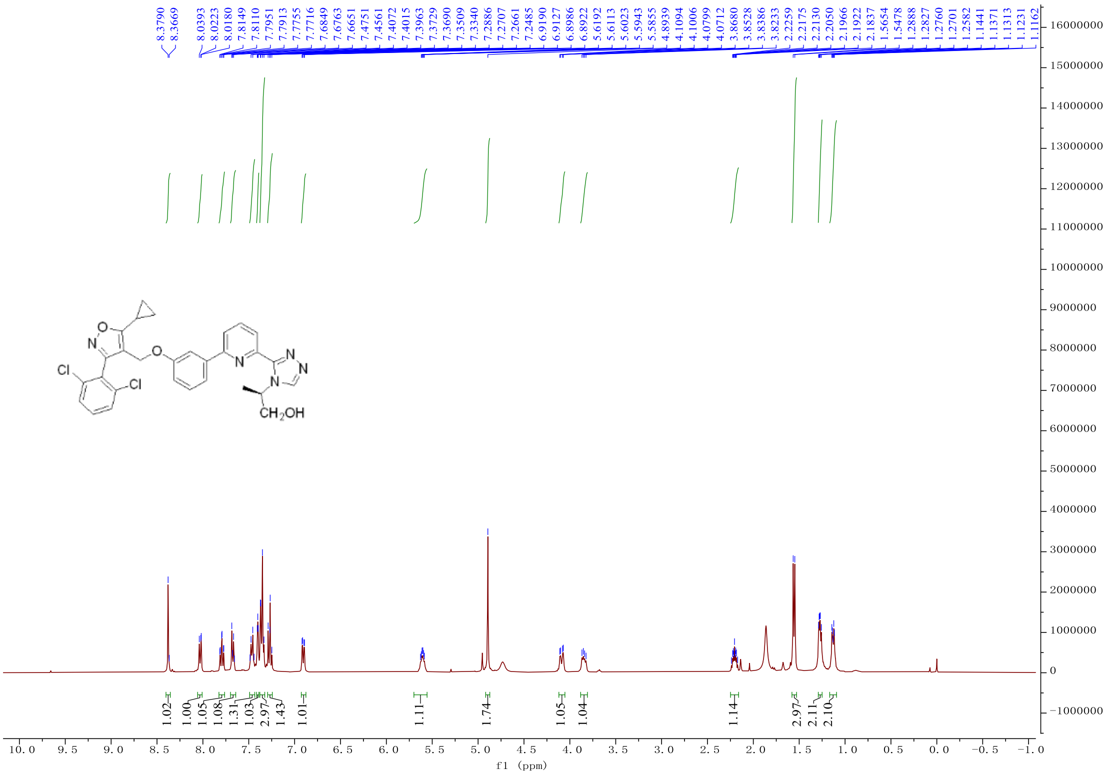

Figure S65: ^{1}H NMR (400 MHz, $CDCl_3$) spectrum of **Z-28**

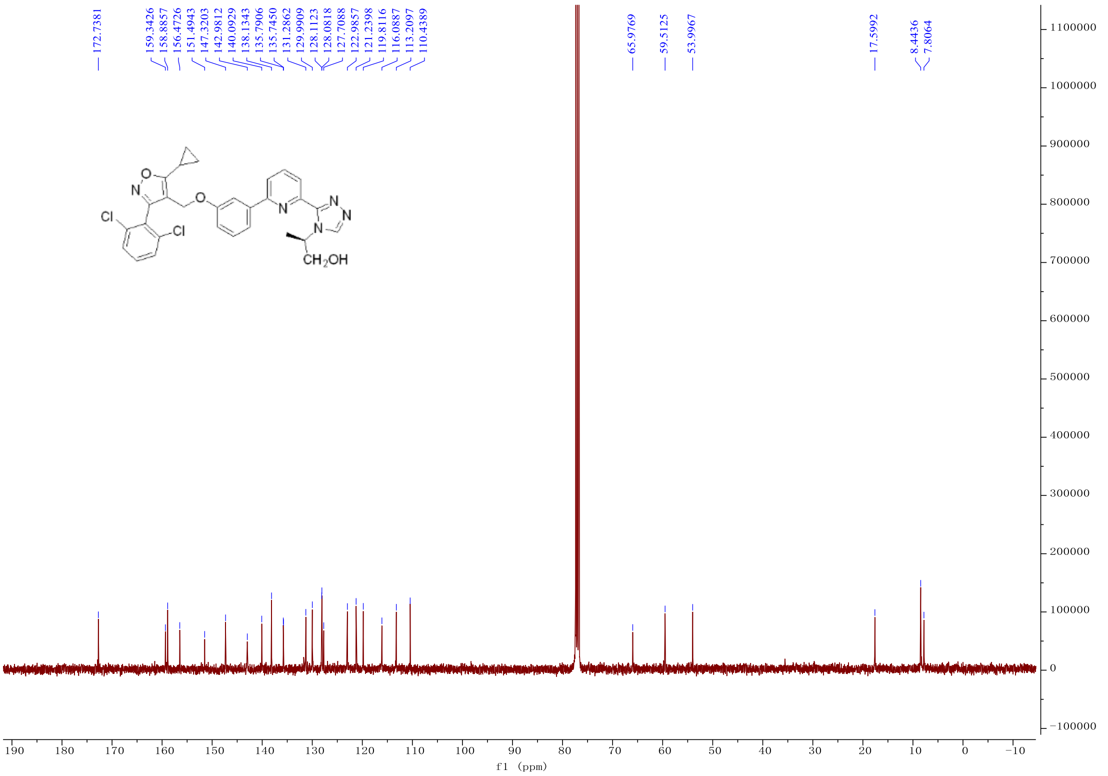

Figure S66: ^{13}C NMR (100 MHz, $CDCl_3$) spectrum of **-Z-28**

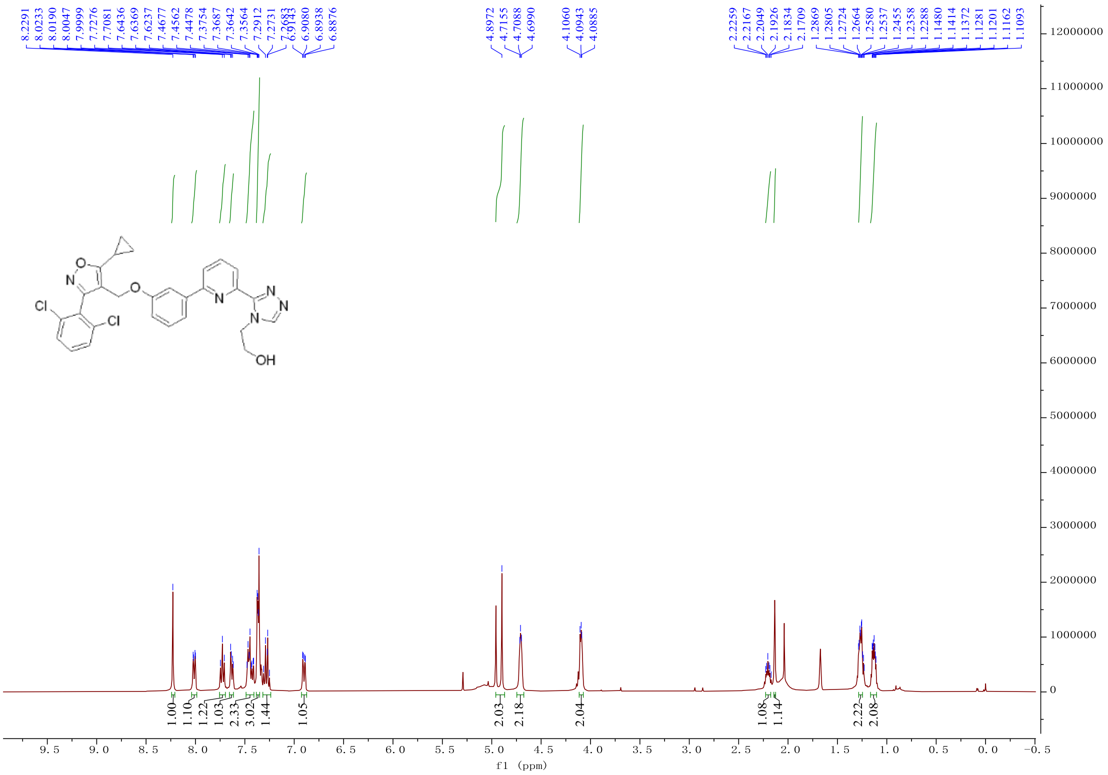

Figure S67: ^{1}H NMR (400 MHz, $CDCl_3$) spectrum of **Z-29**

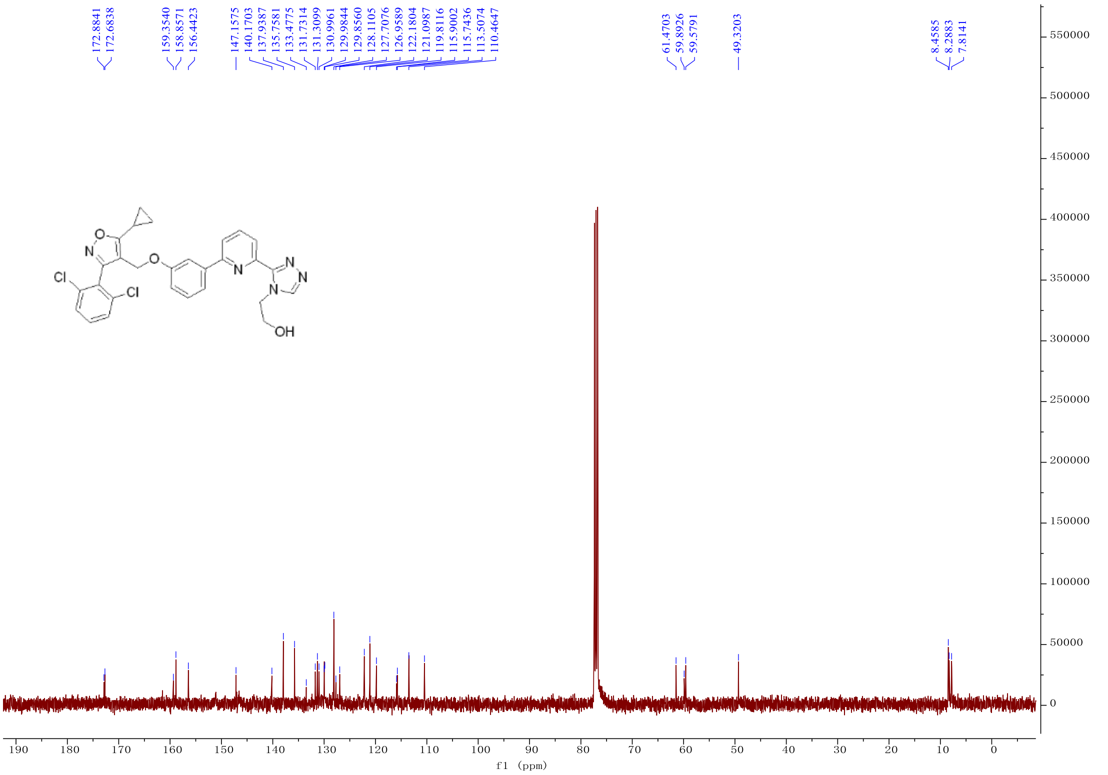

Figure S68: ^{13}C NMR (100 MHz, $CDCl_3$) spectrum of **Z-29**

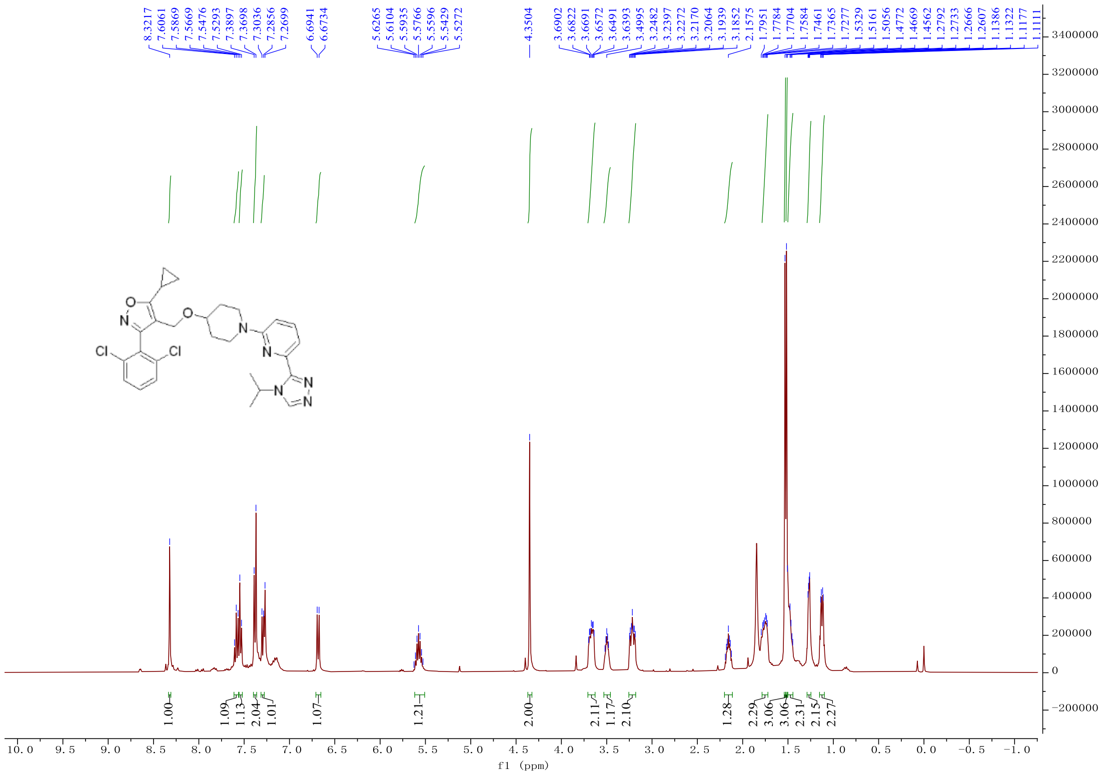

Figure S69: ^{1}H NMR (400 MHz, $CDCl_3$) spectrum of **Z-30**

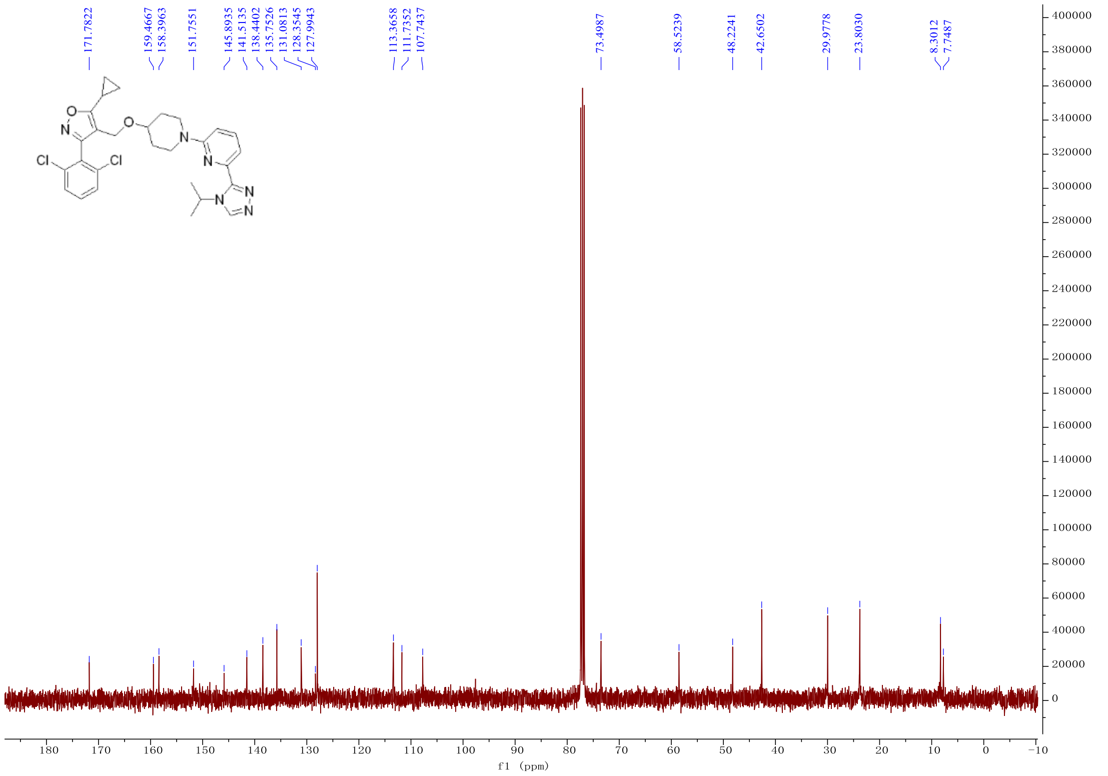

Figure S70: ^{13}C NMR (100 MHz, $CDCl_3$) spectrum of **Z-30**
